# Supplementary material for: Proteomic and Biochemical Analyses of the Mechanism of Tolerance in Mutant Soybean Responding to Flooding Stress
Source: Int J Mol Sci. 2021 Aug 22;22(16):9046. doi: 10.3390/ijms22169046 (PMC8396653; doi:10.3390/ijms22169046)
Supplement: Supplementary file 1 [file ijms-22-09046-s001.zip › Supplemental Figures S1-S5.pdf]

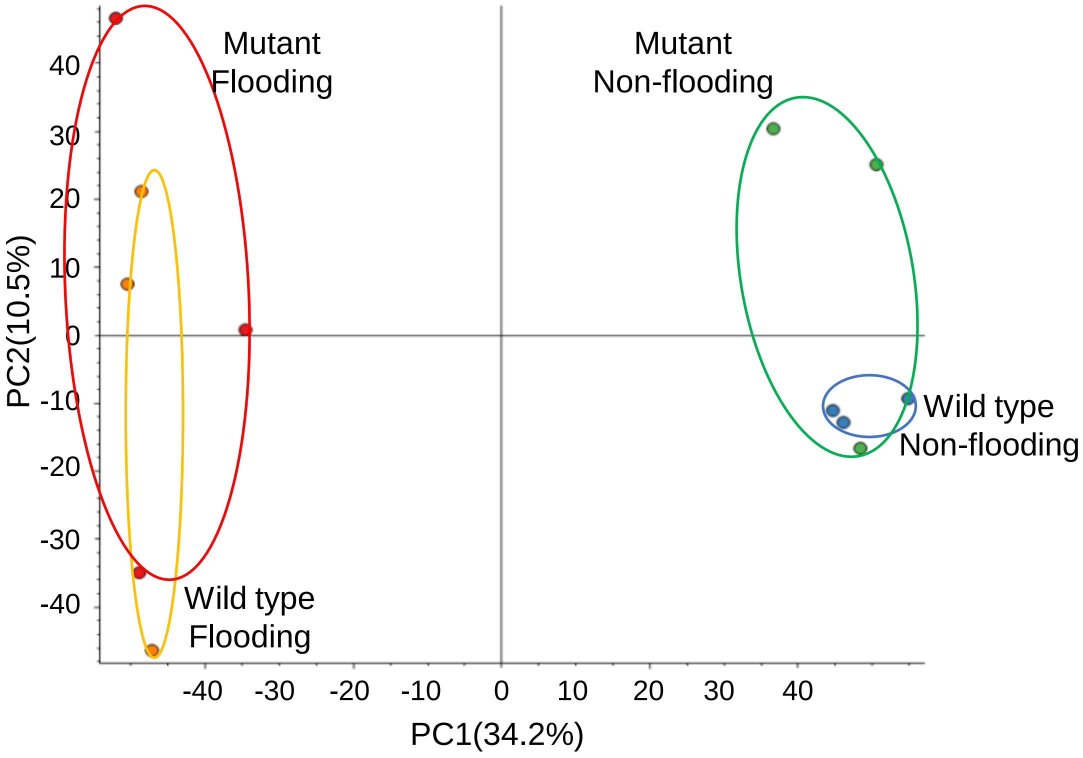

Supplemental Figure S1. Overview of total proteomic data from 12 samples of soybean based on PCA. Two-day-old mutant line and wild type were exposed without (non-flooded) or with (flooded) flooding stress. Proteomic analysis was performed with 3 independent biological replicates for each treatment. PCA was performed with PD 2.2.

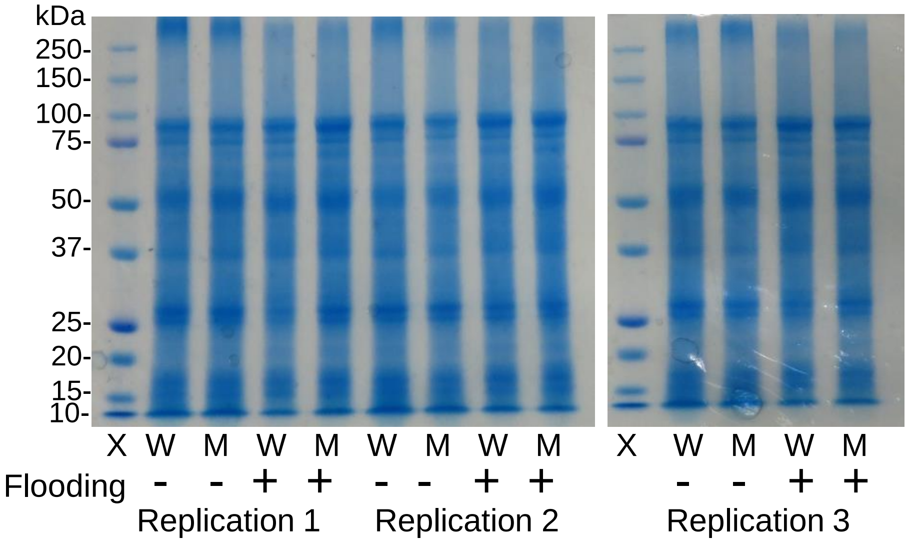

Supplemental Figure S2. The Coomassie brilliant blue staining patterns of proteins used for immuno-blot analysis. Experiments were performed with biologically triplicates for each treatments. Quantified proteins (10 µg) from root including hypocotyl were separated by electrophoresis on a 10% SDS-polyacrylamide. Coomassie brilliant blue staining was used as loading control. “X”, “W”, and “M” means marker proteins, protein pattern of wild type, and protein pattern of mutant line.

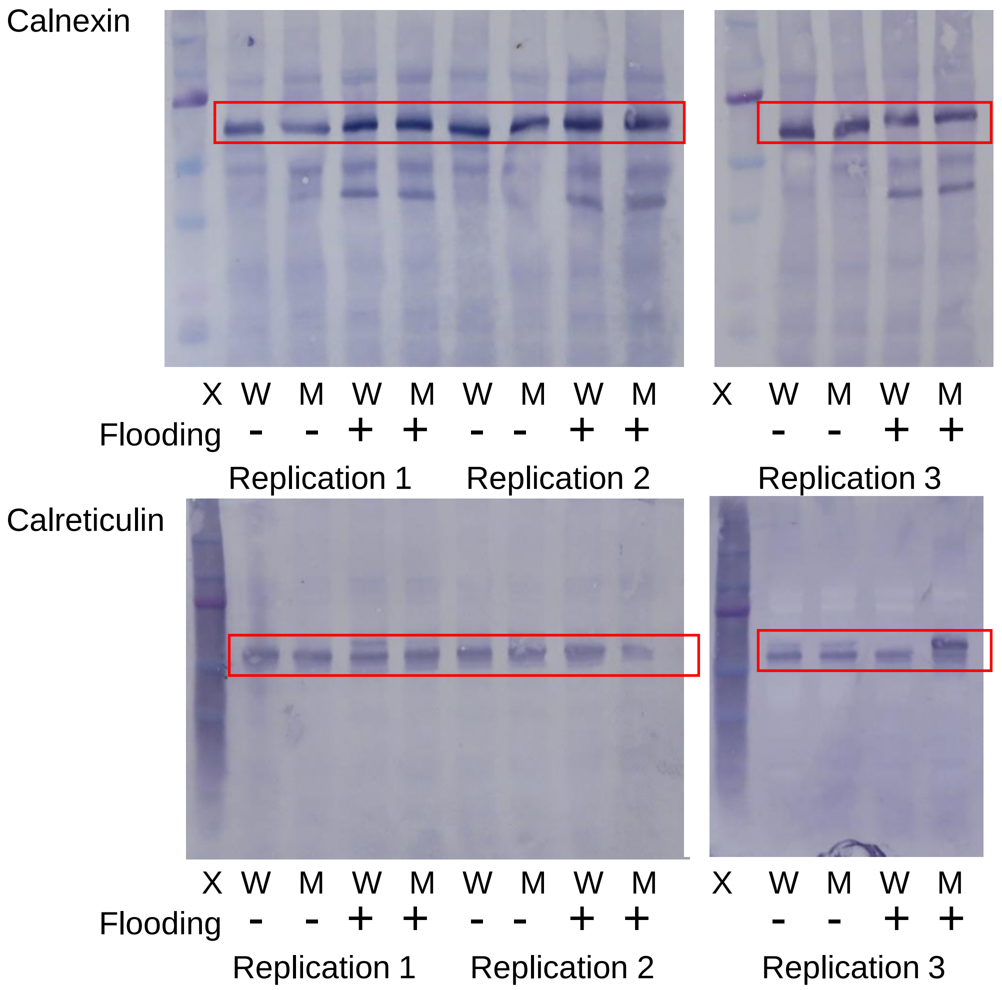

Supplemental Figure S3. Blots of the entire membrane with calnexin and calreticulin antibodies, which used in Figure 4. “X”, “W”, and “M” means marker proteins, wild type, and mutant line.

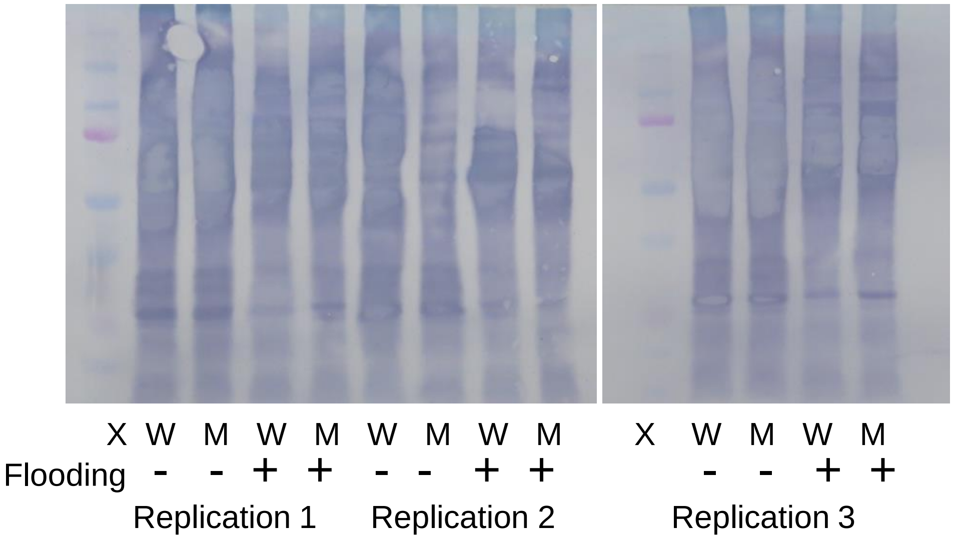

Supplemental Figure S4. Blots of the entire membrane with lectin, which used in Figure 4. “X”, “W”, and “M” means marker proteins, wild type, and mutant line.

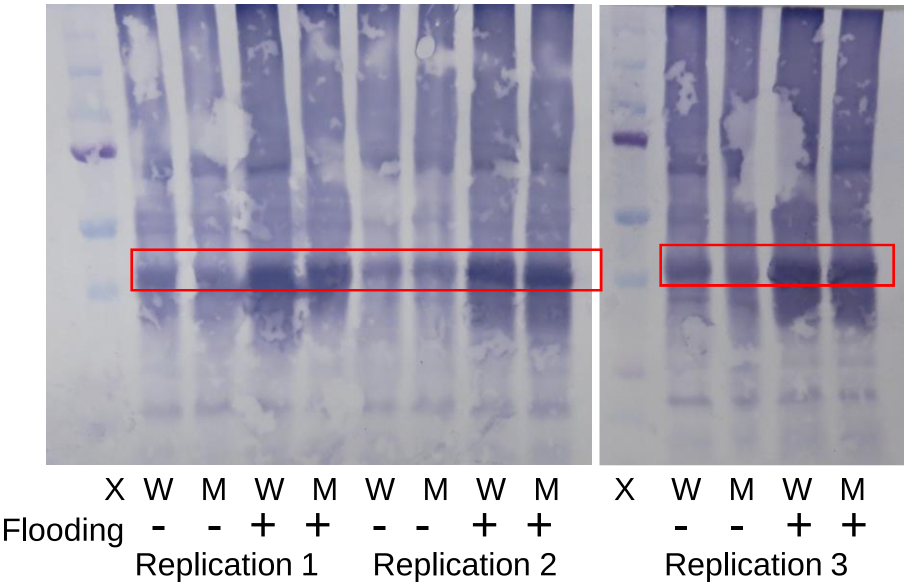

Supplemental Figure S5. Blots of the entire membrane with anti-ADH antibody which used in Figure 5. “X”, “W”, and “M” means marker proteins, wild type, and mutant line.
