# Supplementary material for: Proteomic and Biochemical Analyses of the Mechanism of Tolerance in Mutant Soybean Responding to Flooding Stress
Source: Int J Mol Sci. 2021 Aug 22;22(16):9046. doi: 10.3390/ijms22169046 (PMC8396653; doi:10.3390/ijms22169046)
Supplement: Supplementary file 1 [file ijms-22-09046-s001.zip › Supplementary Tables S1 and S2.pdf]

Supplemental Table S1. List of changed proteins in wild type soybean under flooding compared with non-flooding

Fold change	Accession	Protein names	Cov (%)	MP	MW (kDa)	Calc pI	p-value
11.0852	Q0WX05	Polygalacturonase inhibiting protein	50	10	36.8	8.34	0.0002
9.1702	I1JY29	Uncharacterized protein	42	10	41.2	6.00	0.0019
8.1164	C6TMH1	ZnMc domain-containing protein	12	2	40.1	5.63	0.0002
8.0471	Q42785	Nonsymbiotic hemoglobin	22	3	18.0	8.92	0.0000
7.7919	A0A0R0KF48	Uncharacterized protein	41	14	65.2	6.21	0.0000
7.1804	C6T390	Uncharacterized protein	55	6	16.2	5.91	0.0000
7.0691	I1LJK0	Uncharacterized protein	52	3	7.9	6.60	0.0017
7.0066	I1NF01	Uncharacterized protein	13	8	80.1	6.13	0.0001
6.9769	A0A0R0GEW3	Uncharacterized protein	11	3	30.6	6.80	0.0001
6.6448	I1LL87	Uncharacterized protein	18	2	19.3	4.96	0.0011
6.5690	I1L3I1	Lectin_legB domain-containing protein	12	3	33.2	5.10	0.0003
6.4617	I1K9M3	Uncharacterized protein	21	7	73.0	5.30	0.0001
6.3329	C6TMB5	Uncharacterized protein	45	5	17.8	8.97	0.0011
6.1785	C6TMH8	Ribosomal protein L19	21	2	24.7	11.44	0.0001
6.0694	I1MV69	Uncharacterized protein	25	4	27.5	9.20	0.0019
5.8079	I1KJ00	Uncharacterized protein	14	3	28.2	5.43	0.0020
5.7866	I1KR99	LRRNT_2 domain-containing protein	16	3	37.4	7.91	0.0026
5.7015	C6TFF5	Uncharacterized protein	26	2	11.1	10.45	0.0003
5.5797	C6T229	Uncharacterized protein	32	2	8.0	8.84	0.0008
5.5748	C6T9K9	Elongation factor Tu	49	17	49.8	7.05	0.0003
5.5168	A0A0R0IID0	Uncharacterized protein	31	4	24.0	6.02	0.0031
5.4166	C6TDV4	LRRNT_2 domain-containing protein	7	2	36.2	8.43	0.0004
5.2223	Q0WX04	Polygalacturonase inhibiting protein	19	3	37.5	8.91	0.0010
5.0100	A0A0R0K553	Fe2OG dioxygenase domain-containing protein	38	8	34.8	5.58	0.0003
4.8041	I1KZW3	Uncharacterized protein	32	6	32.8	5.92	0.0005
4.6002	C6SYA5	60S ribosomal protein L18a	29	4	21.3	10.42	0.0004
4.4636	I1LC49	Peptidase A1 domain-containing protein	18	4	47.3	8.24	0.0051
4.3081	I1LT24	DUF3700 domain-containing protein	33	6	28.0	6.68	0.0001
4.2615	K7MID0	Uncharacterized protein	8	4	96.9	6.39	0.0000
4.2281	I1K9W9	Uncharacterized protein	14	2	34.7	8.54	0.0023
4.1308	A0A0R4J4G7	Uncharacterized protein	15	2	29.4	6.79	0.0010
4.1117	I1KBD1	Protein kinase domain-containing protein	4	2	129.2	6.74	0.0057
4.0226	C6TC19	Rhodanese domain-containing protein	13	2	16.4	8.25	0.0020
3.9660	I1K467	Uncharacterized protein	50	18	59.0	6.51	0.0002
3.9646	I1KNR7	Uncharacterized protein	3	2	81.5	6.37	0.0486
3.9423	I1NF42	Uncharacterized protein	17	5	43.5	4.84	0.0011
3.9361	I1KP32	UVR domain-containing protein	39	23	102.1	6.80	0.0034
3.8956	I1K657	RPN13_C domain-containing protein	35	5	36.1	4.44	0.0325
3.8932	A0A0R4J4H3	Nascent polypeptide-associated complex subunit beta	67	11	17.7	7.40	0.0034
3.8755	I1KMK2	Uncharacterized protein	4	3	162.5	8.13	0.0067
3.8491	C6THW8	DUF3700 domain-containing protein	40	7	28.0	6.61	0.0000
3.8122	I1N846	Uncharacterized protein	27	11	83.9	6.73	0.0088
3.8117	I1JQU3	AMP-binding domain-containing protein	6	2	74.4	7.18	0.0022
3.7880	K7LSP6	WD_REPEATS_REGION domain-containing protein	7	2	37.2	5.10	0.0086
3.7775	C6TLA6	DUF3700 domain-containing protein	20	3	27.5	7.37	0.0001
3.7581	C6T721	Aldose 1-epimerase	13	3	40.6	9.28	0.0031
3.6824	I1J8C9	Rab proteins geranylgeranyl transferase component	13	3	60.6	4.87	0.0074
3.6663	I1MQG3	Uncharacterized protein	28	3	22.2	5.21	0.0165
3.5975	C6TKX3	Uncharacterized protein	8	2	27.2	6.40	0.0348
3.5068	K7MFQ7	Uncharacterized protein	15	6	68.8	8.50	0.0003
3.5011	I1JS15	Uncharacterized protein	5	3	116.8	6.58	0.0495
3.4511	I1MAE6	PKS_ER domain-containing protein	39	11	40.8	6.52	0.0003

3.3270	I1LVI3	Uncharacterized protein	12	9	100.1	9.07	0.0082
3.3245	Q948X9	Beta-conglycinin alpha-subunit	7	3	72.4	5.40	0.0099
3.3153	I1ME27	Uncharacterized protein	3	2	140.8	6.15	0.0068
3.3099	C6TCK6	Uncharacterized protein	13	2	25.8	6.86	0.0005
3.2972	C6SXR2	Ribosomal protein L19	21	2	24.6	11.44	0.0052
3.2893	I1J6C3	Arf-GAP domain-containing protein	34	8	43.2	8.92	0.0013
3.2155	I1KQE2	Uncharacterized protein	59	9	34.6	7.81	0.0230
3.1936	A0A0R4J594	Peroxidase (EC 1.11.1.7)	24	5	34.8	7.90	0.0002
3.1859	Q42447	Maturation protein (Maturation-associated protein)	27	3	25.6	6.52	0.0106
3.1785	A0A0R0FZI3	AB hydrolase-1 domain-containing protein	9	2	33.6	6.54	0.0097
3.1366	P11827	Beta-conglycinin alpha' subunit	18	8	72.2	5.71	0.0091
3.1003	K7MNG2	Cytosolic Fe-S cluster assembly factor NBP35	30	6	38.2	4.84	0.0000
3.0608	K7KRK2	Uncharacterized protein	17	3	31.5	6.44	0.0083
2.9956	I1MBS4	Usp domain-containing protein	27	3	18.0	9.41	0.0120
2.9562	I1M7P3	Uncharacterized protein	34	2	14.7	4.93	0.0001
2.9492	I1LRQ2	NEDD8-activating enzyme E1 regulatory subunit	21	5	57.9	5.24	0.0045
2.9418	Q9SEL0	Seed maturation protein PM24	20	3	26.8	5.33	0.0043
2.9249	I1NCN7	Uncharacterized protein	5	3	115.7	6.46	0.0042
2.9114	I1LVS8	Xyloglucan endotransglucosylase/hydrolase	10	2	33.2	8.02	0.0055
2.9062	K7KSU9	GYF domain-containing protein	2	2	197.7	6.47	0.0353
2.9043	A0A024BT07	Acyl-[acyl-carrier-protein] desaturase	12	4	44.0	7.23	0.0000
2.8734	Q01527	Maturation protein	38	2	15.6	9.66	0.0013
2.8361	C6TKW5	MHD domain-containing protein	7	2	49.7	7.18	0.0092
2.8335	P26413	Heat shock 70 kDa protein	53	25	70.8	5.49	0.0026
2.8313	A0A0R4J364	Uncharacterized protein	67	16	40.9	6.40	0.0008
2.7764	K7LSZ2	AA_TRNA_LIGASE_II domain-containing protein	15	5	62.8	6.49	0.0499
2.7744	A0A0G4DBR5	UDP-glycosyltransferase 79B30	10	2	51.1	5.78	0.0049
2.7738	I1KQV7	Uncharacterized protein	38	19	72.5	6.04	0.0201
2.7331	P51061	Phosphoenolpyruvate carboxylase	52	39	110.7	6.47	0.0316
2.7146	I1MN40	VWFA domain-containing protein	15	3	42.4	4.73	0.0022
2.7038	I1M138	Peptidase A1 domain-containing protein	10	3	51.2	8.00	0.0000
2.6976	I1K0A8	Uncharacterized protein	28	9	48.5	5.40	0.0320
2.6834	I1L1N3	Uncharacterized protein	10	2	25.2	5.63	0.0007
2.6484	A0A0R0KSG7	Ribosomal protein L19	17	2	24.7	11.40	0.0033
2.6462	C6TDA6	Protein kinase domain-containing protein	7	2	50.9	4.54	0.0042
2.6432	I1JG72	PlsC domain-containing protein	8	2	43.2	9.36	0.0412
2.6401	I1JJ62	Citrate synthase (EC 2.3.3.16)	19	5	56.5	8.72	0.0416
2.6183	Q9ZTY1	35 kDa seed maturation protein	10	2	35.3	6.32	0.0004
2.6044	A0A0R0KXL5	Uncharacterized protein	23	2	13.6	8.18	0.0000
2.5993	K7MEI2	Exocyst subunit Exo70 family protein	8	2	75.6	6.00	0.0126
2.5586	I1MUP6	Uncharacterized protein	47	14	46.4	5.11	0.0001
2.5507	A0A0B5E8W0	Tau class glutathione S-transferase	28	4	25.9	6.15	0.0367
2.5316	A0A0R4J2I4	Uncharacterized protein	43	16	65.6	5.94	0.0005
2.5243	I1N627	PHB domain-containing protein	26	6	31.5	5.24	0.0081
2.4710	C6TB96	Uncharacterized protein	22	5	35.2	5.49	0.0005
2.4707	A0A0R0ENW6	Peptidase A1 domain-containing protein	35	8	46.3	8.53	0.0149
2.4705	I1JKK8	SEC7 domain-containing protein	9	11	198.7	5.66	0.0079
2.4531	I1MWJ3	Uncharacterized protein	19	2	19.0	9.48	0.0151
2.4432	I1MR73	Uncharacterized protein	4	2	58.0	8.90	0.0065
2.4345	I1KLU0	Mitogen-activated protein kinase (EC 2.7.11.24)	27	7	49.9	5.96	0.0378
2.4337	C6TEY7	Ubiquitin thioesterase (EC 3.4.19.12)	39	6	33.8	4.91	0.0002
2.4160	I1MWZ7	Aldedh domain-containing protein	16	5	53.2	7.61	0.0138
2.3960	I1MCF0	Uncharacterized protein	23	5	45.2	6.43	0.0024
2.3355	I1K7R0	Uncharacterized protein	27	3	16.1	5.35	0.0028
2.3231	K7KU37	Uncharacterized protein	2	2	147.2	5.94	0.0051
2.3132	I1MEX0	MPN domain-containing protein	57	8	34.6	6.71	0.0000
2.2754	C6T545	Uncharacterized protein	32	2	14.9	9.47	0.0494

2.2593	C6TME1	Uncharacterized protein	32	9	35.8	9.58	0.0001
2.2067	I1L7R7	CTP synthase (EC 6.3.4.2) (UTP--ammonia ligase)	9	3	61.6	6.05	0.0003
2.1869	K7MVZ6	Glucose-6-phosphate isomerase (EC 5.3.1.9)	37	10	62.7	5.72	0.0014
2.1606	C6TN86	Uncharacterized protein	10	2	38.3	6.00	0.0087
2.1304	I1LUZ1	Uncharacterized protein	11	3	64.0	5.53	0.0001
2.1301	A0A0R0ETL8	Uncharacterized protein	33	13	64.3	8.15	0.0464
2.1169	I1K586	Protein kinase domain-containing protein	3	2	123.3	4.93	0.0170
2.1117	I1N0X9	Uncharacterized protein	5	3	118.3	6.54	0.0047
2.0934	K7LDT9	Uncharacterized protein	9	5	103.3	6.76	0.0005
2.0925	I1MEH8	Uncharacterized protein	41	6	22.2	7.20	0.0000
2.0884	I1LC12	SHSP domain-containing protein	30	4	22.4	6.02	0.0038
2.0805	I1J8S7	Uncharacterized protein	46	7	29.3	6.73	0.0028
2.0792	I1JCQ0	Amino oxidase domain-containing protein	11	4	60.8	6.90	0.0040
2.0723	P04670	Uricase-2 isozyme 1	46	12	35.1	7.97	0.0006
2.0700	K7LG16	Uncharacterized protein	7	3	109.1	5.67	0.0045
2.0688	I1MQK0	Uncharacterized protein	16	3	41.7	5.78	0.0007
2.0649	K7LF33	RBR-type E3 ubiquitin transferase (EC 2.3.2.31)	10	3	68.2	5.26	0.0006
2.0629	K7KNK3	Uncharacterized protein	26	2	11.2	10.96	0.0057
2.0577	I1KU42	Uncharacterized protein	3	2	91.4	6.23	0.0319
2.0372	C6TFW0	Uncharacterized protein	37	3	13.3	9.16	0.0007
2.0333	I1N7M0	RIBOSOMAL_L9 domain-containing protein	20	2	25.6	9.58	0.0167
2.0297	K7L1E9	Fe2OG dioxygenase domain-containing protein	15	3	34.7	5.71	0.0004
2.0037	I1MRW2	Importin N-terminal domain-containing protein	5	4	123.7	4.83	0.0002
1.9896	I1L7V7	Usp domain-containing protein	30	2	17.2	7.87	0.0046
1.9880	A0A0R0IH39	Uncharacterized protein	2	2	212.1	7.31	0.0013
1.9790	C6SVT0	Usp domain-containing protein	42	6	18.0	7.15	0.0002
1.9635	K7MDW5	Uncharacterized protein	14	20	247.4	5.91	0.0138
1.9303	K7M034	BED-type domain-containing protein	8	6	122.7	5.00	0.0014
1.9238	K7MPT4	TPR_REGION domain-containing protein	6	2	59.8	7.37	0.0015
1.9153	A0A0R0KE56	Pectinesterase (EC 3.1.1.11)	27	5	32.2	8.70	0.0015
1.8894	I1KUE9	WD_REPEATS_REGION domain-containing protein	10	2	38.8	8.22	0.0034
1.8374	C6SVM2	Uncharacterized protein	27	2	17.4	8.94	0.0014
1.7842	C6K8D1	Seed biotinylated protein 68 kDa isoform	23	9	67.9	6.58	0.0000
1.7762	I1L3K7	Uncharacterized protein	64	17	48.0	6.29	0.0000
1.7754	A0A0R0JDS4	TP_methylase domain-containing protein	24	2	30.8	5.39	0.0164
1.7692	C6TNN5	ZnMc domain-containing protein	29	7	43.4	6.10	0.0003
1.7635	C6T063	ANK_REP_REGION domain-containing protein	36	2	16.8	4.63	0.0296
1.7312	I1LM96	60S ribosomal export protein NMD3	8	3	58.5	6.73	0.0057
1.7243	I1LDX7	Uncharacterized protein	7	3	84.6	8.69	0.0110
1.7114	K7K676	AP-1 complex subunit gamma	21	10	95.5	6.51	0.0058
1.7109	I1J9W0	40S ribosomal protein S30	11	2	11.6	12.07	0.0020
1.7070	I1K301	Uncharacterized protein	59	7	14.8	5.71	0.0015
1.6980	I1M1V8	Uncharacterized protein	56	18	63.4	6.11	0.0001
1.6830	A0A0R0H3L4	Poly [ADP-ribose] polymerase	40	21	91.6	5.59	0.0005
1.6761	K7KNP4	Uncharacterized protein	6	8	213.8	6.13	0.0019
1.6701	I1MY42	Uncharacterized protein	2	2	135.6	7.58	0.0322
1.6539	A0A0R0F3E0	Histone H2A	21	2	15.5	10.71	0.0019
1.6363	K7KUE8	DFP domain-containing protein	13	4	36.4	7.94	0.0497
1.6352	K7MFC0	GYF domain-containing protein	5	7	179.2	6.23	0.0005
1.6333	Q8RVH5	Basic 7S globulin 2	40	8	47.2	8.25	0.0188
1.6318	I1LWI3	PHB domain-containing protein	29	7	31.5	5.24	0.0029
1.6277	A0A0R4J3M4	Uncharacterized protein	41	15	65.1	6.27	0.0009
1.6203	C6TIP6	Uncharacterized protein	10	3	54.5	8.25	0.0470
1.6199	K7M5G4	GYF domain-containing protein	3	3	205.6	6.46	0.0129
1.6118	K7KGC7	Sucrose synthase (EC 2.4.1.13)	41	31	92.2	6.13	0.0007
1.5894	K7MZJ0	Sucrose synthase (EC 2.4.1.13)	47	33	92.2	6.38	0.0000

1.5794	C6TE39	Ubiquinone biosynthesis O-methyltransferase	18	3	31.8	6.44	0.0090
1.5789	A0A0R4J387	Chalcone-flavonone isomerase family protein	62	8	23.5	5.00	0.0020
1.5765	I1KR17	Uncharacterized protein	21	3	32.5	8.76	0.0007
1.5345	C6TEE9	Uncharacterized protein	55	10	30.4	6.09	0.0001
1.5315	I1JIE0	Ferritin (EC 1.16.3.1)	38	8	28.1	6.89	0.0041
1.5218	I1K9P4	ZF-HD dimerization-type domain-containing protein	18	2	32.4	6.52	0.0081
1.5174	K7LKJ0	HMA domain-containing protein	48	3	8.6	6.60	0.0016
1.5173	A0A0R0ELP2	Lipase_GDSL domain-containing protein	17	4	43.7	8.60	0.0431
1.5024	I1NFP9	PB1 domain-containing protein	19	6	78.2	6.30	0.0056
1.4970	C6SW77	Uncharacterized protein	34	5	25.8	9.69	0.0065
1.4876	I1K964	Proteasome subunit beta (EC 3.4.25.1)	78	11	25.1	5.55	0.0045
1.4810	I1NA37	Uncharacterized protein	35	4	21.0	6.93	0.0081
1.4781	A0A0R0GAQ3	WD_REPEATS_REGION domain-containing protein	18	5	52.5	8.91	0.0222
1.4710	A0A368UH26	Uncharacterized protein	17	2	21.8	6.01	0.0089
1.4696	I1JET8	Uncharacterized protein	6	2	56.9	6.48	0.0279
1.4681	I1N7K5	UBX domain-containing protein	20	2	29.1	7.18	0.0157
1.4581	Q9SWB2	Seed maturation protein PM41	31	2	8.2	4.97	0.0415
1.4576	C6TN05	Hypersensitive induced reaction protein 4	12	2	32.6	5.31	0.0134
1.4419	Q9XET0	Seed maturation protein PM30	30	4	15.1	8.90	0.0000
1.4330	A0A0R0J2N5	Uncharacterized protein	10	2	29.7	6.73	0.0075
1.4321	I1MGH2	Fn3_like domain-containing protein	13	7	84.0	8.15	0.0230
1.4321	A0A0R0F259	Uncharacterized protein	41	14	65.2	6.21	0.0140
1.4166	A0A0R0EPA1	Uncharacterized protein	16	2	30.5	9.09	0.0262
1.3963	I1JU00	SHSP domain-containing protein	15	2	17.9	7.18	0.0004
1.3755	I1L8X8	DUF5311 domain-containing protein	2	2	169.3	5.87	0.0006
1.3681	A0A0R4J2Q1	Peptidase A1 domain-containing protein	27	8	46.4	8.34	0.0023
1.3651	A0A0R0JL19	Uncharacterized protein	70	17	41.0	6.40	0.0001
1.3623	I1JLC8	Protein SLE2	35	3	11.5	5.60	0.0005
1.3526	I1MUR0	UBX domain-containing protein	20	8	65.5	4.73	0.0020
1.3233	I1MX00	Protein kinase domain-containing protein	9	4	75.5	6.42	0.0072
1.3199	I1J7T0	Pectinesterase (EC 3.1.1.11)	20	7	56.5	9.01	0.0118
1.3173	K7MNN1	Aminotran_1_2 domain-containing protein	12	3	52.8	7.94	0.0000
1.3010	I1J4D5	Uncharacterized protein	41	6	26.6	5.90	0.0003
1.2938	I1M3A7	Uncharacterized protein	12	3	63.8	5.73	0.0104
1.2933	I1JAV9	Pectinesterase (EC 3.1.1.11)	36	13	61.0	8.59	0.0365
1.2838	Q8LGT8	Phosphoglycerate mutase-like protein	20	4	31.8	6.34	0.0053
1.2738	I1KQB7	Uncharacterized protein	10	9	162.8	5.99	0.0021
1.2567	I1M307	Bulb-type lectin domain-containing protein	19	7	48.8	6.64	0.0059
1.2508	K7LLK0	Uncharacterized protein	19	9	88.2	5.15	0.0416
1.2499	I1L5Z2	Aldose 1-epimerase	28	6	40.7	9.32	0.0468
1.2483	I1KSE3	ALOG domain-containing protein	20	2	18.8	9.88	0.0497
1.2460	I1KG22	Uncharacterized protein	10	4	84.8	5.94	0.0010
1.2447	I1MJC0	Protein kinase domain-containing protein	3	2	139.5	5.21	0.0230
1.2397	I1MKC8	Starch synthase, chloroplastic/amyloplasti	26	10	68.1	7.06	0.0001
1.2377	A0A0R0L1W4	Glycosyltransferase	16	5	56.7	6.58	0.0211
1.2239	K7LM54	Rab-GAP TBC domain-containing protein	15	8	93.2	4.83	0.0034
1.2219	I1M3D5	Arf-GAP domain-containing protein	12	4	52.3	8.43	0.0083
1.2139	A0A0R0ESZ7	Uncharacterized protein	19	2	19.6	4.65	0.0310
1.2126	I1LKD9	Protein transport protein Sec61 subunit beta	41	4	10.9	11.65	0.0092
1.2092	C6TFE2	Uncharacterized protein	26	3	22.6	8.95	0.0106
1.2090	C6T9H6	Uncharacterized protein	26	5	42.3	7.56	0.0050
1.2052	K7MP42	Ubiquitin-like domain-containing protein	14	9	98.7	5.43	0.0123
1.2049	I1M2S7	Lactamase_B domain-containing protein	47	15	41.4	8.72	0.0012
1.2037	A0A0R0ICF9	Pectinesterase (EC 3.1.1.11)	20	2	35.4	6.34	0.0001
1.1981	I1MPF6	Uncharacterized protein	31	7	40.4	5.27	0.0002
1.1950	I1MVM6	Protein kinase domain-containing protein	20	5	40.6	8.66	0.0017
1.1816	I1K072	Uncharacterized protein	33	16	77.3	5.14	0.0038

1.1815	O23959	Ca+2-binding EF hand protein	23	4	27.0	6.44	0.0224
1.1814	Q9MAZ9	Nonclathrin coat protein zeta1-COP	46	4	20.1	4.86	0.0002
1.1751	I1N3Y0	Uncharacterized protein	15	4	46.1	7.05	0.0007
1.1747	I1KMS6	Uncharacterized protein	28	4	19.0	7.50	0.0023
1.1743	I1L437	PHB domain-containing protein	46	9	31.8	9.72	0.0345
1.1736	I1L0W9	Uncharacterized protein	18	9	85.2	8.13	0.0010
1.1726	A0A0R0FSB9	Uncharacterized protein	49	2	11.2	9.20	0.0135
1.1713	K7KSA3	Uncharacterized protein	3	2	119.9	6.46	0.0218
1.1704	I1MYR9	Uncharacterized protein	24	3	20.0	10.36	0.0001
1.1661	I1J8U0	Uncharacterized protein	16	4	45.6	6.37	0.0327
1.1659	I1MJS7	RRM domain-containing protein	2	2	97.5	6.35	0.0230
1.1652	Q2PMQ3	DNA-directed RNA polymerase subunit alph	20	4	38.7	7.56	0.0076
1.1579	C6T3U6	Ribosomal protein L19	17	2	24.8	11.44	0.0001
1.1467	A0A0R0JWL2	Uncharacterized protein	45	4	21.2	8.47	0.0069
1.1366	I1LLG6	Uncharacterized protein	5	4	179.7	6.14	0.0008
1.1320	K7MZI8	Pectinesterase (EC 3.1.1.11)	24	9	67.9	8.40	0.0182
1.1308	I1KTY1	Protein kinase domain-containing protein	4	3	138.0	5.81	0.0000
1.1304	A8IFI1	S1 ribosomal protein	24	3	23.4	10.27	0.0365
1.1245	A0A0R0GZ36	Uncharacterized protein	48	9	41.3	5.99	0.0005
1.1201	I1LSH5	Uncharacterized protein	6	2	44.7	9.64	0.0052
1.1157	I1LJ29	Uncharacterized protein	25	4	27.1	6.80	0.0158
1.1110	A0A0R0I3N7	WD_REPEATS_REGION domain-containing protein	17	5	52.6	8.94	0.0071
1.1024	I1JV82	TPR_REGION domain-containing protein	32	4	24.1	8.70	0.0228
1.1016	I1LJA4	Nop domain-containing protein	29	10	60.8	9.03	0.0045
1.1014	I1MKY3	Uncharacterized protein	40	4	12.6	6.74	0.0155
1.1014	I1LUJ6	PHB domain-containing protein	47	10	37.1	9.63	0.0245
1.1010	K7MIR3	Enolase_C domain-containing protein	67	15	38.3	6.80	0.0009
1.0996	A0A0R0E8N5	Uricase (EC 1.7.3.3) (Urate oxidase)	62	15	35.1	8.29	0.0004
1.0912	I1NAS0	EF-hand domain-containing protein	9	3	47.3	5.49	0.0078
1.0896	I1JQI7	Thioredox_DsbH domain-containing protein	12	6	90.9	6.43	0.0000
1.0852	I1NEU8	Aminotran_5 domain-containing protein	23	6	49.5	7.31	0.0095
1.0842	K7MR36	Citrate synthase (EC 2.3.3.16)	30	9	56.6	9.38	0.0120
1.0778	A0A0R0F5Y7	WD_REPEATS_REGION domain-containing protein	18	14	121.9	5.52	0.0004
1.0759	I1KW20	PHB domain-containing protein	35	7	30.5	8.35	0.0343
1.0683	I1LP93	Phospholipid scramblase	12	3	40.2	9.57	0.0015
1.0683	Q8W198	Phosphate transporter	36	12	36.3	9.04	0.0001
1.0644	K7MQ23	Uncharacterized protein	12	5	62.2	6.99	0.0009
1.0544	I1L957	Uncharacterized protein	33	11	48.8	6.42	0.0006
1.0525	A0A0A8WHK7	Expansin	25	4	27.5	8.84	0.0151
1.0519	A0A0R4J2T3	Uncharacterized protein	40	14	65.3	5.25	0.0018
1.0477	C6T9B1	cobW domain-containing protein	53	9	31.4	6.48	0.0006
1.0474	I1J9W3	J domain-containing protein	4	2	59.9	9.09	0.0393
1.0459	K7L3H1	Uncharacterized protein	11	3	47.7	9.85	0.0470
1.0439	A0A0R0HN41	Uncharacterized protein	61	4	12.0	4.46	0.0236
1.0431	Q948P5	Ferritin-4, chloroplastic	46	9	27.5	5.96	0.0120
1.0413	I1KDR0	Uncharacterized protein	64	16	57.1	5.39	0.0007
1.0367	C6TMZ4	Fe2OG dioxygenase domain-containing protein	30	5	36.0	5.38	0.0054
1.0359	K7M9T4	PUA domain-containing protein	8	2	66.2	9.25	0.0034
1.0323	I1MDA0	Uncharacterized protein	19	2	17.1	10.17	0.0079
1.0304	I1KYL4	ER membrane protein complex subunit 3	26	5	27.8	8.97	0.0010
1.0294	I1KW16	Uncharacterized protein	42	3	9.3	9.16	0.0162
1.0276	I1LLT9	Uncharacterized protein	13	3	49.2	8.78	0.0024
1.0242	C6T321	Clat_adaptor_s domain-containing protein	21	2	19.7	4.86	0.0006
1.0145	C6SWV4	Uncharacterized protein	40	5	18.8	6.32	0.0026
1.0143	I1JA23	Phospholipase D (EC 3.1.4.4)	4	3	96.0	7.75	0.0042
1.0120	I1M099	Uncharacterized protein	16	4	31.4	9.67	0.0482

1.0089	I1LS05	Uncharacterized protein	34	7	45.8	7.02	0.0010
1.0061	I1KQG0	T-complex protein 1 subunit delta	66	22	57.6	7.28	0.0145
1.0035	K7KBB0	Phi class glutathione S-transferase	39	6	24.8	6.30	0.0078
1.0023	I1J626	Uncharacterized protein	4	2	73.1	7.87	0.0276
1.0007	I1LDP2	Uncharacterized protein	16	4	27.9	8.92	0.0004
0.9995	A0A0R0EXK4	Uncharacterized protein	15	7	70.7	8.37	0.0470
0.9984	I1LPA6	Uncharacterized protein	12	4	38.8	8.31	0.0312
0.9952	I1JSN6	Usp domain-containing protein	24	2	19.9	6.33	0.0082
0.9842	I1KX25	Arf-GAP domain-containing protein	47	11	43.4	8.92	0.0014
0.9818	I1MY43	Vacuolar protein sorting-associated protein 35	15	7	90.3	5.43	0.0047
0.9800	I1LSS9	Flavodoxin-like domain-containing protein	37	5	21.8	5.57	0.0176
0.9797	I1LTF0	Uncharacterized protein	14	3	51.8	8.78	0.0031
0.9796	C6SZK3	Glutathione peroxidase	44	5	18.4	7.09	0.0001
0.9791	I1JXA0	Uncharacterized protein	54	33	90.8	5.24	0.0016
0.9784	I1NHP3	Uncharacterized protein	25	2	15.6	9.09	0.0005
0.9775	K7MVR5	Methionine aminopeptidase 2	16	3	48.2	6.48	0.0103
0.9775	I1KCD7	Uncharacterized protein	40	26	90.9	5.24	0.0133
0.9758	Q39834	Clathrin heavy chain	45	55	193.2	5.55	0.0020
0.9753	I1N5N0	Pyruvate kinase (EC 2.7.1.40)	55	19	55.2	7.56	0.0010
0.9746	I1LR42	Uncharacterized protein	5	3	93.8	4.59	0.0091
0.9659	I1LUW6	Arf-GAP domain-containing protein	7	3	52.5	8.43	0.0146
0.9635	I1LN49	Uncharacterized protein	50	8	27.0	7.06	0.0000
0.9634	I1JH91	Uncharacterized protein	5	2	92.3	6.65	0.0079
0.9590	I1KWA0	Uncharacterized protein	39	16	65.2	4.83	0.0106
0.9580	I1NC18	Glutathione synthetase	37	13	60.8	7.28	0.0053
0.9548	I1MBI9	Peroxidase (EC 1.11.1.7)	26	7	40.0	9.54	0.0259
0.9516	A0A0R0EEE4	HMA domain-containing protein	32	3	13.6	7.72	0.0399
0.9515	I1K0C8	UBX domain-containing protein	26	9	65.7	4.73	0.0047
0.9493	K7LQ69	Poly [ADP-ribose] polymerase	38	20	91.1	5.43	0.0018
0.9469	A0A368UH41	TOG domain-containing protein	14	9	118.8	6.76	0.0031
0.9433	I1JLY3	Peptidylprolyl isomerase (EC 5.2.1.8)	17	7	69.9	5.53	0.0270
0.9389	I1JJC9	Uncharacterized protein	5	2	61.6	8.07	0.0190
0.9384	A0A0R0G0T0	PfkB domain-containing protein	33	9	37.9	5.63	0.0012
0.9311	I1LPC3	Uncharacterized protein	28	21	134.7	6.58	0.0087
0.9294	I1JUR9	Uncharacterized protein	8	2	37.8	8.78	0.0057
0.9207	I1JYF7	PHB domain-containing protein	43	12	43.9	9.11	0.0147
0.9161	I1KLY3	Abhydrolase_3 domain-containing protein	11	2	35.0	5.76	0.0235
0.9048	Q84ZV2	Uncharacterized protein	33	2	11.3	9.13	0.0025
0.9040	I1LNJ4	SAM_MT_RSMB_NOP domain-containing protein	3	2	91.5	6.10	0.0432
0.9000	Q39801	51 kDa seed maturation protein	25	10	51.0	7.11	0.0058
0.8976	K7LRE0	Uncharacterized protein	24	4	10.3	5.21	0.0041
0.8967	B6CAM2	Matrix metalloproteinase	17	5	44.0	5.86	0.0152
0.8937	I1MK76	Pectinesterase (EC 3.1.1.11)	20	7	54.7	7.75	0.0128
0.8882	I1LGL4	Uncharacterized protein	26	6	45.1	7.20	0.0057
0.8868	I1K4P5	Uncharacterized protein	10	2	40.2	5.45	0.0063
0.8790	I1MBD1	Clathrin heavy chain	37	43	192.7	5.36	0.0429
0.8765	A0A0R0JRB0	L-lactate dehydrogenase (EC 1.1.1.27)	51	10	37.8	7.02	0.0005
0.8744	K7LRS0	Bac_surface_Ag domain-containing protein	9	3	59.1	5.52	0.0199
0.8736	I1M0K3	Cysteine proteinase inhibitor	50	9	27.7	7.08	0.0008
0.8728	I1LGI3	RanBD1 domain-containing protein	8	2	39.1	7.18	0.0139
0.8705	I1KJ35	Uncharacterized protein	27	11	58.7	8.97	0.0326
0.8699	C6SWI0	Thioredoxin domain-containing protein	16	3	26.1	5.29	0.0009
0.8659	I1L0S5	Uncharacterized protein	40	5	19.0	6.93	0.0033
0.8635	I1MMG2	Uncharacterized protein	20	5	40.8	5.92	0.0323
0.8632	C6SZ12	U6 snRNA-associated Sm-like protein LSm1	27	2	14.5	5.07	0.0426
0.8632	Q39871	Late embryongenesis abundant protein	25	9	50.6	6.67	0.0112

0.8606	C6SZ11	Uncharacterized protein	46	7	27.0	6.92	0.0297
0.8593	I1J7C8	Peptidase_M24 domain-containing protein	44	11	42.9	6.76	0.0001
0.8585	I1LG74	NAD(P)-bd_dom domain-containing protein	16	5	42.7	7.28	0.0190
0.8585	C6T294	zf-Tim10_DDP domain-containing protein	51	4	10.9	7.21	0.0331
0.8578	I1MMH5	Pectin acetylesterase	50	15	45.8	8.32	0.0299
0.8575	K7LJP8	Uncharacterized protein	11	9	182.3	6.24	0.0009
0.8563	C6SWV3	Uncharacterized protein	66	11	27.6	6.10	0.0001
0.8551	A1YZE0	Starch synthase, chloroplastic/amyloplastic	24	9	68.0	7.06	0.0030
0.8535	C6T1E1	t-SNARE coiled-coil homology protein	44	4	14.4	5.90	0.0001
0.8512	I1KIT0	Uncharacterized protein	12	2	53.9	6.46	0.0337
0.8488	I1M222	Uncharacterized protein	29	6	25.6	5.68	0.0292
0.8449	A0A0R0IJV1	Uncharacterized protein	26	7	36.9	8.27	0.0445
0.8413	I1LBM1	Uncharacterized protein	10	3	57.9	9.17	0.0025
0.8406	I1M8R6	WPP domain-containing protein	22	2	13.1	4.60	0.0459
0.8395	B1ACD0	Kunitz trypsin inhibitor p20-1-like protein	25	3	22.7	5.58	0.0194
0.8376	C6T2S4	Uncharacterized protein	55	7	22.2	7.47	0.0002
0.8369	K7MQM3	C2 domain-containing protein	26	8	46.5	4.67	0.0055
0.8367	I1JUR5	S5 DRBM domain-containing protein	14	4	57.1	6.70	0.0069
0.8365	K7KZN7	Uncharacterized protein	16	13	129.9	7.96	0.0020
0.8353	Q39873	Lea protein	22	8	49.4	7.52	0.0160
0.8340	I1KEY3	J domain-containing protein	19	4	38.4	9.04	0.0154
0.8292	I1KV60	RuvB-like helicase (EC 3.6.4.12)	22	6	50.2	5.82	0.0066
0.8289	C6T9Z5	Formate dehydrogenase, mitochondrial	58	15	42.8	6.77	0.0031
0.8283	I1LQR8	Uncharacterized protein	34	5	26.2	7.85	0.0079
0.8273	I1K1V3	Uncharacterized protein	16	2	17.3	5.30	0.0240
0.8251	I1LF85	RING-type domain-containing protein	14	2	31.8	4.75	0.0381
0.8247	I1JAU2	Uncharacterized protein	25	9	62.0	5.68	0.0051
0.8221	I1MU56	Uncharacterized protein	24	11	82.2	6.25	0.0058
0.8172	I1M860	TOG domain-containing protein	16	4	37.8	5.31	0.0251
0.8151	I1N610	Uncharacterized protein	16	4	35.5	6.05	0.0005
0.8144	C6T199	zf-Tim10_DDP domain-containing protein	39	2	9.7	6.01	0.0079
0.8136	C6SZR4	Uncharacterized protein	35	6	28.2	6.42	0.0012
0.8128	I1L7W8	Phi class glutathione S-transferase	34	4	24.7	5.44	0.0160
0.8115	K7LPS4	NOT2_3_5 domain-containing protein	10	3	70.0	7.64	0.0447
0.8068	I1MYV2	SAP domain-containing protein	14	6	78.0	4.74	0.0015
0.8050	I1MUN3	Nop domain-containing protein	33	13	61.1	8.43	0.0040
0.8032	K7MXK9	Hydrolase_4 domain-containing protein	13	4	77.3	7.94	0.0309
0.8007	C6T244	Uncharacterized protein	30	2	14.0	5.17	0.0223
0.7994	K7K2H1	Nsp1_C domain-containing protein	5	2	74.1	4.77	0.0034
0.7988	C6T1X7	Uncharacterized protein	38	2	12.5	9.92	0.0123
0.7973	I1K811	Uncharacterized protein	13	4	51.3	6.52	0.0014
0.7964	I1MAX9	SAICAR_synt domain-containing protein	17	4	46.0	6.28	0.0105
0.7945	C6SW70	Uncharacterized protein	42	11	28.2	9.88	0.0225
0.7939	K7K780	Uncharacterized protein	11	9	132.9	5.31	0.0133
0.7930	I1M7N8	Uncharacterized protein	20	5	32.7	4.96	0.0031
0.7923	I1M984	AA_TRNA_LIGASE_II domain-containing protein	45	19	60.2	6.10	0.0034
0.7920	I1JS10	Uncharacterized protein	86	2	6.8	8.92	0.0002
0.7917	C6T078	Uncharacterized protein	18	2	14.3	10.83	0.0059
0.7910	I1JZT5	Plasma membrane ATPase (EC 7.1.2.1)	36	25	105.0	6.73	0.0084
0.7887	A0A0R4J337	Expansin	21	4	27.6	8.27	0.0452
0.7876	K7KWK9	Uncharacterized protein	7	9	212.6	5.80	0.0018
0.7838	C6T588	Bet_v_1 domain-containing protein	61	7	16.8	4.88	0.0306
0.7819	I1JV20	Uncharacterized protein	6	2	52.6	6.10	0.0078
0.7774	I1JVB1	Uncharacterized protein	38	2	10.3	10.05	0.0016
0.7722	K7MBG3	Sucrose synthase (EC 2.4.1.13)	27	17	91.5	6.24	0.0035
0.7696	C6SXW1	Peptide-methionine (R)-S-oxide reductase	41	3	15.1	6.74	0.0164

0.7689	I1NG02	Thioredoxin domain-containing protein	31	4	24.4	5.57	0.0190
0.7687	C6TDW7	Uncharacterized protein	15	4	25.5	8.16	0.0001
0.7666	C6T8S2	Uncharacterized protein	29	5	32.5	5.27	0.0014
0.7641	I1JU82	Uncharacterized protein	13	9	109.2	7.44	0.0015
0.7621	K7LK00	Uncharacterized protein	4	2	83.4	6.04	0.0126
0.7617	K7KPN5	PPR_long domain-containing protein	13	4	50.9	7.88	0.0014
0.7582	I1JMK6	Uncharacterized protein	16	3	52.2	5.81	0.0032
0.7574	C6T8Y6	AB hydrolase-1 domain-containing protein	29	5	35.4	6.46	0.0077
0.7560	C6T9B7	Uncharacterized protein	21	5	31.6	7.24	0.0029
0.7471	A0A0R0I177	Pyr_redox_2 domain-containing protein	55	16	46.8	5.91	0.0004
0.7471	I1KYU6	Alpha-1,4 glucan phosphorylase (EC 2.4.1.1)	33	17	95.8	6.43	0.0136
0.7467	I1JDD2	Uncharacterized protein	29	7	48.8	7.21	0.0193
0.7439	I1KD09	ATP synthase subunit gamma	41	11	35.4	9.06	0.0342
0.7421	K7KUE2	Uncharacterized protein	20	7	83.7	4.98	0.0241
0.7417	I1N628	Uncharacterized protein	53	9	29.7	9.07	0.0388
0.7409	I1JQD8	UBA domain-containing protein	14	3	45.2	7.21	0.0016
0.7407	C6SYQ5	GLTP domain-containing protein	32	5	22.7	7.42	0.0040
0.7394	C6T1V2	SHSP domain-containing protein	46	4	17.7	6.81	0.0464
0.7371	I1JDM8	W2 domain-containing protein	17	6	48.7	6.64	0.0128
0.7367	K7L2B3	Glyco_transf_20 domain-containing protein	19	10	96.9	5.82	0.0314
0.7364	B3TDK6	Lipoxygenase (EC 1.13.11.-)	73	45	96.7	6.58	0.0066
0.7339	C6TB70	Thioredoxin domain-containing protein	56	7	24.4	6.92	0.0034
0.7336	C6TCX5	Peptidase_M24 domain-containing protein	35	10	43.5	6.70	0.0222
0.7332	Q9SWB6	Probable bifunctional TENA-E protein	38	10	26.0	5.35	0.0000
0.7332	K7MT91	Aldo_ket_red domain-containing protein	69	12	32.3	6.73	0.0236
0.7316	C6T3P3	Uncharacterized protein	19	4	24.3	8.29	0.0330
0.7310	A0A0R4J3P8	Peptidase_M24 domain-containing protein	42	11	43.3	6.86	0.0005
0.7310	C6TK48	Uncharacterized protein	7	2	44.9	5.57	0.0015
0.7298	I1MTB5	B5 domain-containing protein	36	14	67.8	5.72	0.0296
0.7291	I1LA46	Uncharacterized protein	25	5	30.6	6.68	0.0002
0.7243	C6TJT0	Uncharacterized protein	51	11	39.9	8.87	0.0079
0.7237	A0A0R0GXL3	MLO-like protein	12	5	61.3	10.45	0.0027
0.7234	A0A0R0KW93	Uncharacterized protein	5	2	65.0	5.05	0.0326
0.7191	B6ZJZ9	Peroxisomal adenine nucleotide carrier 1 (GmPNC1)	15	3	34.7	9.69	0.0081
0.7162	C6TMK3	Cysteine proteinase inhibitor	53	8	27.6	7.80	0.0373
0.7127	K7KWZ7	PPM-type phosphatase domain-containing protein	28	5	30.8	6.30	0.0005
0.7102	K7M6T0	Macro domain-containing protein	28	3	25.3	8.16	0.0207
0.7093	I1LMQ7	Uncharacterized protein	3	2	89.5	7.25	0.0035
0.7084	I1LES5	PB1 domain-containing protein	25	8	76.5	6.18	0.0066
0.7077	I1MTZ8	Tr-type G domain-containing protein	19	12	110.0	5.17	0.0200
0.7069	C6SVI1	V-type proton ATPase subunit F	55	5	14.5	6.57	0.0022
0.7065	A0A0R0LA35	Epimerase domain-containing protein	39	7	35.2	6.52	0.0066
0.7051	K7LD23	Uncharacterized protein	15	7	66.2	7.53	0.0385
0.7040	C6TFT8	Annexin	42	11	35.6	6.46	0.0293
0.6991	I1LCQ1	Pyruvate kinase (EC 2.7.1.40)	36	14	57.8	7.36	0.0003
0.6971	A0A0R4J3X4	SHSP domain-containing protein	31	4	25.6	7.49	0.0491
0.6967	I1JE17	Glycosyltransferase	50	13	53.1	5.86	0.0060
0.6960	I1KJZ4	Uncharacterized protein	15	4	52.5	5.25	0.0092
0.6955	I1J700	Aldo_ket_red domain-containing protein	28	8	35.0	6.73	0.0236
0.6950	I1N5M3	Elongation factor Ts, mitochondrial	7	2	38.3	8.24	0.0010
0.6908	C6T770	RING-type domain-containing protein	21	4	37.2	8.12	0.0021
0.6871	C6TER4	NmrA domain-containing protein	33	8	34.9	6.81	0.0309
0.6867	I1L7B5	Uncharacterized protein	15	7	67.9	8.44	0.0200
0.6847	C6TNZ4	Ferredoxin	49	3	16.3	5.10	0.0466
0.6841	C6SZZ1	Translocon-associated protein subunit beta	26	3	20.9	9.36	0.0018
0.6839	A0A368UHY2	Uncharacterized protein	10	33	559.5	6.16	0.0024
0.6823	I1LAX4	Uncharacterized protein	35	14	82.9	7.43	0.0125

0.6798	I1J7G5	Uncharacterized protein	47	5	18.6	5.02	0.0002
0.6771	C6T2R8	Uncharacterized protein	25	5	25.0	5.57	0.0047
0.6766	I1MHF8	Uncharacterized protein	15	8	99.4	5.29	0.0219
0.6762	I1KBB6	4-alpha-glucanotransferase	32	10	63.9	5.96	0.0142
0.6761	I1M596	Lipoxygenase (EC 1.13.11.-)	69	39	97.2	6.67	0.0144
0.6739	Q9LLQ6	Seed maturation protein PM34	71	17	31.7	7.08	0.0002
0.6696	I1N0Q9	Uncharacterized protein	7	3	85.8	5.83	0.0189
0.6674	I1MKT8	Glycosyltransferase	29	8	54.4	6.84	0.0087
0.6656	K7MGK0	AP-4 complex subunit epsilon	22	14	107.7	6.11	0.0006
0.6646	I1KTV3	Ribosomal_L18e/L15P domain-containing protein	12	3	31.0	11.09	0.0263
0.6637	I1L1F3	Uncharacterized protein	38	7	28.3	9.83	0.0321
0.6636	I1LP68	Bet_v_1 domain-containing protein	40	4	17.7	4.81	0.0008
0.6626	C6T374	VOC domain-containing protein	61	4	15.4	5.69	0.0006
0.6607	I1NAA0	Uncharacterized protein	26	5	33.8	7.46	0.0038
0.6589	I1KBB9	OBG-type G domain-containing protein	21	5	42.6	8.65	0.0175
0.6581	K7MJA4	Uncharacterized protein	1	2	186.8	7.14	0.0304
0.6580	I1KYG2	C2 domain-containing protein	26	8	46.6	4.70	0.0081
0.6577	I1LNT7	Uncharacterized protein	6	4	83.7	6.01	0.0136
0.6574	I1MU70	Uncharacterized protein	17	7	80.0	6.09	0.0193
0.6568	C6TD56	Glyceraldehyde-3-phosphate dehydrogenase	69	17	37.0	7.47	0.0232
0.6541	K7MH27	Uncharacterized protein	37	6	26.5	9.20	0.0386
0.6519	I1NI24	ETF domain-containing protein	38	5	27.7	7.83	0.0297
0.6517	C6SXN1	Uncharacterized protein	15	2	19.1	8.15	0.0290
0.6507	I1MW74	PCI domain-containing protein	27	8	47.0	6.04	0.0444
0.6494	I1KLK0	Lipoxygenase (EC 1.13.11.-)	4	2	103.0	7.31	0.0041
0.6477	C6T0R5	Ribosomal_L18e/L15P domain-containing protein	20	3	21.0	11.18	0.0038
0.6413	A0A0R0F1L5	Uncharacterized protein	25	5	28.3	7.05	0.0282
0.6408	I1L0E5	Ubiquitin-like domain-containing protein	44	7	17.7	9.74	0.0039
0.6366	C6SY72	SWIB domain-containing protein	17	2	14.4	10.23	0.0131
0.6354	A0A0R4J3C5	Uncharacterized protein	21	7	51.4	7.64	0.0175
0.6315	I1MH79	Translation machinery-associated protein 22	40	5	21.0	6.73	0.0088
0.6297	I1K7Z3	Uncharacterized protein	12	2	29.0	9.04	0.0406
0.6297	I1K4M7	Uncharacterized protein	38	17	70.8	5.21	0.0038
0.6263	I1LEY1	Uncharacterized protein	54	18	65.4	5.03	0.0373
0.6243	I1MRJ8	Uncharacterized protein	14	8	78.9	8.68	0.0126
0.6228	C6TBR0	Uncharacterized protein	11	2	29.4	6.52	0.0046
0.6218	K7KS35	Theta class glutathione S-transferase	19	3	28.4	9.64	0.0228
0.6213	I1N6Y7	Uncharacterized protein	10	5	85.9	7.14	0.0119
0.6175	I1MT10	Uncharacterized protein	54	26	70.8	5.39	0.0002
0.6141	A0A0R0IQ81	Glutaredoxin	35	4	17.9	7.37	0.0072
0.6137	I1J495	Uncharacterized protein	10	5	87.5	6.90	0.0156
0.6114	I1JB78	Uncharacterized protein	13	3	48.9	5.82	0.0005
0.6092	I1JRM7	Uncharacterized protein	53	10	35.7	4.73	0.0446
0.6089	I1J575	Uncharacterized protein	31	5	28.8	8.51	0.0060
0.6084	C6SYZ8	Uncharacterized protein	23	5	23.6	10.32	0.0065
0.6062	B0M195	Peroxisomal short-chain dehydrogenase/reductase	29	4	26.3	8.69	0.0147
0.6059	A0A0R0J965	Uncharacterized protein	50	11	34.4	6.90	0.0066
0.6059	I1N4A7	Uncharacterized protein	54	9	29.8	8.65	0.0260
0.6043	C6T3E5	MPN domain-containing protein	39	4	22.9	5.78	0.0053
0.6035	I1KB73	tRNA-binding domain-containing protein	35	8	42.3	6.29	0.0002
0.6033	I1MRK4	Dihydrolipoamide acetyltransferase component of pyruvate dehydrogenase complex (EC 2.3.1.-)	28	9	56.2	7.02	0.0083
0.6029	I1LZK9	Uncharacterized protein	17	5	65.4	6.87	0.0225
0.6015	I1KNN0	ATP-dependent 6-phosphofructokinase	41	11	51.1	6.71	0.0014
0.6009	I1MZJ8	Uncharacterized protein	15	4	41.1	5.62	0.0065
0.6007	C6T763	PCI domain-containing protein	58	11	30.8	5.15	0.0013

0.5997	I1KI20	SufE domain-containing protein	14	3	42.7	5.78	0.0038
0.5965	I1N1M0	ATP-dependent 6-phosphofructokinase	13	4	58.4	7.59	0.0466
0.5962	I1N4I4	Uncharacterized protein	6	2	50.2	8.09	0.0421
0.5941	A0A0R0EHQ5	Uncharacterized protein	1	2	239.4	6.83	0.0409
0.5934	A0A0R0JJK8	Uncharacterized protein	15	7	69.4	7.27	0.0033
0.5928	A0A368UIG3	Dolichyl-diphosphooligosaccharide--protein glycosyltransferase subunit 1	27	9	52.3	7.80	0.0078
0.5923	I1NBE6	Uncharacterized protein	23	12	82.1	7.37	0.0003
0.5907	A0A0R0JW56	Pyruvate kinase (EC 2.7.1.40)	55	20	55.3	7.56	0.0003
0.5903	I1KAV2	Uncharacterized protein	10	3	38.8	4.64	0.0190
0.5899	C6TJP9	WD_REPEATS_REGION domain-containing protein	8	2	38.2	7.06	0.0215
0.5893	I1NIN5	Uncharacterized protein	9	6	108.4	5.39	0.0187
0.5871	K7KP23	Uncharacterized protein	5	2	60.5	9.00	0.0247
-0.5853	I1JU62	Transmembrane 9 superfamily member	20	9	73.1	7.62	0.0072
-0.5853	A0A0R0J895	Terpene cyclase/mutase family member (EC 5.4.99.-)	4	2	87.5	6.76	0.0469
-0.5856	I1KQ30	Aldedh domain-containing protein	52	19	54.8	6.04	0.0082
-0.5857	C6TDP2	Uncharacterized protein	30	8	30.1	7.93	0.0024
-0.5862	I1MR46	Uncharacterized protein	23	8	62.0	9.41	0.0188
-0.5882	I1LBH6	Uncharacterized protein	58	18	50.5	5.00	0.0060
-0.5884	A0A0R4J301	Endoglucanase (EC 3.2.1.4)	9	4	57.7	6.09	0.0046
-0.5893	I1MVC1	Uncharacterized protein	41	15	69.4	8.35	0.0121
-0.5904	I1JRY5	Uncharacterized protein	20	5	37.4	9.55	0.0134
-0.5909	I1N1U5	Glycosyltransferase (EC 2.4.1.-)	29	9	53.3	5.60	0.0120
-0.5952	I1K1I6	Isocitrate dehydrogenase [NADP] (EC 1.1.1.42)	30	13	53.3	8.12	0.0132
-0.5952	I1LCX7	RING-type E3 ubiquitin transferase (EC 2.3.2.27)	4	2	68.7	6.05	0.0018
-0.5969	I1MJH5	Uncharacterized protein	30	8	46.7	5.59	0.0109
-0.5980	I1M6K2	C2 domain-containing protein	15	4	46.1	4.68	0.0102
-0.5989	I1M989	Transmembrane 9 superfamily member	19	10	73.1	7.96	0.0090
-0.5989	I1MGG7	Uncharacterized protein	27	9	60.1	7.36	0.0138
-0.5995	C6TH16	Reticulon-like protein	15	2	28.6	7.65	0.0216
-0.6019	I1KPM9	TPR_REGION domain-containing protein	17	8	63.2	5.41	0.0168
-0.6032	I1L5G4	Amidophosphoribosyltransferase	27	11	62.0	6.84	0.0011
-0.6043	I1LY51	Oxidored_FMN domain-containing protein	72	16	44.1	8.15	0.0143
-0.6065	A0A0R4J2N9	Fe2OG dioxygenase domain-containing protein	31	6	40.6	5.82	0.0017
-0.6068	K7KSH9	MFS domain-containing protein	12	5	78.8	5.71	0.0105
-0.6075	A0A0R0FJI5	Uncharacterized protein	27	20	152.8	6.54	0.0087
-0.6081	I1LJJ4	Uncharacterized protein	57	17	53.5	6.87	0.0157
-0.6092	A6BM07	Isoflavone 7-O-glucosyltransferase 1	11	3	52.0	5.34	0.0480
-0.6096	I1LIV5	FAD-binding PCMH-type domain-containing protein	47	19	66.0	8.21	0.0015
-0.6113	I1LYG4	t-SNARE coiled-coil homology protein	19	4	30.3	6.58	0.0022
-0.6141	I1MR41	Aminomethyltransferase	40	10	44.3	8.15	0.0035
-0.6159	A0A0R4J5C5	Uncharacterized protein	63	8	27.6	7.30	0.0235
-0.6163	Q9FQE7	Glutathione S-transferase	49	7	25.5	6.24	0.0358
-0.6164	I1LXH8	Uncharacterized protein	18	5	50.8	6.73	0.0022
-0.6182	A0A0R0FLF0	Xyloglucan endotransglucosylase/hydrolase	25	8	40.4	9.25	0.0078
-0.6193	A0A0R0HFK7	Exostosin domain-containing protein	5	2	59.4	8.84	0.0336
-0.6200	I1M8Q5	Uncharacterized protein	28	10	54.4	6.02	0.0080
-0.6239	A0A0R4J3B7	Lipase_GDSL domain-containing protein	23	4	39.7	4.84	0.0329
-0.6258	I1M017	PPIase cyclophilin-type domain-containing protein	19	4	48.7	5.20	0.0034
-0.6262	I1JN48	DNA gyrase subunit B (EC 5.6.2.3)	7	3	81.1	8.38	0.0178
-0.6264	I1MEW6	SOR_SNZ domain-containing protein	20	6	33.2	6.11	0.0091
-0.6266	K7MLF2	AAA domain-containing protein	12	8	132.6	6.61	0.0117
-0.6295	I1MD33	LsmAD domain-containing protein	3	2	69.3	5.71	0.0231
-0.6317	I1LU49	PKS_ER domain-containing protein	41	8	38.2	6.00	0.0136
-0.6334	I1KPI6	NAB domain-containing protein	2	2	162.5	5.01	0.0427
-0.6359	Q6YGT9	Purple acid phosphatase (EC 3.1.3.2)	53	15	59.5	8.10	0.0004
-0.6359	I1M658	Transmembrane 9 superfamily member	15	7	75.1	6.96	0.0022

-0.6389	A0A0R0IG82	Aldedh domain-containing protein	18	6	56.5	6.42	0.0081
-0.6424	A0A0R0GAK0	Uncharacterized protein	48	10	30.6	8.40	0.0161
-0.6428	I1JM18	Transket_pyr domain-containing protein	41	7	39.0	6.54	0.0118
-0.6438	I1KP02	Uncharacterized protein	27	11	65.3	5.05	0.0083
-0.6468	C6THB9	Uncharacterized protein	43	12	45.6	6.35	0.0076
-0.6472	I1L327	DUF862 domain-containing protein	19	3	27.2	7.34	0.0161
-0.6505	C6TK68	TPT domain-containing protein	11	3	36.2	9.29	0.0055
-0.6533	I1N4U1	6-phosphogluconate dehydrogenase, decarboxylating	59	20	53.6	5.90	0.0035
-0.6546	F6KBT5	Allene oxide cyclase 5 (EC 5.3.99.6)	23	5	28.4	8.63	0.0460
-0.6569	Q8S5C1	4-coumarate:CoA ligase isoenzyme 2 (EC 6.2.1.12)	27	11	60.1	6.74	0.0166
-0.6576	I1JIC8	PRA1 family protein	26	3	23.1	9.82	0.0112
-0.6585	I1MDX7	Uncharacterized protein	14	3	31.9	8.15	0.0331
-0.6608	C6TK33	Uncharacterized protein	41	10	32.5	6.70	0.0103
-0.6681	K7MNJ0	GH10 domain-containing protein	11	8	103.3	6.92	0.0441
-0.6697	A0A0R0LJH9	Protein transport protein sec16	4	4	152.0	5.12	0.0252
-0.6712	A0A0R0JMI0	Glutamine amidotransferase type-2	16	20	239.4	6.57	0.0081
-0.6718	I1JUM2	Plasma membrane ATPase (EC 7.1.2.1)	28	19	104.4	6.93	0.0383
-0.6725	K7LZA7	SHSP domain-containing protein	11	2	48.1	5.36	0.0262
-0.6726	C6T218	Cytochrome b5 heme-binding protein	36	2	13.6	5.11	0.0094
-0.6803	C6SVR0	Plastocyanin	39	3	16.8	5.20	0.0231
-0.6813	I1LJG0	Glycosyltransferase (EC 2.4.2.-)	22	6	49.4	8.78	0.0079
-0.6817	I1MC85	Uncharacterized protein	32	10	45.8	5.82	0.0202
-0.6826	A0A0R0HNL5	NAB domain-containing protein	2	4	251.2	4.97	0.0007
-0.6843	I1KM16	Uncharacterized protein	64	13	28.1	5.68	0.0002
-0.6845	A0A0R0GYY6	Abhydrolase_3 domain-containing protein	35	7	30.5	5.29	0.0052
-0.6873	A0A0R0H975	Apocytochr_F_N domain-containing protein	34	7	35.3	8.82	0.0163
-0.6903	K7MU41	Uncharacterized protein	26	10	66.9	8.53	0.0004
-0.6913	I1MM41	Uncharacterized protein	9	3	58.3	6.00	0.0194
-0.6924	I1KUF1	Fn3_like domain-containing protein	15	7	83.8	6.02	0.0026
-0.6931	C6TCI6	Glutamate decarboxylase (EC 4.1.1.15)	45	15	56.2	5.81	0.0048
-0.6931	A0A368UJK6	Uncharacterized protein	12	7	125.3	5.26	0.0005
-0.6938	I1MR83	DUF3700 domain-containing protein	32	5	25.5	5.60	0.0219
-0.6952	I1K478	YTH domain-containing protein	13	7	77.1	6.62	0.0036
-0.6956	I1J8X9	Flavodoxin-like domain-containing protein	25	4	28.0	7.20	0.0102
-0.6989	C6T2H1	Flavodoxin-like domain-containing protein	57	8	21.7	6.95	0.0470
-0.7041	I1L2I7	Serine/threonine-protein phosphatase (EC 3.1.3.16)	33	7	35.8	5.41	0.0273
-0.7059	I1LUM7	ALOG domain-containing protein	37	4	19.6	9.09	0.0016
-0.7087	I1K2B5	Uncharacterized protein	20	9	79.6	6.60	0.0011
-0.7130	I1K642	Uncharacterized protein	13	5	53.6	6.68	0.0150
-0.7138	I1LU10	Uncharacterized protein	46	13	46.3	6.30	0.0020
-0.7147	I1LN30	Adenosylhomocysteinase (EC 3.3.1.1)	61	20	53.3	6.23	0.0035
-0.7159	I1N8A2	Uncharacterized protein	11	5	69.7	6.90	0.0005
-0.7252	I1KHB7	Germin-like protein	46	3	23.8	8.46	0.0003
-0.7281	A0A0R0JQA9	X8 domain-containing protein	19	2	21.5	7.24	0.0031
-0.7290	I1LJH2	Uncharacterized protein	32	9	52.0	5.36	0.0025
-0.7320	I1L1Q3	Uncharacterized protein	12	2	45.1	7.20	0.0178
-0.7322	A0A0R0EQE5	Uncharacterized protein	33	16	70.3	6.04	0.0190
-0.7352	I1K512	RING-type domain-containing protein	10	2	47.3	6.09	0.0048
-0.7373	I1KRD0	Uncharacterized protein	12	2	47.8	6.30	0.0248
-0.7421	A0A0R0JPL6	Peptidase A1 domain-containing protein	44	10	50.0	8.24	0.0171
-0.7426	I1KIA1	Post-GPI attachment to proteins factor 3	5	2	39.9	7.27	0.0470
-0.7452	E2FKH8	Sieve element occlusion by forisomes 1	7	3	75.2	6.64	0.0315
-0.7472	B1NC19	Putative cysteine protease	14	3	39.0	7.30	0.0019
-0.7480	I1MQP2	Cellulase domain-containing protein	7	3	56.2	5.62	0.0158
-0.7485	I1KIJ3	Uncharacterized protein	3	2	117.3	7.12	0.0397
-0.7488	I1NHB5	Dihydrolipoamide acetyltransferase component of pyruvate dehydrogenase complex	32	9	48.4	8.63	0.0487

-0.7490	I1L2C2	Transmembrane 9 superfamily member	21	10	74.9	6.18	0.0048
-0.7506	A0A0R4J5R0	Fe2OG dioxygenase domain-containing protein	19	4	37.0	5.88	0.0172
-0.7560	G0T437	Purple acid phosphatase (EC 3.1.3.2)	17	7	74.1	6.35	0.0055
-0.7574	I1J940	RPW8 domain-containing protein	6	3	94.5	6.51	0.0027
-0.7584	I1J4T0	t-SNARE coiled-coil homology protein	12	2	30.1	6.10	0.0026
-0.7584	I1MGZ2	Plasma membrane ATPase (EC 7.1.2.1)	40	29	105.0	6.81	0.0052
-0.7593	I1JE07	Uncharacterized protein	45	12	36.9	7.05	0.0195
-0.7594	C6TMK6	AAI domain-containing protein	40	3	13.3	8.05	0.0380
-0.7598	I1NBJ8	Pectin acetylesterase (EC 3.1.1.-)	35	6	45.2	7.20	0.0097
-0.7643	C6T225	Uncharacterized protein (Fragment)	24	4	22.2	5.00	0.0003
-0.7643	I1KSB7	X8 domain-containing protein	13	4	53.0	5.16	0.0326
-0.7672	C6T2V7	Phytocyanin domain-containing protein	18	2	26.1	4.87	0.0168
-0.7698	I1KYC6	Peptidase A1 domain-containing protein	14	4	51.9	7.84	0.0066
-0.7700	A0A0R4J5M9	Uncharacterized protein	13	3	38.8	7.59	0.0003
-0.7705	I1M1I9	Uncharacterized protein	5	3	82.0	9.28	0.0190
-0.7708	I1KBK8	Uncharacterized protein	23	13	91.0	5.25	0.0008
-0.7753	I1MT77	Uncharacterized protein	29	8	50.1	5.67	0.0227
-0.7762	I1K775	X8 domain-containing protein	12	3	48.3	5.00	0.0010
-0.7770	I1KRP1	Transmembrane 9 superfamily member	20	11	69.2	7.47	0.0002
-0.7792	C5HU39	AIR12	20	3	25.6	7.50	0.0176
-0.7796	I1JVD1	Uncharacterized protein	6	3	78.7	6.73	0.0325
-0.7803	I1MZ96	Uncharacterized protein	9	4	75.4	5.72	0.0003
-0.7811	I1LTS0	Arogenate dehydratase (EC 4.2.1.91)	17	5	46.2	7.84	0.0272
-0.7874	I1JL58	PRA1 family protein	11	2	24.2	8.19	0.0001
-0.7874	C6SYW1	Bet_v_1 domain-containing protein	44	6	17.0	5.12	0.0049
-0.7877	I1L7Z5	Peroxidase (EC 1.11.1.7)	18	4	35.1	6.30	0.0256
-0.7895	I1J5A5	Aldedh domain-containing protein	50	16	58.4	6.84	0.0006
-0.7909	I1K7Q8	NmrA domain-containing protein	60	12	34.2	6.06	0.0189
-0.7930	P13708	Sucrose synthase	75	46	92.2	6.48	0.0160
-0.7935	K7L6B6	NAD(P)-bd_dom domain-containing protein	9	3	45.7	8.98	0.0071
-0.7942	C6TH50	Xyloglucan endotransglucosylase/hydrolase	22	5	35.0	5.01	0.0004
-0.7957	I1LWZ5	PMR5N domain-containing protein	7	2	50.4	6.84	0.0387
-0.8009	C6TA28	Nucleoside diphosphate kinase (EC 2.7.4.6)	15	2	25.2	9.03	0.0002
-0.8039	C6SX29	ACT domain-containing protein	22	2	16.6	4.70	0.0107
-0.8054	Q6RUF9	Proline dehydrogenase (EC 1.5.5.2)	9	3	54.4	8.13	0.0014
-0.8117	I1JJA3	Uncharacterized protein	8	3	59.5	6.80	0.0058
-0.8130	I1LZN1	Transmembrane 9 superfamily member	17	8	77.5	6.96	0.0197
-0.8163	I1MV80	Cupin_2 domain-containing protein	51	7	33.0	6.61	0.0218
-0.8211	I1NAB2	Uncharacterized protein	9	8	146.2	7.62	0.0359
-0.8217	A0A0R0JYE8	Uncharacterized protein	3	2	101.0	8.53	0.0058
-0.8224	I1JIW1	Amidohydro-rel domain-containing protein	19	5	57.8	6.54	0.0007
-0.8243	A0A0R0KVX2	Fe2OG dioxygenase domain-containing protein	15	3	39.7	6.84	0.0495
-0.8281	C6THF2	Uncharacterized protein	14	2	31.5	5.36	0.0015
-0.8284	A0A0R0GFB2	Uncharacterized protein	54	18	57.5	8.87	0.0349
-0.8332	A0A0R4J4S6	NADPH-protochlorophyllide oxidoreductase	52	13	43.2	8.78	0.0091
-0.8379	I1K7G0	Methyltransferase (EC 2.1.1.-)	34	10	40.4	6.74	0.0061
-0.8399	C6TJF9	Uncharacterized protein	55	8	24.0	6.58	0.0141
-0.8401	I1JHV7	Uncharacterized protein	10	2	29.7	7.64	0.0025
-0.8448	C6T1F3	Uncharacterized protein	26	2	12.1	9.61	0.0154
-0.8471	I1KUQ6	Lipoxygenase (EC 1.13.11.-)	26	15	97.1	7.40	0.0152
-0.8524	K7M429	Uncharacterized protein	30	10	45.6	6.28	0.0001
-0.8547	I1J4P0	TFIIS N-terminal domain-containing protein	5	2	57.6	5.38	0.0387
-0.8552	A0A0R4J508	Expansin	11	2	27.9	9.26	0.0274
-0.8562	I1KW78	Uncharacterized protein	23	8	60.8	9.16	0.0349
-0.8563	I1LXW3	Transmembrane 9 superfamily member	16	7	74.7	5.78	0.0070
-0.8572	C6SVL3	Phytocyanin domain-containing protein	16	2	19.3	6.93	0.0170
-0.8575	I1L295	Uncharacterized protein	4	2	60.1	6.64	0.0026

-0.8578	A0A368UGT8	Uncharacterized protein	46	12	50.4	5.14	0.0074
-0.8626	I1MXA0	Uncharacterized protein	4	2	63.2	5.36	0.0006
-0.8671	C6SVC7	Uncharacterized protein	32	3	20.2	7.87	0.0023
-0.8672	I1L3Z7	Transmembrane 9 superfamily member	19	7	48.0	7.46	0.0002
-0.8716	A0A0R0GIU4	Peroxidase (EC 1.11.1.7)	24	5	35.2	7.83	0.0215
-0.8762	I1L340	C2 domain-containing protein	15	3	30.3	5.03	0.0242
-0.8773	I1KFE9	Uncharacterized protein	30	9	60.1	7.77	0.0026
-0.8799	A0A0R0LAT1	Protein kinase domain-containing protein	10	5	66.4	7.50	0.0348
-0.8800	K7K770	PWWP domain-containing protein	3	2	111.3	4.94	0.0449
-0.8838	K7L3Q8	Uncharacterized protein	12	5	53.5	6.20	0.0001
-0.8866	I1LBI3	Cysteine synthase (EC 2.5.1.47)	16	3	34.5	5.45	0.0479
-0.8872	I1KUJ7	Uncharacterized protein	6	2	58.4	6.46	0.0114
-0.8883	I1NBM7	Uncharacterized protein	32	10	55.5	6.73	0.0022
-0.8912	I1JIV1	Phytocyanin domain-containing protein	18	2	25.8	4.75	0.0008
-0.8942	I1K8W1	TPT domain-containing protein	5	2	38.6	8.84	0.0199
-0.8946	A0A0R0L3G5	Amino_oxidase domain-containing protein	5	3	66.8	7.87	0.0438
-0.8960	C6SVZ6	AAI domain-containing protein	47	3	12.4	7.96	0.0365
-0.8998	Q9SWA8	Glycine-rich RNA-binding protein	61	8	15.8	7.20	0.0046
-0.9000	Q96446	Actin (Fragment)	38	5	22.4	5.62	0.0059
-0.9007	C6T447	Uncharacterized protein	40	6	25.1	6.35	0.0317
-0.9009	A0A0R0HDJ7	PEROXIDASE_4 domain-containing protein	8	2	31.2	9.04	0.0174
-0.9041	I1LLM7	Asparagine synthetase	51	19	65.2	6.64	0.0195
-0.9050	D2D5G3	Methyltransferase (EC 2.1.1.-)	31	9	40.4	6.95	0.0362
-0.9144	C6SYP7	Dirigent protein	18	2	24.2	8.37	0.0050
-0.9196	A0A0R0FQV9	Xyloglucan endotransglucosylase/hydrolase	28	7	33.7	6.93	0.0295
-0.9221	Q9ZNZ5	Peroxidase (EC 1.11.1.7)	36	8	38.5	8.13	0.0040
-0.9227	I1MER4	PMR5N domain-containing protein	28	6	42.3	8.75	0.0441
-0.9269	C6T487	Uncharacterized protein	19	3	19.8	7.09	0.0005
-0.9269	I1M4U9	NAD(P)-bd_dom domain-containing protein	51	11	39.1	8.13	0.0070
-0.9295	A0A0R0IIS3	Cas1_AcylT domain-containing protein	4	2	67.1	8.81	0.0133
-0.9374	K7KDX4	Uncharacterized protein	4	2	71.3	8.43	0.0345
-0.9377	B9VJS7	Starch synthase IIa-2	7	4	86.5	6.28	0.0039
-0.9407	I1MPK1	Uncharacterized protein	6	2	49.9	5.58	0.0040
-0.9414	A0A0R4J3J1	Peroxidase (EC 1.11.1.7)	43	11	35.2	8.06	0.0114
-0.9415	C6TIR2	Uncharacterized protein	58	5	16.9	4.27	0.0030
-0.9434	I1JDK6	Uncharacterized protein	12	4	50.7	6.92	0.0051
-0.9435	I1KBS7	Uncharacterized protein	39	12	62.5	4.86	0.0053
-0.9452	I1LPL0	FAD-binding PCMH-type domain-containing protein	47	19	66.0	8.22	0.0015
-0.9459	K7MCL5	Flavin-containing monooxygenase (EC 1.-.-.-)	14	4	59.3	7.83	0.0226
-0.9519	Q2MJR5	Myo-inositol-1-phosphate synthase	56	13	56.4	5.54	0.0056
-0.9579	C6TGB8	Uncharacterized protein	36	10	46.2	6.10	0.0009
-0.9663	I1K0L4	Uncharacterized protein	34	15	71.7	6.00	0.0158
-0.9678	K7LSM2	Uncharacterized protein	41	45	153.5	4.72	0.0025
-0.9724	I1LLM2	Asparagine synthetase	51	19	65.2	6.64	0.0159
-0.9744	C6ZGA8	Somatic embryogenesis receptor kinase	11	3	68.9	5.73	0.0010
-0.9775	A0A0R0HYB1	Uncharacterized protein	59	18	51.6	8.34	0.0009
-0.9792	I1KL23	Uncharacterized protein	15	3	50.0	7.58	0.0096
-0.9820	C6TH72	Fe2OG dioxygenase domain-containing protein	25	4	32.9	5.43	0.0055
-0.9881	K7N544	CMP/dCMP-type deaminase	26	3	20.6	5.16	0.0324
-1.0002	I1M712	Oxygen-evolving enhancer protein 2	31	4	28.6	7.87	0.0330
-1.0018	C6T7J2	Uncharacterized protein	12	3	25.1	9.01	0.0156
-1.0049	C6TFS5	Cytochrome b6-f complex iron-sulfur subunit	32	5	28.4	8.79	0.0145
-1.0055	K7MPZ4	dUTPase domain-containing protein	43	3	15.7	5.53	0.0120
-1.0128	A0A0R4J339	Uncharacterized protein	18	6	82.3	8.54	0.0008
-1.0136	I1J4L0	Uncharacterized protein	5	2	58.2	8.65	0.0152
-1.0138	K7LQ40	Pyr_redox_2 domain-containing protein	9	2	39.4	8.54	0.0079
-1.0225	C6SYE7	Bet_v_1 domain-containing protein	55	6	17.6	6.52	0.0224

-1.0331	I1KB60	Uncharacterized protein	10	2	60.0	5.22	0.0165
-1.0345	I1JY06	Uncharacterized protein	33	17	91.7	5.31	0.0001
-1.0388	C6T1T3	Uncharacterized protein	35	4	22.4	6.07	0.0415
-1.0487	I1MBC5	Asparagine synthetase	57	21	65.5	6.47	0.0011
-1.0516	I1M9W9	Uncharacterized protein	4	2	107.0	7.27	0.0111
-1.0562	K7KRR7	X8 domain-containing protein	10	3	54.4	5.97	0.0025
-1.0601	C6THA9	NAD(P)-bd_dom domain-containing protein	51	11	39.1	8.13	0.0387
-1.0659	C6SYL5	SHSP domain-containing protein	20	2	22.2	6.21	0.0010
-1.0660	C6TDC0	Uncharacterized protein	11	3	37.0	6.18	0.0062
-1.0703	I1KQC0	Uncharacterized protein	15	6	68.8	8.95	0.0084
-1.0753	I1LU76	Peroxidase (EC 1.11.1.7)	32	7	35.8	8.22	0.0143
-1.0784	C6ZS11	Leucine-rich repeat resistance protein-like protein	12	3	37.1	8.38	0.0009
-1.0830	I1L8V8	Uncharacterized protein	45	5	17.7	8.70	0.0372
-1.1006	I1M3S8	Uncharacterized protein	4	3	85.7	5.12	0.0037
-1.1057	K7L413	Hexosyltransferase (EC 2.4.1.-)	6	3	75.9	8.92	0.0052
-1.1079	I1MTR7	DUF4005 domain-containing protein	14	6	65.0	9.47	0.0041
-1.1098	A0A0R0J569	Uncharacterized protein	13	3	46.0	7.78	0.0000
-1.1115	C6T8W8	PsbP domain-containing protein	11	2	28.4	7.87	0.0323
-1.1132	A0A0R0GF09	Uncharacterized protein	5	2	76.1	5.29	0.0289
-1.1179	I1KZH4	FKS1_dom1 domain-containing protein	2	3	225.6	9.09	0.0132
-1.1219	I1NHP0	Uncharacterized protein	16	9	117.7	6.87	0.0168
-1.1295	A0A0R0IA82	Peptidase A1 domain-containing protein	14	3	52.8	5.35	0.0017
-1.1319	I1LSM1	Phytocyanin domain-containing protein	20	3	22.8	6.92	0.0130
-1.1339	I1NIY9	Mitochondrial Rho GTPase (EC 3.6.5.-)	43	16	71.9	5.54	0.0081
-1.1380	C6THI3	PKS_ER domain-containing protein	58	12	39.1	6.84	0.0490
-1.1458	C6TNI2	Lipase_3 domain-containing protein	13	4	55.0	8.13	0.0011
-1.1459	C6SVL0	Uncharacterized protein	47	6	21.8	5.33	0.0222
-1.1488	I1KG57	Uncharacterized protein	32	9	46.6	5.88	0.0207
-1.1492	I1JBJ9	Endoglucanase (EC 3.2.1.4)	8	2	56.6	9.22	0.0138
-1.1507	I1KVS6	D-3-phosphoglycerate dehydrogenase (EC 1.1.1.95)	34	12	66.0	6.90	0.0069
-1.1553	A0A0R0L0K1	Uncharacterized protein	10	4	62.4	9.07	0.0009
-1.1569	I1M9S1	Plasma membrane ATPase (EC 7.1.2.1)	32	22	104.6	7.21	0.0133
-1.1655	I1MCZ5	LEA_2 domain-containing protein	12	2	27.9	10.27	0.0011
-1.1662	I1ND63	LRRNT_2 domain-containing protein	21	7	50.3	8.05	0.0189
-1.1674	I1KYQ1	Glycosyltransferase (EC 2.4.1.-)	7	2	51.5	5.92	0.0312
-1.1683	I1L3X9	Sucrose synthase (EC 2.4.1.13)	11	6	104.1	7.06	0.0263
-1.1813	I1LDH2	Tr-type G domain-containing protein	9	8	147.0	5.30	0.0492
-1.1853	C6TGB4	Uncharacterized protein	12	2	29.9	9.28	0.0433
-1.1901	A0A0R0GA40	Uncharacterized protein	13	3	50.8	6.01	0.0087
-1.1996	I1JWW4	Uncharacterized protein	12	2	31.1	5.12	0.0109
-1.2233	B0M194	Peroxisomal 3-hydroxyacyl-CoA dehydrogenase	12	2	35.0	9.45	0.0002
-1.2318	I1K510	Uncharacterized protein	18	8	93.7	5.17	0.0058
-1.2489	C6SX14	Ferredoxin	49	3	16.5	6.89	0.0047
-1.2535	A0A0R0KYU7	Xyloglucan endotransglucosylase/hydrolase	29	8	36.0	8.25	0.0024
-1.2553	K7MK51	Uncharacterized protein	7	4	87.6	6.98	0.0134
-1.2575	A0A0R4J2V8	Dirigent protein	28	3	23.9	6.28	0.0078
-1.2630	I1MJP8	Cellulose synthase (EC 2.4.1.12)	17	10	119.9	7.02	0.0070
-1.2642	I1K702	PLCXc domain-containing protein	26	5	36.2	8.02	0.0166
-1.2725	K7KCU6	Protein kinase domain-containing protein	6	3	92.0	8.16	0.0004
-1.2759	A0A0R0LFB4	Dihydrolipoamide acetyltransferase component of pyruvate dehydrogenase complex (EC 2.3.1.-)	21	8	47.9	8.70	0.0348
-1.2815	K7KZ57	Uncharacterized protein	5	2	51.6	6.42	0.0003
-1.2902	A8HTK0	Thioredoxin	51	4	13.3	5.53	0.0043
-1.2907	K7LJA2	Uncharacterized protein	8	2	51.2	6.02	0.0365
-1.2907	C6TGT5	Uncharacterized protein	9	2	37.7	7.03	0.0446
-1.3005	I1KD34	COBRA-like protein	14	3	50.7	8.60	0.0005
-1.3011	A0A0R0KSI5	TPR_REGION domain-containing protein	7	3	37.7	8.73	0.0081

-1.3121	A0A0R0JAB1	Auxin efflux carrier component	4	2	60.7	7.06	0.0043
-1.3207	C6SVM4	Uncharacterized protein	35	4	22.3	5.82	0.0080
-1.3253	A0A0R0K0E5	FAD-binding PCMH-type domain-containing protein	10	4	59.2	7.85	0.0062
-1.3388	I1MBL5	Uncharacterized protein	40	15	57.9	8.75	0.0002
-1.3414	A0A0R0JPK0	AAA domain-containing protein	5	4	133.9	6.65	0.0039
-1.3499	I1KSC1	Uncharacterized protein	11	5	66.5	8.19	0.0000
-1.3504	I1LCX5	Uncharacterized protein	8	3	67.3	6.49	0.0249
-1.3541	A0A0R0FND6	Elongation factor G, chloroplastic (cEF-G)	10	4	86.8	5.77	0.0401
-1.3587	I1M9K9	Uncharacterized protein	25	8	39.8	7.74	0.0030
-1.3613	C6ZRR4	NAK-type protein kinase	8	5	102.2	6.34	0.0001
-1.3631	I1KWE5	RRM domain-containing protein	10	2	26.2	4.86	0.0438
-1.3646	Q9SWB5	Seed maturation protein PM37	43	12	46.3	6.28	0.0335
-1.3819	K7N361	Lipase	11	3	49.2	7.23	0.0169
-1.3923	I1LA99	Uncharacterized protein	10	4	86.2	5.92	0.0003
-1.3936	C6SZA9	Bet_v_1 domain-containing protein	44	6	17.1	5.25	0.0381
-1.3946	Q2MJR4	Myo-inositol-1-phosphate synthase	40	9	56.4	5.67	0.0203
-1.3984	C6TMX0	Xyloglucan endotransglucosylase/hydrolase	31	8	34.4	8.95	0.0009
-1.3986	C6TD35	PABS domain-containing protein	9	2	37.0	5.40	0.0247
-1.4082	K7M8I6	Uncharacterized protein	6	3	60.9	6.55	0.0041
-1.4095	K7N057	Hexosyltransferase (EC 2.4.1.-)	10	3	61.8	8.21	0.0424
-1.4100	I1JG92	Uncharacterized protein	12	5	86.3	6.23	0.0181
-1.4130	I1N4W1	PMR5N domain-containing protein	24	6	51.3	8.51	0.0002
-1.4238	K7KQQ0	Uncharacterized protein	13	2	30.8	7.28	0.0009
-1.4250	C6TM76	DUF1279 domain-containing protein	15	2	20.6	9.32	0.0219
-1.4378	I1N6I1	Hexosyltransferase (EC 2.4.1.-)	8	3	63.1	8.48	0.0170
-1.4513	C6T736	V-SNARE domain-containing protein	42	6	24.7	9.50	0.0002
-1.4661	I1MS92	Uncharacterized protein	5	2	55.0	8.28	0.0040
-1.4664	I1L3E3	Uncharacterized protein	6	2	39.4	9.48	0.0023
-1.4795	I1L546	NmrA domain-containing protein	11	2	35.6	6.62	0.0051
-1.4930	A0A0R0IYY7	Uncharacterized protein	51	19	59.0	8.65	0.0023
-1.4941	K7LP97	RRM domain-containing protein	42	5	16.9	8.12	0.0031
-1.4980	C6SXA1	CS domain-containing protein	41	3	19.4	4.55	0.0325
-1.5002	I1L0Z7	Uncharacterized protein	6	6	168.3	7.47	0.0060
-1.5047	I1KEH1	Phytocyanin domain-containing protein	20	3	23.2	7.87	0.0024
-1.5144	K7KPK3	Uncharacterized protein	28	11	73.0	4.30	0.0096
-1.5167	I1JUE0	Cellulose synthase (EC 2.4.1.12)	28	19	122.0	6.76	0.0004
-1.5313	I1JT65	Uncharacterized protein	18	4	40.1	6.37	0.0019
-1.5410	A0A0R4J645	Uncharacterized protein	9	2	41.6	5.45	0.0390
-1.5523	C6SYN8	Uncharacterized protein	24	2	15.5	7.96	0.0111
-1.5613	I1LL85	Uncharacterized protein	5	2	60.7	8.48	0.0017
-1.5616	I1NII6	IPPc domain-containing protein	2	2	127.2	6.47	0.0420
-1.5666	C6TA63	Uncharacterized protein	7	2	47.8	7.42	0.0143
-1.5768	I1JXR7	Exostosin domain-containing protein	12	3	47.1	6.65	0.0020
-1.5769	Q06765	ADR6 protein (Sali5-4a protein)	52	8	30.4	6.84	0.0008
-1.5800	I1KNV8	Uncharacterized protein	14	7	95.1	5.14	0.0147
-1.5954	K7LCZ8	Cellulose synthase (EC 2.4.1.12)	20	11	120.1	7.39	0.0011
-1.5961	I1LZW4	Tr-type G domain-containing protein	16	11	109.9	5.24	0.0064
-1.6205	I1LCV9	Carboxypeptidase (EC 3.4.16.-)	16	6	56.0	5.38	0.0039
-1.6243	K7K6S5	AB hydrolase-1 domain-containing protein	16	2	29.3	5.60	0.0280
-1.6311	I1L7W1	Uncharacterized protein	4	4	167.6	8.15	0.0420
-1.6536	I1MUR9	GMC_OxRdtase_N domain-containing protein	6	3	62.9	6.84	0.0045
-1.6590	A0A0R0JQU1	Uncharacterized protein	34	5	32.9	4.93	0.0355
-1.6670	A0A0R0INP8	Peroxidase (EC 1.11.1.7)	29	7	34.5	8.90	0.0162
-1.6803	I1KRB5	FAD-binding PCMH-type domain-containing protein	33	12	58.8	8.70	0.0006
-1.6901	Q2LAL0	Cytochrome P450 monooxygenase CYP81E11	22	6	57.9	8.91	0.0129
-1.6975	I1MLV5	Fe2OG dioxygenase domain-containing protein	32	7	35.8	5.69	0.0420
-1.7055	I1LDY8	Uncharacterized protein	23	5	52.1	7.80	0.0004

-1.7130	I1J7C0	Uncharacterized protein	11	2	37.3	4.88	0.0022
-1.7390	I1KTE1	Cellulose synthase (EC 2.4.1.12)	12	8	123.9	6.93	0.0002
-1.7525	A0A0R0K0I4	X8 domain-containing protein	6	2	62.3	6.13	0.0105
-1.7569	A0A0R0FVX1	Uncharacterized protein	32	12	58.6	7.37	0.0002
-1.8050	I1JTX2	Uncharacterized protein	16	3	28.2	9.83	0.0285
-1.8576	I1JIW7	Uncharacterized protein	30	7	24.9	9.42	0.0020
-1.8765	A0A0R0EUW6	Uncharacterized protein	9	3	57.6	8.38	0.0019
-1.9205	C6SZ34	Uncharacterized protein	9	2	23.8	6.61	0.0114
-1.9238	I1LJ10	Peptidyl-prolyl cis-trans isomerase	11	2	26.1	7.34	0.0003
-1.9262	I1K2X6	FAD-binding PCMH-type domain-containing protein	30	11	59.5	9.03	0.0352
-1.9269	A0A0R0EHS8	PMR5N domain-containing protein	33	3	20.5	8.31	0.0129
-1.9454	K7M4N7	Uncharacterized protein	4	2	65.1	8.12	0.0272
-1.9505	I1LHJ6	SHNi-TPR domain-containing protein	23	6	50.1	4.54	0.0000
-1.9539	Q8S564	4-coumarate:coenzyme A ligase (EC 6.2.1.12)	26	9	59.3	5.91	0.0005
-1.9568	I1K8W4	Cellulose synthase (EC 2.4.1.12)	28	18	122.0	6.76	0.0177
-1.9569	I1JWM6	COBRA-like protein	10	2	50.6	8.68	0.0001
-1.9642	A0A0R0FEC7	Lipase_3 domain-containing protein	7	2	54.3	5.96	0.0324
-1.9841	C6TN54	Uncharacterized protein	10	2	47.1	7.08	0.0397
-1.9867	I1K3A6	Uncharacterized protein	10	4	64.3	6.57	0.0000
-2.0067	K7KEZ6	Uncharacterized protein	6	2	78.8	5.31	0.0229
-2.0407	C6TNZ5	Uncharacterized protein	12	2	33.0	6.35	0.0075
-2.0454	A8C8H5	Glutamate decarboxylase (EC 4.1.1.15)	59	19	57.2	5.82	0.0030
-2.0668	I1MY41	Uncharacterized protein	13	5	61.3	6.65	0.0004
-2.0698	I1ND18	Uncharacterized protein	9	3	73.8	6.83	0.0088
-2.0736	I1J7C7	RanBD1 domain-containing protein	21	3	25.6	4.72	0.0041
-2.0744	A0A024FRX1	Glycosyltransferase (EC 2.4.1.-)	10	3	52.2	6.14	0.0044
-2.0813	I1K6B1	NAB domain-containing protein	5	4	139.4	4.94	0.0444
-2.1017	I1MWI1	zinc_ribbon_10 domain-containing protein	9	2	44.8	8.84	0.0002
-2.1115	I1N843	Glyceraldehyde-3-phosphate dehydrogenase	47	14	43.2	8.25	0.0003
-2.1141	K7L4Y5	Pectinesterase (EC 3.1.1.11)	5	2	57.8	8.68	0.0278
-2.1160	I1LM80	Sucrose synthase (EC 2.4.1.13)	5	3	95.7	6.99	0.0142
-2.1251	C6TIQ7	Cytochrome c domain-containing protein	48	8	33.6	6.95	0.0302
-2.1467	I1M4H1	Carboxypeptidase (EC 3.4.16.-)	11	3	56.4	5.74	0.0317
-2.1473	C6SZQ8	Phytocyanin domain-containing protein	24	2	17.3	6.76	0.0004
-2.1571	A0A0R0F859	Epimerase domain-containing protein	28	6	35.1	5.83	0.0027
-2.1817	A0A0R0G7V6	Uncharacterized protein	5	2	62.8	6.99	0.0107
-2.1866	I1MXX3	Uncharacterized protein	21	2	16.5	9.31	0.0075
-2.1996	I1JBJ2	Uncharacterized protein	45	11	50.2	6.99	0.0157
-2.2028	I1KNP5	Endoglucanase (EC 3.2.1.4)	9	4	68.7	8.72	0.0392
-2.2043	A0A0R0HPC7	Pectate lyase (EC 4.2.2.2)	5	2	42.1	8.09	0.0012
-2.2179	K7MAF0	Lipase_GDSL domain-containing protein	8	2	41.3	5.91	0.0338
-2.2345	I1NAW5	Uncharacterized protein	15	3	50.5	6.71	0.0001
-2.2369	I1L0J1	Uncharacterized protein	5	2	52.9	5.16	0.0019
-2.2470	Q6Q2Z8	Phosphoenolpyruvate carboxylase (EC 4.1.1.31)	31	23	110.5	6.42	0.0083
-2.2561	I1JY96	Protein kinase domain-containing protein	6	3	113.6	7.11	0.0009
-2.2646	C6TF22	Uncharacterized protein	14	3	39.5	4.91	0.0317
-2.2818	I1MVV4	Uncharacterized protein	3	2	125.9	5.22	0.0181
-2.2828	I1JN04	Uncharacterized protein	11	4	57.7	8.53	0.0030
-2.3166	A0A0R0LFG1	Uncharacterized protein	4	2	118.6	6.55	0.0286
-2.3407	Q2LAL4	Cytochrome P450 monooxygenase CYP83E8	38	13	57.4	7.96	0.0130
-2.3421	I1LRM9	Uncharacterized protein	12	2	50.4	7.08	0.0082
-2.3426	A0A0R0HZ32	Uncharacterized protein	13	2	20.0	5.30	0.0175
-2.3620	I1KRH0	AAA domain-containing protein	9	2	45.4	6.29	0.0471
-2.3771	I1JNM0	PMR5N domain-containing protein	9	2	70.1	8.43	0.0021
-2.3948	I1MMC2	t-SNARE coiled-coil homology protein	18	3	30.0	6.32	0.0002
-2.4308	Q9XFI6	Peroxidase (EC 1.11.1.7)	23	5	37.4	8.35	0.0039
-2.4507	A0A024FRL5	Glycosyltransferase (EC 2.4.1.-)	8	2	52.0	5.92	0.0367

-2.4594	I1M3B6	Uncharacterized protein	3	2	131.6	7.34	0.0403
-2.4654	A0A0R0I5R9	Uncharacterized protein	38	12	48.0	6.52	0.0004
-2.5444	I1KZ04	Cytochrome b561 and DOMON protein	13	3	43.2	9.38	0.0001
-2.5473	I1K6Q2	Uncharacterized protein	12	3	48.3	5.78	0.0030
-2.5572	K7N525	ATP-grasp domain-containing protein	13	5	68.6	6.83	0.0429
-2.5629	A0A0R0L0I9	HTH myb-type domain-containing protein	2	2	46.7	6.84	0.0042
-2.5631	A0A0R0JLI3	Epimerase domain-containing protein	7	2	36.6	8.54	0.0090
-2.5955	C6T8K0	LEA_2 domain-containing protein	19	4	34.2	9.94	0.0001
-2.6228	C6T3V3	Glutathione peroxidase	18	3	19.1	9.61	0.0006
-2.6234	I1L158	Cellulose synthase (EC 2.4.1.12)	6	3	119.2	7.62	0.0045
-2.6305	I1KWS8	Glyco_hydro_19_cat domain-containing protein	10	2	36.0	6.27	0.0002
-2.6349	K7M1F7	Lipase_GDSL domain-containing protein	8	2	40.5	7.91	0.0014
-2.6350	I1KH95	Uncharacterized protein	5	2	93.0	8.76	0.0117
-2.6361	I1KW02	BURP domain-containing protein	12	2	30.2	7.94	0.0093
-2.6939	C6TK45	Uncharacterized protein	8	2	43.8	8.46	0.0000
-2.6972	C6TEQ7	Lipase_GDSL domain-containing protein	15	2	37.8	7.85	0.0028
-2.7071	I1ML02	Uncharacterized protein	8	4	69.9	6.62	0.0370
-2.7185	I1MFX5	Endoglucanase (EC 3.2.1.4)	13	6	68.3	8.66	0.0004
-2.7597	C6TL20	Lipase_GDSL domain-containing protein	31	7	40.2	6.10	0.0239
-2.7866	Q43468	Hsp70-Hsp90 organizing protein 1	17	7	64.1	5.69	0.0484
-2.7964	I1N641	Uncharacterized protein	16	14	136.1	7.99	0.0004
-2.8418	A0A0R0EB47	Uncharacterized protein	6	2	35.3	9.63	0.0003
-2.8662	I1JFT2	Glycosyltransferase (EC 2.4.1.-)	6	2	54.9	6.52	0.0008
-2.8832	A0A0R4J4M3	FAS1 domain-containing protein	13	2	27.8	8.88	0.0142
-3.0075	C6TD22	Glyco_hydro_19_cat domain-containing protein	15	3	36.4	6.51	0.0013
-3.0221	C6KHU4	Phenylalanine ammonia-lyase (EC 4.3.1.24)	41	16	78.1	6.19	0.0000
-3.0364	I1K0W4	Glutamate dehydrogenase	17	4	44.8	6.61	0.0042
-3.1029	I1LWJ6	Uncharacterized protein	17	14	136.0	7.99	0.0000
-3.1682	I1LRC1	Uncharacterized protein	21	9	62.5	9.11	0.0000
-3.1737	I1LH27	Tubulin alpha chain	56	20	49.7	5.06	0.0251
-3.1762	A0A0R4J551	Phytocyanin domain-containing protein	19	2	20.9	7.36	0.0001
-3.1764	A0A0R0FGJ1	Protein kinase domain-containing protein	4	2	95.1	6.23	0.0010
-3.1958	I1M9G4	Uncharacterized protein	8	2	41.2	8.56	0.0491
-3.2692	I1K345	AAA domain-containing protein	9	2	45.3	6.48	0.0173
-3.3016	K7LCS5	Flavin-containing monooxygenase (EC 1.-.-.-)	6	2	59.5	7.55	0.0219
-3.3052	I1K8B5	Uncharacterized protein	11	2	53.5	6.13	0.0151
-3.3169	Q70MR6	Ornithine decarboxylase (EC 4.1.1.17)	9	2	46.4	5.49	0.0254
-3.3921	I1M8S3	Uncharacterized protein	36	15	82.1	7.39	0.0008
-3.3979	A0A0R0IBM9	CMP/dCMP-type deaminase protein	19	3	20.7	5.86	0.0003
-3.4434	K7MR59	NAD(P)-bd_dom domain-containing protein	26	8	74.3	6.67	0.0316
-3.5126	I1JSP4	X8 domain-containing protein	10	2	48.3	5.16	0.0132
-3.5235	I1JIM1	WBb98N11.19 protein	5	2	64.5	6.62	0.0034
-3.5282	A1Z0H8	D-myo-inositol 3-phosphate synthase	50	12	56.4	5.39	0.0298
-3.5484	A0A0R4J2X1	Uncharacterized protein	8	2	44.0	8.34	0.0007
-3.5681	C6T5R3	PH domain-containing protein	27	2	16.8	8.65	0.0010
-3.5685	I1LVS4	Uncharacterized protein	27	9	61.7	5.20	0.0102
-3.6429	I1NHU7	Uncharacterized protein	10	2	39.0	6.29	0.0076
-3.6936	I1N019	Uncharacterized protein	12	4	72.2	6.25	0.0026
-3.6972	K7K8V7	C2 domain-containing protein	2	3	229.9	5.62	0.0283
-3.7284	A0A0R0I9A1	3-ketoacyl-CoA synthase (EC 2.3.1.-)	14	4	56.1	8.90	0.0005
-3.7480	I1MVG7	Uncharacterized protein	23	7	76.9	4.35	0.0011
-3.7670	I1K8I9	3-ketoacyl-CoA synthase (EC 2.3.1.-)	5	2	60.7	9.13	0.0003
-3.7712	I1KX04	Phospholipid-transporting ATPase (EC 7.6.2.1)	7	3	135.8	6.93	0.0032
-3.8203	I1L5J4	Uncharacterized protein	7	2	45.5	8.72	0.0008
-3.8488	C6TKJ2	Xyloglucan endotransglucosylase/hydrolase	35	7	35.6	8.37	0.0018
-3.8551	K7MNZ5	Uncharacterized protein	15	3	40.4	6.70	0.0081
-3.9002	I1M8D6	X8 domain-containing protein	7	2	51.2	5.19	0.0286

-3.9470	I1KPJ3	Endoglucanase (EC 3.2.1.4)	6	3	68.7	8.72	0.0019
-4.0320	I1M228	Eukaryotic translation initiation factor 3 subunit D	38	11	63.7	5.69	0.0050
-4.1000	I1MXJ1	Uncharacterized protein	36	15	82.2	7.09	0.0005
-4.1306	I1JT08	Uncharacterized protein	10	3	81.4	6.67	0.0045
-4.1386	A0A0R4J3Q3	ABC transporter domain-containing protein	33	4	28.3	5.90	0.0000
-4.2154	I1LTU5	Uncharacterized protein	18	6	48.5	7.47	0.0009
-4.2554	A0A0R0F5B0	Epimerase domain-containing protein	10	2	48.3	9.67	0.0305
-4.2622	I1NGD0	Lipoxygenase (EC 1.13.11.-)	12	6	97.9	6.70	0.0060
-4.3929	I1KCA8	Elongation factor Tu	39	13	52.1	6.68	0.0019
-4.3964	I1MUQ1	ThiF domain-containing protein	36	9	46.7	4.75	0.0001
-4.4399	I1KYC4	Peptidase A1 domain-containing protein	15	4	51.9	8.07	0.0020
-4.4419	A0A0R0I8A1	Xyloglucan endotransglucosylase/hydrolase	41	7	29.9	7.96	0.0036
-4.4849	I1JDD7	Cellulose synthase (EC 2.4.1.12)	11	7	121.4	7.18	0.0004
-4.5580	I1LKW4	Uncharacterized protein	21	9	62.4	9.11	0.0025
-4.5689	C6T020	Glutathione peroxidase	33	3	18.7	5.72	0.0002
-4.6311	K7MQS3	Peptidase A1 domain-containing protein	16	3	52.0	8.12	0.0002
-4.6353	I1LZ94	Phenylalanine ammonia-lyase (EC 4.3.1.24)	38	15	78.1	6.10	0.0158
-4.7179	C6TKY7	Str_synth domain-containing protein	8	2	38.8	7.81	0.0177
-4.7451	I1MC22	Lipase_GDSL domain-containing protein	10	2	39.9	7.85	0.0001
-4.8272	I1NA96	Phenylalanine ammonia-lyase (EC 4.3.1.24)	28	11	77.7	6.49	0.0002
-4.8630	K7N4G1	Uncharacterized protein	33	10	46.6	6.79	0.0002
-5.0003	Q70MR5	Ornithine decarboxylase (EC 4.1.1.17)	9	2	46.3	5.60	0.0016
-5.1048	I1LPJ0	ATP-dependent Clp protease proteolytic subunit	31	4	25.9	7.17	0.0009
-5.1694	I1N0I7	t-SNARE coiled-coil homology protein	16	3	28.1	8.90	0.0000
-5.4933	A0A0R0GCM7	CMP/dCMP-type deaminase protein	19	3	20.6	6.13	0.0119
-5.8435	A0A0R0FB78	Uncharacterized protein	33	8	45.8	8.43	0.0006
-5.9738	A0A0R0KUR1	Phenylalanine ammonia-lyase (EC 4.3.1.24)	20	9	77.9	6.42	0.0002
-6.0109	K7MPG5	Uncharacterized protein	28	6	42.4	8.29	0.0002
-6.4346	I1M5G8	Malic enzyme	27	9	71.3	7.55	0.0025
-7.1872	I1LJF7		8	3	56.5	9.38	0.0000
-10.9263	A0A0R0EIR6	Glycosyltransferase	36	9	54.4	5.92	0.0000

Abbreviation means as follows: Cov, coverage; MP, matched protein; MW, molecular weight; Cal, calculated.
Grey background is to proteins not considered to be differentially accumulated.

Supplemental Table S2. List of changed proteins in mutant soybean under flooding compared with non-flooding

Fold change	Accession	Protein names	Cov (%)	MP	MW (kDa)	Calc pI	p-value
9.6946	Q0WX05	Polygalacturonase inhibiting protein	50	10	36.8	8.34	0.0002
9.5204	C6T390	Uncharacterized protein	55	6	16.2	5.91	0.0001
7.7448	A0A0R0GEW3	Uncharacterized protein	11	3	30.6	6.80	0.0004
7.7396	Q42785	Nonsymbiotic hemoglobin	22	3	18.0	8.92	0.0000
6.7396	I1JY29	Uncharacterized protein	42	10	41.2	6.00	0.0003
6.5628	I1NF01	Uncharacterized protein	13	8	80.1	6.13	0.0000
6.3346	Q0WX04	Polygalacturonase inhibiting protein	19	3	37.5	8.91	0.0004
6.2153	C6TMH8	Ribosomal protein L19	21	2	24.7	11.44	0.0004
5.8204	I1LJK0	Uncharacterized protein	52	3	7.9	6.60	0.0010
5.7755	I1L3I1	Lectin_legB domain-containing protein	12	3	33.2	5.10	0.0002
5.6935	C6TFF5	Uncharacterized protein	26	2	11.1	10.45	0.0184
5.6256	A0A0R0K553	Fe2OG dioxygenase domain-containing protein	38	8	34.8	5.58	0.0018
5.6147	A0A0R0KF48	Uncharacterized protein	41	14	65.2	6.21	0.0000
5.4557	A0A0R0IID0	Uncharacterized protein	31	4	24.0	6.02	0.0006
5.4388	C6TMH1	ZnMc domain-containing protein	12	2	40.1	5.63	0.0012
5.4217	C6TDV4	LRRNT_2 domain-containing protein	7	2	36.2	8.43	0.0001
5.2687	I1KR99	LRRNT_2 domain-containing protein	16	3	37.4	7.91	0.0000
4.5604	I1MQG3	Uncharacterized protein	28	3	22.2	5.21	0.0034
4.3185	A0A0R0KXL5	Uncharacterized protein	23	2	13.6	8.18	0.0124
4.2159	C6TC19	Rhodanese domain-containing protein	13	2	16.4	8.25	0.0006
4.0563	I1LL87	Uncharacterized protein	18	2	19.3	4.96	0.0340
4.0260	I1LT24	DUF3700 domain-containing protein	33	6	28.0	6.68	0.0000
3.9997	C6TLA6	DUF3700 domain-containing protein	20	3	27.5	7.37	0.0045
3.8885	I1JAL3	Uncharacterized protein	9	3	61.0	8.68	0.0065
3.7787	I1MAE6	PKS_ER domain-containing protein	39	11	40.8	6.52	0.0000
3.7308	C6T2R5	RNase H domain-containing protein	14	2	26.7	7.34	0.0001
3.6458	I1MBS4	Usp domain-containing protein	27	3	18.0	9.41	0.0000
3.6182	Q42447	Maturation protein (Maturation-associated protein)	27	3	25.6	6.52	0.0001
3.4729	I1JQU3	AMP-binding domain-containing protein	6	2	74.4	7.18	0.0010
3.4605	I1JS15	Uncharacterized protein	5	3	116.8	6.58	0.0307
3.4392	C6TCK6	Uncharacterized protein	13	2	25.8	6.86	0.0003
3.4120	C6THW8	DUF3700 domain-containing protein	40	7	28.0	6.61	0.0002
3.3355	I1JZK8	t-SNARE coiled-coil homology protein	10	2	33.3	7.14	0.0002
3.2705	C6TN86	Uncharacterized protein	10	2	38.3	6.00	0.0000
3.1554	A0A0R0FZI3	AB hydrolase-1 domain-containing protein	9	2	33.6	6.54	0.0094
3.1463	A0A0R0F3E0	Histone H2A	21	2	15.5	10.71	0.0082
3.0952	K7KSU9	GYF domain-containing protein	2	2	197.7	6.47	0.0040
3.0592	I1LYM0	DUF1681 domain-containing protein	23	4	30.7	6.68	0.0016
3.0085	C6TKX3	Uncharacterized protein	8	2	27.2	6.40	0.0245
2.9768	I1MN40	VWFA domain-containing protein	15	3	42.4	4.73	0.0046
2.9512	I1N846	Uncharacterized protein	27	11	83.9	6.73	0.0147
2.9493	C6T229	Uncharacterized protein	32	2	8.0	8.84	0.0351
2.9102	I1KFZ1	Smg4_UPF3 domain-containing protein	8	2	58.4	10.02	0.0157
2.8082	I1KSE3	ALOG domain-containing protein	20	2	18.8	9.88	0.0056
2.7457	A0A0R4J2I4	Uncharacterized protein	43	16	65.6	5.94	0.0003
2.7137	I1MEX0	MPN domain-containing protein	57	8	34.6	6.71	0.0002
2.6910	A0A024BT07	Acyl-[acyl-carrier-protein] desaturase (EC 1.14.19.-)	12	4	44.0	7.23	0.0011
2.6847	C6TB96	Uncharacterized protein	22	5	35.2	5.49	0.0001
2.6403	A0A0R4J364	Uncharacterized protein	67	16	40.9	6.40	0.0008
2.6399	K7MEI2	Exocyst subunit Exo70 family protein	8	2	75.6	6.00	0.0331
2.6338	K7LDT9	Uncharacterized protei	9	5	103.3	6.76	0.0002
2.5196	K7MVZ6	Glucose-6-phosphate isomerase (EC 5.3.1.9)	37	10	62.7	5.72	0.0002
2.5094	K7MPT4	TPR_REGION domain-containing protein	6	2	59.8	7.37	0.0073

2.5032	I1MEH8	Uncharacterized protein	41	6	22.2	7.20	0.0006
2.4515	I1LC12	SHSP domain-containing protein	30	4	22.4	6.02	0.0166
2.4431	K7MID0	Uncharacterized protein	8	4	96.9	6.39	0.0131
2.4406	I1KQV7	Uncharacterized protein	38	19	72.5	6.04	0.0002
2.4194	I1JCQ0	Amino_oxidase domain-containing protein	11	4	60.8	6.90	0.0143
2.4079	I1J6C3	Arf-GAP domain-containing protein	34	8	43.2	8.92	0.0001
2.3830	I1K9P4	ZF-HD dimerization-type domain-containing protein	18	2	32.4	6.52	0.0016
2.3471	I1MR73	Uncharacterized protein	4	2	58.0	8.90	0.0034
2.3384	I1KJ00	Uncharacterized protein	14	3	28.2	5.43	0.0008
2.3279	I1JK18	Translocase of chloroplast (EC 3.6.5.-)	23	4	34.9	9.33	0.0138
2.2651	I1NHI7	VHS domain-containing protein	6	7	222.6	7.78	0.0217
2.2449	I1MCF0	Uncharacterized protein	23	5	45.2	6.43	0.0073
2.2247	Q01527	Maturation protein	38	2	15.6	9.66	0.0188
2.2178	K7MM61	Uncharacterized protein	3	5	256.7	6.20	0.0080
2.2098	Q9ZTY1	35 kDa seed maturation protein	10	2	35.3	6.32	0.0008
2.1948	K7LLK0	Uncharacterized protein	19	9	88.2	5.15	0.0077
2.1941	I1K9W9	Uncharacterized protein	14	2	34.7	8.54	0.0191
2.1798	A0A0R0GIX5	Nicotinamide-nucleotide adenylyltransferase	15	2	31.7	6.73	0.0482
2.1691	C6TME1	Uncharacterized protein	32	9	35.8	9.58	0.0000
2.1608	I1KUE9	WD_REPEATS_REGION domain-containing protein	10	2	38.8	8.22	0.0009
2.1588	C6T063	ANK_REP_REGION domain-containing protein	36	2	16.8	4.63	0.0088
2.1461	I1MUP6	Uncharacterized protein	47	14	46.4	5.11	0.0001
2.1243	I1JE40	Uncharacterized protein	35	16	68.9	7.36	0.0309
2.1204	I1M138	Peptidase A1 domain-containing protein	10	3	51.2	8.00	0.0248
2.1148	A0A0R0ENW6	Peptidase A1 domain-containing protein	35	8	46.3	8.53	0.0112
2.1095	A0A0R0KSG7	Ribosomal protein L19	17	2	24.7	11.40	0.0309
2.0244	K7MFQ7	Uncharacterized protein	15	6	68.8	8.50	0.0097
2.0101	I1LF63	Importin N-terminal domain-containing protein	30	14	99.4	5.05	0.0465
1.9453	I1L3K7	Uncharacterized protein	64	17	48.0	6.29	0.0000
1.9446	I1M7P3	Uncharacterized protein	34	2	14.7	4.93	0.0000
1.9380	I1MV69	Uncharacterized protein	25	4	27.5	9.20	0.0005
1.9057	A0A0R4J5T8	Uncharacterized protein	11	4	75.0	6.54	0.0047
1.8982	A0A0R4J4L3	Annexin	31	9	35.9	7.91	0.0250
1.8830	Q9SEL0	Seed maturation protein PM24	20	3	26.8	5.33	0.0009
1.8620	A0A0R0F259	Uncharacterized protein	41	14	65.2	6.21	0.0002
1.8617	I1J8S7	Uncharacterized protein	46	7	29.3	6.73	0.0028
1.8551	I1JPH6	Lambda class glutathione S-transferase	50	7	26.8	6.01	0.0042
1.8475	I1K301	Uncharacterized protein	59	7	14.8	5.71	0.0015
1.8379	I1N627	PHB domain-containing protein	26	6	31.5	5.24	0.0022
1.8224	A0A0R4J3N3	SHSP domain-containing protein	32	3	18.2	7.50	0.0341
1.8084	I1M1V8	Uncharacterized protein	56	18	63.4	6.11	0.0001
1.8043	I1NFP9	PB1 domain-containing protein	19	6	78.2	6.30	0.0124
1.8003	K7KGC7	Sucrose synthase (EC 2.4.1.13)	41	31	92.2	6.13	0.0000
1.7961	I1JU00	SHSP domain-containing protein	15	2	17.9	7.18	0.0007
1.7895	C6TFW0	Uncharacterized protein	37	3	13.3	9.16	0.0003
1.7668	I1N9W2	WD_REPEATS_REGION domain-containing protein	5	2	70.5	6.64	0.0186
1.7668	C6SVT0	Usp domain-containing protein	42	6	18.0	7.15	0.0001
1.7482	K7LNG2	NmrA domain-containing protein	53	12	41.8	8.21	0.0005
1.7057	A0A0R4J3M4	Uncharacterized protein	41	15	65.1	6.27	0.0004
1.7025	K7MZJ0	Sucrose synthase (EC 2.4.1.13)	47	33	92.2	6.38	0.0001
1.6994	C6SVM2	Uncharacterized protein	27	2	17.4	8.94	0.0004
1.6939	I1MWJ3	Uncharacterized protein	19	2	19.0	9.48	0.0387
1.6896	K7L1E9	Fe2OG dioxygenase domain-containing protein	15	3	34.7	5.71	0.0100
1.6649	P04670	Uricase-2 isozyme 1	46	12	35.1	7.97	0.0025
1.6635	K7LKJ0	HMA domain-containing protein	48	3	8.6	6.60	0.0007
1.6528	I1JSX4	AAA domain-containing protein	18	8	74.1	6.19	0.0077
1.6458	C6TEY7	Ubiquitin thioesterase (EC 3.4.19.12)	39	6	33.8	4.91	0.0281

1.6396	I1K7R0	Uncharacterized protein	27	3	16.1	5.35	0.0090
1.6056	I1JFL5	Inhibitor I9 domain-containing protein	26	3	14.7	6.52	0.0007
1.5807	I1K1B0	Uncharacterized protein	13	2	34.2	5.02	0.0340
1.5761	I1MX49	Uncharacterized protein	22	6	46.0	6.35	0.0288
1.5517	I1JFD2	BSD domain-containing protein	24	6	50.4	4.53	0.0175
1.5431	I1K850	Uncharacterized protein	15	5	82.5	8.88	0.0364
1.5203	A0A0R0JD83	Ligase_CoA domain-containing protein	35	15	63.1	6.71	0.0349
1.5200	I1LZK9	Uncharacterized protein	17	5	65.4	6.87	0.0151
1.5094	A0A0R0KL81	Uncharacterized protein	7	3	80.9	7.14	0.0053
1.5066	C6TEE9	Uncharacterized protein	55	10	30.4	6.09	0.0003
1.5041	A0A0R0GPZ3	SHSP domain-containing protein	16	2	17.6	5.97	0.0278
1.4953	K7K3E3	Uncharacterized protein	4	3	147.9	5.27	0.0145
1.4737	K7KVD9	Uncharacterized protein	4	2	73.5	6.42	0.0317
1.4380	C6K8D1	Seed biotinylated protein 68 kDa isoform	23	9	67.9	6.58	0.0026
1.4303	K7M5G4	GYF domain-containing protein	3	3	205.6	6.46	0.0053
1.4286	I1JKK8	SEC7 domain-containing protein	9	11	198.7	5.66	0.0036
1.4266	I1KKH2	Importin N-terminal domain-containing protein	6	3	114.8	5.68	0.0014
1.4158	I1NA37	Uncharacterized protein	35	4	21.0	6.93	0.0173
1.4130	I1MKC8	Starch synthase, chloroplastic/amyloplastic (EC 2.4.1.-)	26	10	68.1	7.06	0.0001
1.4100	K7L6T7	Protein kinase domain-containing protein	7	4	73.6	7.24	0.0168
1.4078	A0A0R0JL19	Uncharacterized protein	70	17	41.0	6.40	0.0002
1.4057	I1MUR0	UBX domain-containing protein	20	8	65.5	4.73	0.0008
1.3947	A0A0R4J4G9	SHSP domain-containing protein	22	3	23.7	6.98	0.0055
1.3736	Q9SWB2	Seed maturation protein PM41	31	2	8.2	4.97	0.0470
1.3723	M1FIS9	Ribosomal protein S4	11	3	41.3	11.22	0.0038
1.3612	I1KXF9	Carbonic anhydrase	20	4	28.1	6.77	0.0258
1.3576	C6TNN5	ZnMc domain-containing protein	29	7	43.4	6.10	0.0005
1.3452	I1M7N8	Uncharacterized protein	20	5	32.7	4.96	0.0085
1.3402	I1KWD8	Uncharacterized protein	36	7	49.8	5.27	0.0350
1.3386	A1YZE0	Starch synthase, chloroplastic/amyloplastic (EC 2.4.1.-)	24	9	68.0	7.06	0.0006
1.3218	I1JPH2	Uncharacterized protein	21	2	20.2	6.93	0.0274
1.3125	I1L7V7	Usp domain-containing protein	30	2	17.2	7.87	0.0270
1.2985	Q9S7N8	Seed maturation protein PM21	48	2	10.1	5.01	0.0012
1.2956	A0A0R4J2Q1	Peptidase A1 domain-containing protein	27	8	46.4	8.34	0.0003
1.2867	A0A0R0FSB9	Uncharacterized protein	49	2	11.2	9.20	0.0416
1.2828	K7K676	AP-1 complex subunit gamma	21	10	95.5	6.51	0.0069
1.2499	K7KHI6	Bifunctional dihydrofolate reductase-thymidylate synthase	15	5	59.7	6.73	0.0292
1.2465	I1J9W0	40S ribosomal protein S30	11	2	11.6	12.07	0.0002
1.2405	C6T9H6	Uncharacterized protein	26	5	42.3	7.56	0.0089
1.2388	I1M2S7	Lactamase_B domain-containing protein	47	15	41.4	8.72	0.0016
1.2313	A0A0R0EX12	Histone H4	21	4	17.9	11.36	0.0043
1.2312	I1NDA1	Uncharacterized protein	25	4	31.8	6.18	0.0058
1.2263	A0A0R0KE56	Pectinesterase (EC 3.1.1.11)	27	5	32.2	8.70	0.0258
1.2232	Q9XER5	Seed maturation protein PM22	36	4	16.7	5.36	0.0022
1.2133	I1L957	Uncharacterized protein	33	11	48.8	6.42	0.0004
1.2116	K7MIR3	Enolase_C domain-containing protein	67	15	38.3	6.80	0.0001
1.1965	O23959	Ca+2-binding EF hand protein	23	4	27.0	6.44	0.0235
1.1741	I1J7T0	Pectinesterase (EC 3.1.1.11)	20	7	56.5	9.01	0.0245
1.1706	C6T1V2	SHSP domain-containing protein	46	4	17.7	6.81	0.0050
1.1700	A0A0R0L186	SHSP domain-containing protein	18	3	17.3	7.42	0.0415
1.1558	I1M3D5	Arf-GAP domain-containing protein	12	4	52.3	8.43	0.0028
1.1315	I1LTF0	Uncharacterized protein	14	3	51.8	8.78	0.0069
1.1297	C6T1S9	Uncharacterized protein	23	2	13.6	10.45	0.0049
1.1225	I1J4D5	Uncharacterized protein	41	6	26.6	5.90	0.0084
1.1164	K7MFC0	GYF domain-containing protein	5	7	179.2	6.23	0.0025
1.1142	A0A0R4J4X6	SHSP domain-containing protein	39	6	18.5	8.34	0.0103

1.1137	K7LRE0	Uncharacterized protein	24	4	10.3	5.21	0.0039
1.0920	I1KS18	Uncharacterized protein	25	4	25.3	6.52	0.0141
1.0914	C6T3U6	Ribosomal protein L19	17	2	24.8	11.44	0.0019
1.0870	A0A0R0H3L4	Poly [ADP-ribose] polymerase	40	21	91.6	5.59	0.0052
1.0746	I1KG22	Uncharacterized protein	10	4	84.8	5.94	0.0031
1.0689	I1K964	Proteasome subunit beta (EC 3.4.25.1)	78	11	25.1	5.55	0.0321
1.0657	A0A368UH41	TOG domain-containing protein	14	9	118.8	6.76	0.0380
1.0544	I1NEU8	Aminotran_5 domain-containing protein	23	6	49.5	7.31	0.0055
1.0495	K7L8B5	Uncharacterized protein	42	5	15.3	4.64	0.0000
1.0418	I1JH91	Uncharacterized protein	5	2	92.3	6.65	0.0254
1.0313	K7LM54	Rab-GAP TBC domain-containing protein	15	8	93.2	4.83	0.0067
1.0219	C6T244	Uncharacterized protein	30	2	14.0	5.17	0.0021
1.0211	A0A0R0EXK4	Uncharacterized protein	15	7	70.7	8.37	0.0309
1.0086	I1KY70	Uncharacterized protein	5	2	86.0	6.02	0.0009
1.0072	I1MD29	PHD-type domain-containing protein	15	2	28.1	4.84	0.0245
1.0057	I1JLC8	Protein SLE2	35	3	11.5	5.60	0.0098
1.0056	I1KW96	Uncharacterized protein	3	2	117.1	8.57	0.0013
1.0027	C6TK48	Uncharacterized protein	7	2	44.9	5.57	0.0100
0.9997	I1KX25	Arf-GAP domain-containing protein	47	11	43.4	8.92	0.0014
0.9960	A0A0R0GA14	SHSP domain-containing protein	30	4	17.9	7.50	0.0182
0.9947	A0A0R0GZ36	Uncharacterized protein	48	9	41.3	5.99	0.0111
0.9919	Q8RVH5	Basic 7S globulin 2	40	8	47.2	8.25	0.0085
0.9871	Q9XET0	Seed maturation protein PM30	30	4	15.1	8.90	0.0255
0.9845	I1KMW4	TPR_REGION domain-containing protein	12	4	50.0	8.18	0.0002
0.9757	I1MB74	Uncharacterized protein	4	2	92.0	6.64	0.0177
0.9685	Q39873	Lea protein	22	8	49.4	7.52	0.0015
0.9632	C6T1E1	t-SNARE coiled-coil homology protein	44	4	14.4	5.90	0.0046
0.9626	I1JS10	Uncharacterized protein	86	2	6.8	8.92	0.0065
0.9605	I1LN49	Uncharacterized protein	50	8	27.0	7.06	0.0001
0.9603	C6SWV4	Uncharacterized protein	40	5	18.8	6.32	0.0109
0.9597	I1K517	Uncharacterized protein	11	3	47.0	5.53	0.0043
0.9493	K7KBB0	Phi class glutathione S-transferase	39	6	24.8	6.30	0.0342
0.9468	I1LKD9	Protein transport protein Sec61 subunit beta	41	4	10.9	11.65	0.0295
0.9417	I1M0K3	Cysteine proteinase inhibitor	50	9	27.7	7.08	0.0035
0.9391	C6SVX0	SHSP domain-containing protein	39	6	18.5	6.09	0.0114
0.9390	A0A0R0G8Z2	Cysteine proteinase inhibitor	13	2	22.5	5.30	0.0280
0.9329	C6T374	VOC domain-containing protein	61	4	15.4	5.69	0.0122
0.9323	I1KJK0	HABP4_PAI-RBP1 domain-containing protein	29	7	39.3	6.19	0.0444
0.9304	I1LNT7	Uncharacterized protein	6	4	83.7	6.01	0.0146
0.9296	K7MQM3	C2 domain-containing protein	26	8	46.5	4.67	0.0316
0.9288	I1KCD7	Uncharacterized protein	40	26	90.9	5.24	0.0280
0.9206	C6TFW8	Uncharacterized protein	18	3	25.9	7.30	0.0020
0.9172	I1K0C8	UBX domain-containing protein	26	9	65.7	4.73	0.0005
0.9158	K7MXK9	Hydrolase_4 domain-containing protein	13	4	77.3	7.94	0.0287
0.9120	I1MKT8	Glycosyltransferase (EC 2.4.1.-)	29	8	54.4	6.84	0.0011
0.9099	I1J5P9	Glycosyltransferase (EC 2.4.1.-)	28	8	56.1	5.88	0.0233
0.9045	I1JEB4	ANK_REP_REGION domain-containing protein	34	6	26.0	5.12	0.0116
0.9045	I1K7F5	MaoC-like domain-containing protein	13	3	34.1	8.53	0.0119
0.8992	I1MX00	Protein kinase domain-containing protein	9	4	75.5	6.42	0.0389
0.8971	A0A0R4J387	Chalcone-flavonone isomerase family protein	62	8	23.5	5.00	0.0022
0.8956	I1NG02	Thioredoxin domain-containing protein	31	4	24.4	5.57	0.0015
0.8923	C6T9B1	cobW domain-containing protein	53	9	31.4	6.48	0.0052
0.8918	A0A0R0E8N5	Uricase (EC 1.7.3.3) (Urate oxidase)	62	15	35.1	8.29	0.0002
0.8874	I1KHE4	TPR_REGION domain-containing protein	9	2	33.1	8.98	0.0011
0.8808	A0A0R0ES33	Uncharacterized protein	32	6	37.2	6.65	0.0155
0.8805	I1LR42	Uncharacterized protein	5	3	93.8	4.59	0.0090
0.8788	Q39801	51 kDa seed maturation protein	25	10	51.0	7.11	0.0039

0.8691	B3TDK6	Lipoxygenase (EC 1.13.11.-)	73	45	96.7	6.58	0.0008
0.8657	K7MS29	PPDK_N domain-containing protein	22	20	164.0	6.77	0.0052
0.8656	K7MR36	Citrate synthase (EC 2.3.3.16)	30	9	56.6	9.38	0.0332
0.8631	C6SV68	Cysteine proteinase inhibitor	31	2	11.4	6.06	0.0130
0.8629	C6SZK3	Glutathione peroxidase	44	5	18.4	7.09	0.0037
0.8626	I1KGM8	Uncharacterized protein	5	2	66.3	7.64	0.0171
0.8625	A0A0R0KRH5	Uncharacterized protein	12	3	44.7	4.82	0.0191
0.8574	I1N5N0	Pyruvate kinase (EC 2.7.1.40)	55	19	55.2	7.56	0.0012
0.8532	I1LDP2	Uncharacterized protein	16	4	27.9	8.92	0.0036
0.8471	A0A0R4J3K7	Epimerase domain-containing protein	11	3	35.8	6.86	0.0153
0.8454	A0A0R4J3X4	SHSP domain-containing protein	31	4	25.6	7.49	0.0204
0.8426	Q8W198	Phosphate transporter	36	12	36.3	9.04	0.0012
0.8420	C6T9B7	Uncharacterized protein	21	5	31.6	7.24	0.0012
0.8419	C6SYZ8	Uncharacterized protein	23	5	23.6	10.32	0.0020
0.8390	I1K7Z6	VID27 domain-containing protein	10	4	70.4	5.58	0.0118
0.8384	C6SXR9	Uncharacterized protein	15	3	26.1	8.05	0.0332
0.8337	K7KRK2	Uncharacterized protein	17	3	31.5	6.44	0.0448
0.8320	I1LP93	Phospholipid scramblase	12	3	40.2	9.57	0.0160
0.8297	A0A0R0EEE4	HMA domain-containing protein	32	3	13.6	7.72	0.0065
0.8213	K7KP23	Uncharacterized protein	5	2	60.5	9.00	0.0073
0.8202	I1MDA0	Uncharacterized protein	19	2	17.1	10.17	0.0005
0.8158	I1KJM6	Uncharacterized protein	24	7	55.2	8.10	0.0048
0.8129	I1JE17	Glycosyltransferase (EC 2.4.1.-)	50	13	53.1	5.86	0.0003
0.8107	A0A0R0GAY5	Peroxidase (EC 1.11.1.7)	7	2	37.8	7.94	0.0103
0.8068	C6SXY3	Glutaredoxin domain-containing protein	17	2	18.1	6.32	0.0012
0.7970	I1NDG9	Uncharacterized protein	9	2	43.1	5.95	0.0401
0.7962	I1KZ41	Glycosyltransferase (EC 2.4.1.-)	21	5	54.3	6.74	0.0034
0.7957	I1JV82	TPR_REGION domain-containing protein	32	4	24.1	8.70	0.0043
0.7956	A0A0R0HTR7	Uncharacterized protein	6	3	76.9	8.28	0.0297
0.7940	I1KYL4	ER membrane protein complex subunit 3	26	5	27.8	8.97	0.0060
0.7900	K7MHI8	G-patch domain-containing protein	7	3	98.7	5.74	0.0060
0.7898	A0A0R0G0T0	PfkB domain-containing protein	33	9	37.9	5.63	0.0025
0.7846	A0A0R0J624	Uncharacterized protein	10	2	27.2	10.08	0.0004
0.7830	C6SXW1	Peptide-methionine (R)-S-oxide reductase	41	3	15.1	6.74	0.0192
0.7823	I1MYR9	Uncharacterized protein	24	3	20.0	10.36	0.0457
0.7813	A0A0R0IVF1	Uncharacterized protein	23	7	48.0	8.22	0.0057
0.7760	I1M307	Bulb-type lectin domain-containing protein	19	7	48.8	6.64	0.0344
0.7746	I1MBI9	Peroxidase (EC 1.11.1.7)	26	7	40.0	9.54	0.0224
0.7688	I1MYV2	SAP domain-containing protein	14	6	78.0	4.74	0.0157
0.7646	I1KEY3	J domain-containing protein	19	4	38.4	9.04	0.0173
0.7625	C6SY82	Eukaryotic translation initiation factor 5A (eIF-5A)	22	3	17.3	6.20	0.0163
0.7588	C6SW77	Uncharacterized protein	34	5	25.8	9.69	0.0378
0.7586	I1L999	Uncharacterized protein	12	2	19.2	9.86	0.0494
0.7550	I1L2S8	Uncharacterized protein	10	2	33.2	4.63	0.0001
0.7517	I1K811	Uncharacterized protein	13	4	51.3	6.52	0.0310
0.7505	K7MP42	Ubiquitin-like domain-containing protein	14	9	98.7	5.43	0.0428
0.7488	I1MMG2	Uncharacterized protein	20	5	40.8	5.92	0.0436
0.7465	Q9LLQ6	Seed maturation protein PM34	71	17	31.7	7.08	0.0104
0.7454	A0A0R0JRB0	L-lactate dehydrogenase (EC 1.1.1.27)	51	10	37.8	7.02	0.0003
0.7383	C6TD56	Glyceraldehyde-3-phosphate dehydrogenase	69	17	37.0	7.47	0.0019
0.7377	I1JSN6	Usp domain-containing protein	24	2	19.9	6.33	0.0023
0.7333	I1LJN2	Uncharacterized protein	33	8	49.2	6.30	0.0004
0.7331	I1NBW7	Pescadillo homolog	8	2	69.2	8.84	0.0357
0.7329	C6TN05	Hypersensitive induced reaction protein 4	12	2	32.6	5.31	0.0055
0.7307	I1JVB1	Uncharacterized protein	38	2	10.3	10.05	0.0007
0.7279	I1KMB5	Uncharacterized protein	9	2	42.3	5.64	0.0192

0.7263	K7L479	J domain-containing protein	8	4	103.3	6.55	0.0075
0.7253	I1KDR0	Uncharacterized protein	64	16	57.1	5.39	0.0058
0.7200	C6T321	Clat_adaptor_s domain-containing protein	21	2	19.7	4.86	0.0153
0.7197	C6TB70	Thioredoxin domain-containing protein	56	7	24.4	6.92	0.0023
0.7197	A0A0R0EF73	Uncharacterized protein	8	8	183.5	6.18	0.0209
0.7195	I1JXA0	Uncharacterized protein	54	33	90.8	5.24	0.0012
0.7187	C6SWV3	Uncharacterized protein	66	11	27.6	6.10	0.0007
0.7161	B6ZJZ9	Peroxisomal adenine nucleotide carrier 1 (GmPNC1)	15	3	34.7	9.69	0.0125
0.7153	C6T9Z5	Formate dehydrogenase	58	15	42.8	6.77	0.0143
0.7108	I1KDI8	MI domain-containing protein	13	7	85.2	6.95	0.0145
0.7088	A0A0R4J309	Uncharacterized protein	37	5	18.6	10.51	0.0002
0.7074	C6SVF6	Glutaredoxin domain-containing protein	90	7	11.3	6.52	0.0003
0.7053	I1LD03	Dolichyl-diphosphooligosaccharide--protein glycosyltransferase subunit 1	29	9	52.7	7.31	0.0488
0.6991	I1M8B3	Uncharacterized protein	6	4	78.6	5.43	0.0423
0.6952	C6TK30	60S ribosomal protein L36	23	2	12.4	11.31	0.0003
0.6950	I1LP75	AAA domain-containing protein	7	4	68.4	9.29	0.0201
0.6908	I1M3A7	Uncharacterized protein	12	3	63.8	5.73	0.0044
0.6872	C6T470	Uncharacterized protein	16	2	27.2	4.81	0.0218
0.6868	C6TFN8	Uncharacterized protein	12	2	20.9	8.46	0.0108
0.6860	I1LCQ1	Pyruvate kinase (EC 2.7.1.40)	36	14	57.8	7.36	0.0003
0.6852	I1KQG0	T-complex protein 1 subunit delta	66	22	57.6	7.28	0.0318
0.6839	I1NHP3	Uncharacterized protein	25	2	15.6	9.09	0.0033
0.6829	I1LTT3	Uncharacterized protein	89	5	11.2	4.37	0.0066
0.6816	C6SWA6	Histone H2A	46	4	15.7	10.67	0.0355
0.6799	I1KSL3	Uncharacterized protein	7	3	89.9	5.55	0.0126
0.6779	I1K1V3	Uncharacterized protein	16	2	17.3	5.30	0.0394
0.6764	I1NBP5	Glucose-6-phosphate 1-dehydrogenase (EC 1.1.1.49)	5	2	68.0	6.84	0.0043
0.6759	K7MBG3	Sucrose synthase (EC 2.4.1.13)	27	17	91.5	6.24	0.0089
0.6756	Q84ZV2	Uncharacterized protein	33	2	11.3	9.13	0.0135
0.6745	Q9MAZ9	Nonclathrin coat protein zeta1-COP	46	4	20.1	4.86	0.0061
0.6732	I1MY42	Uncharacterized protein	2	2	135.6	7.58	0.0403
0.6731	A0A0R0HN41	Uncharacterized protein	61	4	12.0	4.46	0.0085
0.6721	I1M5H3	Nuclear pore complex protein NUP35 (Nucleoporin 35)	21	3	36.2	9.23	0.0226
0.6705	I1L0S5	Uncharacterized protein	40	5	19.0	6.93	0.0115
0.6673	C6SW70	Uncharacterized protein	42	11	28.2	9.88	0.0420
0.6627	I1NDE9	Uncharacterized protein	24	8	54.4	4.73	0.0131
0.6602	C6T4V0	Uncharacterized protein	60	2	8.0	4.53	0.0187
0.6592	I1J8U6	AAA domain-containing protein	8	3	70.3	8.10	0.0131
0.6578	K7MNV4	Uncharacterized protein	21	3	22.1	5.22	0.0007
0.6578	I1M860	TOG domain-containing protein	16	4	37.8	5.31	0.0387
0.6563	I1KVZ9	Bet_v_1 domain-containing protein	70	9	17.6	5.68	0.0139
0.6555	I1MT10	Uncharacterized protein	54	26	70.8	5.39	0.0015
0.6548	I1JR04	Tr-type G domain-containing protein	6	3	77.1	8.19	0.0451
0.6502	I1MUN3	Nop domain-containing protein	33	13	61.1	8.43	0.0075
0.6489	A0A0R0I4F6	Uncharacterized protein	56	15	31.8	7.36	0.0171
0.6483	I1KQ89	PMEI domain-containing protein	23	3	22.8	5.57	0.0044
0.6469	C6T588	Bet_v_1 domain-containing protein	61	7	16.8	4.88	0.0075
0.6319	A0A0R0L1W4	Glycosyltransferase (EC 2.4.1.-)	16	5	56.7	6.58	0.0393
0.6317	C6T2R8	Uncharacterized protein	25	5	25.0	5.57	0.0027
0.6306	K7KUE2	Uncharacterized protein	20	7	83.7	4.98	0.0480
0.6299	I1LD65	Dihydrolipoamide acetyltransferase component of pyruvate dehydrogenase complex (EC 2.3.1.-)	34	9	48.9	8.82	0.0434
0.6253	I1JZL5	Flavin-containing monooxygenase (EC 1.-.-.-)	35	9	52.2	5.63	0.0266
0.6227	I1N8H9	Uncharacterized protein	4	4	148.8	4.35	0.0340
0.6183	I1LA46	Uncharacterized protein	25	5	30.6	6.68	0.0026
0.6157	I1N5S0	Formate dehydrogenase	72	17	42.8	6.77	0.0008

0.6154	C6SVE0	Eukaryotic translation initiation factor 5A (eIF-5A)	30	4	17.4	5.76	0.0441
0.6142	I1J7G5	Uncharacterized protein	47	5	18.6	5.02	0.0014
0.6084	I1MRJ8	Uncharacterized protein	14	8	78.9	8.68	0.0127
0.6024	C6TDW7	Uncharacterized protein	15	4	25.5	8.16	0.0003
0.6009	I1M5Q4	ADP,ATP carrier protein	17	7	67.5	9.50	0.0436
0.6007	C6SVD3	Peptidylprolyl isomerase (EC 5.2.1.8)	23	2	12.0	6.01	0.0089
0.5988	I1M595	Uncharacterized protein	17	2	19.8	9.50	0.0448
0.5983	I1MMM3	Uncharacterized protein	30	4	23.3	7.11	0.0029
0.5970	I1KRB3	FAD-binding PCMH-type domain-containing protein	8	4	64.0	7.31	0.0201
0.5970	I1MCM4	RanBD1 domain-containing protein	14	4	48.1	4.91	0.0287
0.5941	A0A2Z6BYW7	Glycosyltransferase	54	16	53.6	6.16	0.0018
0.5936	I1L1F3	Uncharacterized protein	38	7	28.3	9.83	0.0053
0.5918	I1M984	AA_TRNA_LIGASE_II domain-containing protein	45	19	60.2	6.10	0.0189
0.5913	I1LS05	Uncharacterized protein	34	7	45.8	7.02	0.0165
0.5901	I1JQD8	UBA domain-containing protein	14	3	45.2	7.21	0.0289
0.5852	K7KMV4	Uncharacterized protein	2	2	124.2	5.91	0.0224
-0.5872	C6SYK2	Uncharacterized protein	38	5	22.4	5.07	0.0112
-0.5894	I1J940	RPW8 domain-containing protein	6	3	94.5	6.51	0.0337
-0.5915	A0A0R0J0J8	Uncharacterized protein	25	3	30.1	7.49	0.0041
-0.5929	I1LJE0	Adenylyl cyclase-associated protein	28	8	50.6	6.80	0.0326
-0.5930	A0A0R0KIF2	Importin N-terminal domain-containing protein	35	16	86.6	4.75	0.0009
-0.5931	A0A368UHG2	NAD(P)-bd_dom domain-containing protein	59	13	38.7	7.90	0.0168
-0.5936	I1L4U2	Serine hydroxymethyltransferase (EC 2.1.2.1)	55	15	57.0	7.59	0.0498
-0.5942	I1JRY5	Uncharacterized protein	20	5	37.4	9.55	0.0220
-0.5952	I1JXR7	Exostosin domain-containing protein	12	3	47.1	6.65	0.0062
-0.5961	C6SVK3	PITH domain-containing protein	21	2	22.7	5.27	0.0301
-0.5997	C6T8I2	Aminotran_1_2 domain-containing protein	38	11	43.6	5.77	0.0401
-0.6004	I1KJR7	Uncharacterized protein	17	3	28.4	4.86	0.0004
-0.6006	K7LW22	Ubiquitin-like domain-containing protein	4	3	116.7	7.56	0.0176
-0.6035	I1JZM5	Uncharacterized protein	7	2	51.5	6.76	0.0103
-0.6043	C6SW65	Uncharacterized protein	24	3	20.1	8.70	0.0233
-0.6054	I1NBW2	Serine/threonine-protein phosphatase (EC 3.1.3.16)	32	7	42.7	7.78	0.0274
-0.6078	I1KP26	Mg-por_mtran_C domain-containing protein	11	3	35.2	7.44	0.0249
-0.6083	Q2PMU2	Photosystem I P700 chlorophyll a apoprotein A2	19	9	82.4	7.31	0.0380
-0.6086	K7M8I6	Uncharacterized protein	6	3	60.9	6.55	0.0382
-0.6088	C6TJL7	Xyloglucan endotransglucosylase/hydrolase	21	4	34.2	8.21	0.0251
-0.6101	C6SY33	Uncharacterized protein	11	2	25.9	8.51	0.0006
-0.6114	C6THB9	Uncharacterized protein	43	12	45.6	6.35	0.0069
-0.6119	I1JJV5	Protein kinase domain-containing protein	28	9	57.9	6.18	0.0467
-0.6159	Q8S5C1	4-coumarate:CoA ligase isoenzyme 2 (EC 6.2.1.12)	27	11	60.1	6.74	0.0262
-0.6159	C6TMK2	Uncharacterized protein	17	3	31.9	9.26	0.0034
-0.6181	A0A0R0KVT1	Uncharacterized protein	39	5	26.1	5.80	0.0046
-0.6184	A0A0R0JQA9	X8 domain-containing protein	19	2	21.5	7.24	0.0270
-0.6214	A0A0R4J3N6	Uncharacterized protein	6	2	41.3	5.34	0.0042
-0.6229	I1L5K6	Carboxypeptidase (EC 3.4.16.-)	22	6	55.6	6.71	0.0027
-0.6237	I1KZI6	ENTH domain-containing protein	7	3	98.5	5.87	0.0294
-0.6251	I1K5J8	Uncharacterized protein	26	10	69.3	7.87	0.0103
-0.6269	I1K380	Uncharacterized protein	13	3	40.2	7.24	0.0291
-0.6275	I1L295	Uncharacterized protein	4	2	60.1	6.64	0.0326
-0.6289	I1JST6	RING-type E3 ubiquitin transferase (EC 2.3.2.27)	10	5	89.2	5.31	0.0016
-0.6291	Q1WCP1	Cytochrome P450 monooxygenase CYP94D24	6	2	39.3	9.22	0.0289
-0.6316	C6TL49	Protein kinase domain-containing protein	13	4	33.9	8.13	0.0061
-0.6329	I1K6N8	ThiF domain-containing protein	17	3	36.8	5.86	0.0031
-0.6338	C6TNQ1	Fe2OG dioxygenase domain-containing protein	41	8	34.9	5.31	0.0038
-0.6341	I1M9P8	X8 domain-containing protein	12	3	53.0	6.77	0.0061
-0.6361	I1LST9	Uncharacterized protein	9	4	66.7	6.29	0.0035
-0.6368	A0A0R0H975	Apocytochr_F_N domain-containing protein	34	7	35.3	8.82	0.0072

-0.6415	A0A0R0GFB2	Uncharacterized protein	54	18	57.5	8.87	0.0016
-0.6458	C6SVL3	Phytocyanin domain-containing protein	16	2	19.3	6.93	0.0003
-0.6470	I1L5G4	Amidophosphoribosyltransferase amidotransferase	27	11	62.0	6.84	0.0017
-0.6485	I1KM16	Uncharacterized protein	64	13	28.1	5.68	0.0128
-0.6500	C6T8H5	Tubulin gamma chain	11	3	53.1	5.94	0.0097
-0.6515	I1MVC1	Uncharacterized protein	41	15	69.4	8.35	0.0022
-0.6529	A0A0R0EQE5	Uncharacterized protein	33	16	70.3	6.04	0.0027
-0.6549	I1JR44	Uncharacterized protein	20	3	18.6	6.80	0.0394
-0.6550	I1J4T0	t-SNARE coiled-coil homology protein	12	2	30.1	6.10	0.0371
-0.6551	I1MVN5	Peroxidase (EC 1.11.1.7)	66	13	35.5	9.00	0.0128
-0.6574	C6TL29	Phosphoribulokinase (EC 2.7.1.19)	23	6	45.3	6.28	0.0139
-0.6593	C6T2H1	Flavodoxin-like domain-containing protein	57	8	21.7	6.95	0.0235
-0.6602	I1LI96	Phosphoserine aminotransferase (EC 2.6.1.52)	32	10	45.0	7.94	0.0096
-0.6607	A0A0R4J307	Uncharacterized protein	17	3	25.4	6.51	0.0141
-0.6639	I1L367	Uncharacterized protein	46	12	55.7	6.34	0.0331
-0.6653	I1J547	Aminotran_1_2 domain-containing protein	35	11	53.5	5.69	0.0012
-0.6654	C6T2V7	Phytocyanin domain-containing protein	18	2	26.1	4.87	0.0368
-0.6710	I1KUQ6	Lipoxygenase (EC 1.13.11.-)	26	15	97.1	7.40	0.0223
-0.6711	I1MU50	ENTH domain-containing protein	16	4	60.8	6.29	0.0121
-0.6733	I1K7Q8	NmrA domain-containing protein	60	12	34.2	6.06	0.0277
-0.6758	I1LQ56	Flavodoxin-like domain-containing protein	26	5	27.1	5.99	0.0176
-0.6763	A0A0R0LB64	Uncharacterized protein	44	10	34.2	9.29	0.0308
-0.6769	I1JL58	PRA1 family protein	11	2	24.2	8.19	0.0352
-0.6774	I1LRA7	Carboxypeptidase (EC 3.4.16.-)	28	7	51.0	5.52	0.0159
-0.6789	I1K498	Ferredoxin	29	3	15.8	4.55	0.0376
-0.6791	I1LU10	Uncharacterized protein	46	13	46.3	6.30	0.0018
-0.6800	A0A0R0KVX2	Fe2OG dioxygenase domain-containing protein	15	3	39.7	6.84	0.0162
-0.6806	K7K5P1	J domain-containing protein	31	5	27.1	5.50	0.0010
-0.6824	A0A368UKJ9	Pyruvate kinase (EC 2.7.1.40)	34	12	63.3	7.59	0.0299
-0.6848	I1MUR9	GMC_OxRdtase_N domain-containing protein	6	3	62.9	6.84	0.0212
-0.6848	K7MMN7	FAD-binding PCMH-type domain-containing protein	7	4	66.1	8.07	0.0023
-0.6892	I1J4P0	TFIIS N-terminal domain-containing protein	5	2	57.6	5.38	0.0135
-0.6894	I1K5M5	AP-3 complex subunit delta	4	3	108.7	6.42	0.0047
-0.6902	A0A0R0HC62	Uncharacterized protein	13	2	20.4	8.43	0.0037
-0.6944	I1JJP2	Glucose-1-phosphate adenylyltransferase	35	11	56.3	6.74	0.0228
-0.6945	A0A0R4J5R0	Fe2OG dioxygenase domain-containing protein	19	4	37.0	5.88	0.0015
-0.6966	I1MR46	Uncharacterized protein	23	8	62.0	9.41	0.0447
-0.7031	I1K4M6	C2 domain-containing protein	28	36	229.2	5.36	0.0470
-0.7036	A0A0R0KAJ9	Uncharacterized protein	6	3	58.8	6.96	0.0197
-0.7124	I1JGA0	Calcium-transporting ATPase (EC 7.2.2.10)	16	10	113.9	6.11	0.0000
-0.7137	I1K675	TPR_REGION domain-containing protein	16	7	61.5	5.40	0.0226
-0.7182	C6ZS11	Leucine-rich repeat resistance protein-like protein	12	3	37.1	8.38	0.0368
-0.7242	K7MKC7	SRP54 domain-containing protein	14	5	68.7	8.18	0.0000
-0.7244	A0A0R0ESN9	Uncharacterized protein	26	10	76.4	5.07	0.0042
-0.7263	I1LCX7	RING-type E3 ubiquitin transferase (EC 2.3.2.27)	4	2	68.7	6.05	0.0016
-0.7311	I1JQZ6	Pept_C1 domain-containing protein	27	6	39.7	6.27	0.0043
-0.7320	A0A0R0HNL5	NAB domain-containing protein	2	4	251.2	4.97	0.0040
-0.7335	I1L2T0	SGL domain-containing protein	10	2	41.3	6.46	0.0076
-0.7417	I1JJW5	Uncharacterized protein	13	4	61.5	5.83	0.0036
-0.7419	I1M5C1	LEA_2 domain-containing protein	12	2	27.8	10.23	0.0062
-0.7501	Q9ZNZ5	Peroxidase (EC 1.11.1.7) (Fragment)	36	8	38.5	8.13	0.0314
-0.7505	I1LSM1	Phytocyanin domain-containing protein	20	3	22.8	6.92	0.0040
-0.7508	A0A1V0PL25	Argonaute 5	8	4	103.3	9.57	0.0278
-0.7510	I1LWX5	Uncharacterized protein	21	6	54.2	7.42	0.0196
-0.7512	I1LFX6	Uncharacterized protein	20	11	96.9	4.89	0.0232
-0.7530	A0A0R0GAK0	Uncharacterized protein	48	10	30.6	8.40	0.0007
-0.7564	I1M6B9	Neutral ceramidase (EC 3.5.1.23)	28	13	84.5	7.24	0.0004

-0.7575	I1KMS7	SMK-1 domain-containing protein	7	4	100.0	5.39	0.0002
-0.7583	C6T7J2	Uncharacterized protein	12	3	25.1	9.01	0.0018
-0.7624	I1M712	Oxygen-evolving enhancer protein 2	31	4	28.6	7.87	0.0067
-0.7648	A0A0R0LAT1	Protein kinase domain-containing protein	10	5	66.4	7.50	0.0223
-0.7653	K7MU41	Uncharacterized protein	26	10	66.9	8.53	0.0000
-0.7671	K7MYB6	HP domain-containing protein	33	21	108.4	5.71	0.0031
-0.7693	K7ML47	Phytocyanin domain-containing protein	17	2	25.3	9.72	0.0466
-0.7737	I1N1F9	Aldedh domain-containing protein	56	18	58.3	7.15	0.0006
-0.7737	A0A0R0JCW2	Uncharacterized protein	26	7	66.2	6.29	0.0308
-0.7740	A0A0R0HXE9	Uncharacterized protein	12	2	18.7	9.99	0.0206
-0.7764	I1JSJ3	Fructose-bisphosphate aldolase (EC 4.1.2.13)	30	8	42.5	6.79	0.0334
-0.7793	I1KJQ4	Reticulon-like protein	9	3	62.4	8.41	0.0209
-0.7800	A0A0R0LI21	Uncharacterized protein	2	2	232.4	5.64	0.0444
-0.7801	I1JBJ9	Endoglucanase (EC 3.2.1.4)	8	2	56.6	9.22	0.0053
-0.7834	C6TK14	FBPase domain-containing protein	10	3	41.7	6.43	0.0482
-0.7836	I1KHK4	Protein kinase domain-containing protein	11	4	77.5	6.20	0.0083
-0.7843	C6TDP2	Uncharacterized protein	30	8	30.1	7.93	0.0010
-0.7881	C6TKD0	Fe2OG dioxygenase domain-containing protein	18	2	40.9	6.37	0.0033
-0.7917	A0A0R4J4D2	PKS_ER domain-containing protein	56	13	39.1	5.95	0.0012
-0.7952	K7MK51	Uncharacterized protein	7	4	87.6	6.98	0.0306
-0.7974	I1MRL3	TPR_REGION domain-containing protein	7	2	45.4	7.28	0.0278
-0.7980	I1L327	DUF862 domain-containing protein	19	3	27.2	7.34	0.0489
-0.7995	I1MJG6	Uncharacterized protein	12	14	180.4	7.55	0.0154
-0.7999	A0A0R0JPA1	Beta-galactosidase (EC 3.2.1.23)	26	13	94.0	7.53	0.0011
-0.7999	K7L6W0	LRRNT_2 domain-containing protein	12	3	37.9	5.81	0.0019
-0.8025	K7MR59	NAD(P)-bd_dom domain-containing protein	26	8	74.3	6.67	0.0263
-0.8032	I1NHN6	Uncharacterized protein	10	3	66.2	8.91	0.0061
-0.8055	K7MCL5	Flavin-containing monooxygenase	14	4	59.3	7.83	0.0015
-0.8071	I1KW78	Uncharacterized protein	23	8	60.8	9.16	0.0079
-0.8089	K7LKM1	Peptidase_M28 domain-containing protein	10	5	99.7	7.62	0.0154
-0.8102	C6TNJ3	S-adenosylmethionine synthase (EC 2.5.1.6)	63	13	42.9	6.14	0.0094
-0.8108	K7LEA7	RING-type domain-containing protein	23	5	39.1	4.60	0.0024
-0.8143	A0A0R0IM08	Uncharacterized protein	34	5	30.9	8.41	0.0426
-0.8148	I1MGZ2	Plasma membrane ATPase (EC 7.1.2.1)	40	29	105.0	6.81	0.0037
-0.8163	A0A0R0FLF0	Xyloglucan endotransglucosylase/hydrolase	25	8	40.4	9.25	0.0048
-0.8182	I1LCP7	Protein kinase domain-containing protein	7	2	54.8	7.21	0.0165
-0.8186	C6TGB8	Uncharacterized protein	36	10	46.2	6.10	0.0038
-0.8233	C6TBY6	V-type proton ATPase proteolipid subunit	29	2	16.5	8.46	0.0002
-0.8245	C6TI82	Methyltransferase (EC 2.1.1.-)	33	9	38.5	6.65	0.0301
-0.8311	K7LXR3	Uncharacterized protein	6	2	83.1	6.95	0.0018
-0.8327	I1LIV5	FAD-binding PCMH-type domain-containing protein	47	19	66.0	8.21	0.0011
-0.8340	I1L2C2	Transmembrane 9 superfamily member	21	10	74.9	6.18	0.0010
-0.8364	K7KQQ0	Uncharacterized protein	13	2	30.8	7.28	0.0191
-0.8402	I1N842	Xyloglucan endotransglucosylase/hydrolase	28	7	34.1	7.71	0.0195
-0.8403	I1JM18	Transket_pyr domain-containing protein	41	7	39.0	6.54	0.0006
-0.8404	C6T225	Uncharacterized protein	24	4	22.2	5.00	0.0002
-0.8405	I1LP66	Uncharacterized protein	17	9	96.7	4.82	0.0197
-0.8411	I1L4V4	Fn3_like domain-containing protein	24	12	85.1	5.71	0.0216
-0.8423	K7N544	CMP/dCMP-type deaminase domain-containing protein	26	3	20.6	5.16	0.0113
-0.8451	K7L413	Hexosyltransferase (EC 2.4.1.-)	6	3	75.9	8.92	0.0189
-0.8469	A0A0R0JYS9	Peroxidase (EC 1.11.1.7)	60	12	35.5	8.76	0.0177
-0.8629	K7MNN1	Aminotran_1_2 domain-containing protein	12	3	52.8	7.94	0.0077
-0.8647	I1JSR9	MFS domain-containing protein	12	5	78.7	5.72	0.0012
-0.8689	C6TEX7	Uncharacterized protein	16	3	28.9	5.96	0.0000
-0.8698	I1K4S4	Uncharacterized protein	17	3	35.2	9.54	0.0260
-0.8710	I1KFE9	Uncharacterized protein	30	9	60.1	7.77	0.0022
-0.8765	C6T3D7	G protein gamma domain-containing protein	35	3	16.0	5.71	0.0258

-0.8786	C6TNE6	Chlorophyll a-b binding protein, chloroplastic	53	6	27.9	5.43	0.0203
-0.8810	I1K0L4	Uncharacterized protein	34	15	71.7	6.00	0.0055
-0.8837	C6TFS5	Cytochrome b6-f complex iron-sulfur subunit	32	5	28.4	8.79	0.0111
-0.8865	K7KKK3	Uncharacterized protein	18	4	61.0	7.44	0.0053
-0.8872	I1L776	Uncharacterized protein	35	6	39.9	5.29	0.0127
-0.8879	Q06765	ADR6 protein (Sali5-4a protein)	52	8	30.4	6.84	0.0039
-0.8880	B0FPA3	Extracellular Ca2+ sensing receptor	10	2	41.9	9.39	0.0269
-0.8895	A0A0R4J3B7	Lipase_GDSL domain-containing protein	23	4	39.7	4.84	0.0171
-0.8914	K7LD67	Uncharacterized protein	67	18	41.7	5.49	0.0072
-0.8921	A0A0R0IIS3	Cas1_AcylT domain-containing protein	4	2	67.1	8.81	0.0004
-0.8924	A0A368ULH0	Kinesin-like protein	3	2	98.3	7.40	0.0449
-0.8937	A0A0R0JYY8	Tubulin beta chain	55	22	49.9	5.01	0.0050
-0.8966	I1M4U9	NAD(P)-bd_dom domain-containing protein	51	11	39.1	8.13	0.0295
-0.8982	I1JY06	Uncharacterized protein	33	17	91.7	5.31	0.0003
-0.9041	I1KIJ3	Uncharacterized protein	3	2	117.3	7.12	0.0088
-0.9073	I1JHV7	Uncharacterized protein	10	2	29.7	7.64	0.0003
-0.9107	A0A0R4J3V9	Flavodoxin-like domain-containing protein	46	6	21.7	6.54	0.0037
-0.9119	I1L340	C2 domain-containing protein	15	3	30.3	5.03	0.0046
-0.9144	K7M429	Uncharacterized protein	30	10	45.6	6.28	0.0020
-0.9218	A0A0R4J339	Uncharacterized protein	18	6	82.3	8.54	0.0079
-0.9234	Q6RUF9	Proline dehydrogenase (EC 1.5.5.2)	9	3	54.4	8.13	0.0047
-0.9283	I1KPM9	TPR_REGION domain-containing protein	17	8	63.2	5.41	0.0023
-0.9291	I1L8N6	Uncharacterized protein	8	2	50.0	5.78	0.0064
-0.9332	I1KTW4	ALA-interacting subunit	9	2	38.5	9.54	0.0084
-0.9357	A0A0R0K666	Peptidase A1 domain-containing protein	45	9	47.0	8.12	0.0207
-0.9398	I1MC85	Uncharacterized protein	32	10	45.8	5.82	0.0006
-0.9409	G0T437	Purple acid phosphatase (EC 3.1.3.2)	17	7	74.1	6.35	0.0013
-0.9512	C6TH50	Xyloglucan endotransglucosylase/hydrolase	22	5	35.0	5.01	0.0015
-0.9561	I1KN20	Uncharacterized protein	6	2	54.8	5.15	0.0261
-0.9630	I1JG92	Uncharacterized protein	12	5	86.3	6.23	0.0102
-0.9636	I1LLM2	Asparagine synthetase	51	19	65.2	6.64	0.0019
-0.9657	C6T487	Uncharacterized protein	19	3	19.8	7.09	0.0205
-0.9680	I1MYV1	C2 domain-containing protein	32	41	228.5	5.50	0.0270
-0.9697	I1MDX7	Uncharacterized protein	14	3	31.9	8.15	0.0123
-0.9706	I1JTX2	Uncharacterized protein	16	3	28.2	9.83	0.0001
-0.9720	I1L6Y8	Protein kinase domain-containing protein	21	11	98.0	6.01	0.0006
-0.9723	I1KBK8	Uncharacterized protein	23	13	91.0	5.25	0.0128
-0.9725	I1MBL5	Uncharacterized protein	40	15	57.9	8.75	0.0187
-0.9758	I1K8M2	Transmembrane 9 superfamily member	20	9	73.2	7.61	0.0300
-0.9772	I1MQP2	Cellulase domain-containing protein	7	3	56.2	5.62	0.0005
-0.9863	I1LZN1	Transmembrane 9 superfamily member	17	8	77.5	6.96	0.0290
-0.9909	I1LPL0	FAD-binding PCMH-type domain-containing protein	47	19	66.0	8.22	0.0045
-0.9936	A0A0R4J645	Uncharacterized protein	9	2	41.6	5.45	0.0126
-0.9960	A0A0R0K0E5	FAD-binding PCMH-type domain-containing protein	10	4	59.2	7.85	0.0015
-0.9977	I1MPK1	Uncharacterized protein	6	2	49.9	5.58	0.0012
-0.9993	C6SXF0	Uncharacterized protein	23	3	22.6	10.23	0.0039
-1.0009	I1KG34	Uncharacterized protein	74	32	68.7	5.50	0.0200
-1.0047	C6THA9	NAD(P)-bd_dom domain-containing protein	51	11	39.1	8.13	0.0272
-1.0052	A0A0R0G3L8	Phospho-2-dehydro-3-deoxyheptonate aldolase	22	9	54.9	8.28	0.0100
-1.0065	Q39828	Dynamin-related protein 5A	26	13	68.3	7.99	0.0198
-1.0099	I1KP02	Uncharacterized protein	27	11	65.3	5.05	0.0007
-1.0105	K7M5U1	GH10 domain-containing protein	13	9	103.8	6.95	0.0031
-1.0125	K7MLF2	AAA domain-containing protein	12	8	132.6	6.61	0.0003
-1.0137	C6SY48	Glutathione peroxidase	26	3	18.9	9.07	0.0070
-1.0160	I1K3H5	Lactamase_B domain-containing protein	11	2	34.8	6.57	0.0219
-1.0181	I1KJZ0	E3 ubiquitin-protein ligase (EC 2.3.2.27)	2	2	229.8	6.25	0.0252
-1.0200	I1LWZ5	PMR5N domain-containing protein	7	2	50.4	6.84	0.0341

-1.0210	K7KRR7	X8 domain-containing protein	10	3	54.4	5.97	0.0306
-1.0235	I1KEH1	Phytocyanin domain-containing protein	20	3	23.2	7.87	0.0001
-1.0237	I1M9W9	Uncharacterized protein	4	2	107.0	7.27	0.0157
-1.0258	I1N6I1	Hexosyltransferase (EC 2.4.1.-)	8	3	63.1	8.48	0.0024
-1.0314	C6T1T3	Uncharacterized protein	35	4	22.4	6.07	0.0061
-1.0320	I1KYC6	Peptidase A1 domain-containing protein	14	4	51.9	7.84	0.0101
-1.0327	I1J4W3	Uncharacterized protein	10	4	73.1	7.23	0.0468
-1.0344	I1M1I9	Uncharacterized protein	5	3	82.0	9.28	0.0017
-1.0392	I1LVI0	Protein kinase domain-containing protein	10	5	100.6	7.25	0.0395
-1.0420	I1ND63	LRRNT_2 domain-containing protein	21	7	50.3	8.05	0.0131
-1.0428	I1M9S1	Plasma membrane ATPase (EC 7.1.2.1)	32	22	104.6	7.21	0.0013
-1.0483	I1MA39	Lipase_GDSL domain-containing protein	13	3	46.8	8.19	0.0301
-1.0483	A0A0R0I2M6	Peroxidase (EC 1.11.1.7)	34	5	38.5	6.04	0.0114
-1.0506	C6TI50	Peroxidase (EC 1.11.1.7)	31	5	38.0	8.43	0.0220
-1.0557	I1LLG3	Peptidase A1 domain-containing protein	29	6	46.8	9.01	0.0010
-1.0565	I1MHW2	Uncharacterized protein	4	3	94.2	6.64	0.0046
-1.0567	A0A0R4J2V8	Dirigent protein	28	3	23.9	6.28	0.0027
-1.0571	A0A0R0L0K1	Uncharacterized protein	10	4	62.4	9.07	0.0098
-1.0572	I1M9K9	Uncharacterized protein	25	8	39.8	7.74	0.0076
-1.0579	A0A0R0HFK7	Exostosin domain-containing protein	5	2	59.4	8.84	0.0081
-1.0621	I1MEL2	Uncharacterized protein	31	2	13.3	8.81	0.0086
-1.0643	I1LJG0	Glycosyltransferase (EC 2.4.2.-)	22	6	49.4	8.78	0.0028
-1.0643	C6SX09	Uncharacterized protein	34	7	29.2	6.35	0.0066
-1.0693	A0A0R0JQ91	Chlorophyll a-b binding protein, chloroplastic	33	4	27.9	7.44	0.0366
-1.0728	I1NIY9	Mitochondrial Rho GTPase (EC 3.6.5.-)	43	16	71.9	5.54	0.0037
-1.0749	I1LQ25	Glucose-1-phosphate adenylyltransferase	13	4	58.5	8.29	0.0130
-1.0807	C6TJF9	Uncharacterized protein	55	8	24.0	6.58	0.0014
-1.0811	I1MXA0	Uncharacterized protein	4	2	63.2	5.36	0.0029
-1.0826	C6SVL0	Uncharacterized protein	47	6	21.8	5.33	0.0043
-1.0969	K7MPZ4	dUTPase domain-containing protein	43	3	15.7	5.53	0.0045
-1.1072	C6T9R8	Glyceraldehyde-3-phosphate dehydrogenase	37	10	48.1	7.43	0.0235
-1.1073	I1LMX6	Uroporphyrinogen decarboxylase (EC 4.1.1.37)	25	5	42.7	7.49	0.0012
-1.1092	I1MY52	PKS_ER domain-containing protein	22	4	39.4	7.33	0.0195
-1.1108	I1K7G0	Methyltransferase (EC 2.1.1.-)	34	10	40.4	6.74	0.0001
-1.1108	I1MY38	HATPase_c domain-containing protein	38	28	93.2	4.98	0.0468
-1.1121	I1NBM7	Uncharacterized protein	32	10	55.5	6.73	0.0055
-1.1152	A0A0R0FQV9	Xyloglucan endotransglucosylase/hydrolase	28	7	33.7	6.93	0.0202
-1.1202	I1LLM7	Asparagine synthetase	51	19	65.2	6.64	0.0079
-1.1250	K7LQ40	Pyr_redox_2 domain-containing protein	9	2	39.4	8.54	0.0000
-1.1318	D2D5G3	Methyltransferase (EC 2.1.1.-)	31	9	40.4	6.95	0.0065
-1.1418	A0A0R0GAQ0	Peptidase A1 domain-containing protein	5	2	50.1	8.75	0.0080
-1.1497	I1NCT8	Potassium transporter	4	2	88.4	8.56	0.0018
-1.1536	I1K0I0	Uncharacterized protein	10	5	75.6	8.37	0.0183
-1.1598	I1L1D9	Uncharacterized protein	35	2	10.5	9.35	0.0310
-1.1673	C6TA28	Nucleoside diphosphate kinase (EC 2.7.4.6)	15	2	25.2	9.03	0.0074
-1.1673	C6SVC7	Uncharacterized protein	32	3	20.2	7.87	0.0003
-1.1714	C6F122	Beta-galactosidase (EC 3.2.1.23)	14	5	79.8	8.03	0.0421
-1.1738	I1LBD2	Uncharacterized protein	14	2	22.0	5.21	0.0187
-1.1770	A0A0R0GA40	Uncharacterized protein	13	3	50.8	6.01	0.0170
-1.1783	A0A0R0H2Z4	Uncharacterized protein	22	7	59.8	8.94	0.0247
-1.1817	I1J7R6	FACT complex subunit SSRP1	6	3	71.8	5.78	0.0329
-1.1833	K7N361	Lipase	11	3	49.2	7.23	0.0063
-1.1900	I1K108	Uncharacterized protein	52	18	69.1	7.93	0.0001
-1.1922	I1KET7	PMR5N domain-containing protein	8	2	40.6	8.94	0.0045
-1.1958	C6SYP7	Dirigent protein	18	2	24.2	8.37	0.0015
-1.2125	I1MBC5	Asparagine synthetase	57	21	65.5	6.47	0.0005
-1.2182	I1NAB2	Uncharacterized protein	9	8	146.2	7.62	0.0046

-1.2192	I1L6Z0	Cellulase domain-containing protein	7	3	55.5	5.64	0.0238
-1.2422	I1K6B1	NAB domain-containing protein	5	4	139.4	4.94	0.0078
-1.2507	I1J5A5	Aldedh domain-containing protein	50	16	58.4	6.84	0.0043
-1.2593	C6SZQ8	Phytocyanin domain-containing protein	24	2	17.3	6.76	0.0246
-1.2730	I1LRP4	Uncharacterized protein	27	8	60.2	7.28	0.0280
-1.2771	I1MLA1	Uncharacterized protein	6	2	55.0	8.94	0.0026
-1.2786	I1MTI7	Aldedh domain-containing protein	27	8	49.7	8.13	0.0022
-1.2803	I1KHL4	Chlorophyll a-b binding protein, chloroplastic	22	3	30.8	6.39	0.0228
-1.2808	I1L2Z2	SPX domain-containing protein	9	4	78.1	6.61	0.0026
-1.2818	I1MCZ5	LEA_2 domain-containing protein	12	2	27.9	10.27	0.0001
-1.2873	I1MHW9	Uncharacterized protein	12	4	59.1	6.11	0.0174
-1.2874	I1J5R4	Uncharacterized protein	15	2	25.9	5.07	0.0292
-1.2946	I1KQC0	Uncharacterized protein	15	6	68.8	8.95	0.0455
-1.3102	I1KRB5	FAD-binding PCMH-type domain-containing protein	33	12	58.8	8.70	0.0039
-1.3289	I1K510	Uncharacterized protein	18	8	93.7	5.17	0.0036
-1.3330	I1KMK2	Uncharacterized protein	4	3	162.5	8.13	0.0062
-1.3344	A8HTK0	Thioredoxin	51	4	13.3	5.53	0.0084
-1.3413	K7LQS4	Uncharacterized protein	5	2	72.1	6.54	0.0105
-1.3421	I1JWM6	COBRA-like protein	10	2	50.6	8.68	0.0377
-1.3427	I1MS92	Uncharacterized protein	5	2	55.0	8.28	0.0063
-1.3447	A8C8H5	Glutamate decarboxylase (EC 4.1.1.15)	59	19	57.2	5.82	0.0014
-1.3474	I1LZU4	CYTH domain-containing protein	13	2	22.3	5.39	0.0002
-1.3518	I1KBS7	Uncharacterized protein	39	12	62.5	4.86	0.0008
-1.3558	I1MQL3	Uncharacterized protein	18	14	116.4	9.31	0.0134
-1.3585	A0A0R0GN50	Phosphotransferase (EC 2.7.1.-)	29	9	53.7	5.24	0.0044
-1.3586	I1MZM7	Uncharacterized protein	27	4	26.7	6.42	0.0076
-1.3632	C6THI3	PKS_ER domain-containing protein	58	12	39.1	6.84	0.0102
-1.3635	I1MER4	PMR5N domain-containing protein	28	6	42.3	8.75	0.0015
-1.3677	K7KN46	Uncharacterized protein	2	2	239.3	5.77	0.0471
-1.3708	K7LP97	RRM domain-containing protein	42	5	16.9	8.12	0.0015
-1.3770	K7LCZ8	Cellulose synthase (EC 2.4.1.12)	20	11	120.1	7.39	0.0017
-1.3800	C6T447	Uncharacterized protein	40	6	25.1	6.35	0.0172
-1.3876	I1N4W1	PMR5N domain-containing protein	24	6	51.3	8.51	0.0006
-1.3922	I1KSC1	Uncharacterized protein	11	5	66.5	8.19	0.0001
-1.3923	A0A0R0EHS8	PMR5N domain-containing protein	33	3	20.5	8.31	0.0020
-1.3928	C6TI75	NTP_transferase domain-containing protein	22	5	39.6	7.49	0.0369
-1.4015	I1KNV8	Uncharacterized protein	14	7	95.1	5.14	0.0141
-1.4032	I1K702	PLCXc domain-containing protein	26	5	36.2	8.02	0.0072
-1.4111	I1KRH0	AAA domain-containing protein	9	2	45.4	6.29	0.0120
-1.4310	A0A0R0HUK1	Uncharacterized protein	18	3	30.7	7.42	0.0187
-1.4347	I1NII6	IPPc domain-containing protein	2	2	127.2	6.47	0.0300
-1.4531	K7MP10	Uncharacterized protein	10	6	118.6	5.02	0.0112
-1.4605	A0A0R0FVX1	Uncharacterized protein	32	12	58.6	7.37	0.0001
-1.4696	I1L0D9	Peroxidase (EC 1.11.1.7)	36	6	38.0	7.30	0.0062
-1.4723	K7KZ57	Uncharacterized protein	5	2	51.6	6.42	0.0029
-1.4724	C6T736	V-SNARE domain-containing protein	42	6	24.7	9.50	0.0001
-1.4725	A0A0R0J452	Uncharacterized protein	19	3	25.2	6.15	0.0058
-1.4859	C6TMX0	Xyloglucan endotransglucosylase/hydrolase	31	8	34.4	8.95	0.0192
-1.5011	I1KZ04	Cytochrome b561 and DOMON protein	13	3	43.2	9.38	0.0018
-1.5060	I1MLV5	Fe2OG dioxygenase domain-containing protein	32	7	35.8	5.69	0.0481
-1.5145	K7LJA2	Uncharacterized protein	8	2	51.2	6.02	0.0005
-1.5155	I1LHR1	RRM domain-containing protein	8	2	57.6	8.41	0.0343
-1.5285	C6T3E5	MPN domain-containing protein	39	4	22.9	5.78	0.0203
-1.5380	I1M3B6	Uncharacterized protein	3	2	131.6	7.34	0.0003
-1.5532	I1M3S8	Uncharacterized protein	4	3	85.7	5.12	0.0062
-1.5570	I1KD34	COBRA-like protein	14	3	50.7	8.60	0.0002
-1.5571	I1L3E3	Uncharacterized protein	6	2	39.4	9.48	0.0010

-1.5647	Q8LGT8	Phosphoglycerate mutase-like protein	20	4	31.8	6.34	0.0488
-1.5938	A0A0R0IYY7	Uncharacterized protein	51	19	59.0	8.65	0.0001
-1.6086	K7K7V7	Pterin-binding domain-containing protein	15	5	57.4	7.42	0.0100
-1.6092	C6TD35	PABS domain-containing protein	9	2	37.0	5.40	0.0033
-1.6144	A0A0R0G7V6	Uncharacterized protein	5	2	62.8	6.99	0.0177
-1.6241	A0A0R4J508	Expansin	11	2	27.9	9.26	0.0015
-1.6335	I1JIW7	Uncharacterized protein	30	7	24.9	9.42	0.0024
-1.6454	I1LVS4	Uncharacterized protein	27	9	61.7	5.20	0.0127
-1.6549	C6TDZ7	Uncharacterized protein	14	3	40.2	6.19	0.0006
-1.6588	I1JT65	Uncharacterized protein	18	4	40.1	6.37	0.0001
-1.6799	C6THF2	Uncharacterized protein	14	2	31.5	5.36	0.0001
-1.6808	A0A0R0IA82	Peptidase A1 domain-containing protein	14	3	52.8	5.35	0.0012
-1.6826	A0A0R0KTP5	Uncharacterized protein	30	4	30.4	8.15	0.0494
-1.6847	A0A024FRX1	Glycosyltransferase (EC 2.4.1.-)	10	3	52.2	6.14	0.0003
-1.6949	I1MMC2	t-SNARE coiled-coil homology protein	18	3	30.0	6.32	0.0027
-1.7112	I1KTE1	Cellulose synthase (EC 2.4.1.12)	12	8	123.9	6.93	0.0009
-1.7140	I1MNI2	Uncharacterized protein	16	3	39.6	6.80	0.0080
-1.7162	A0A0R0KYU7	Xyloglucan endotransglucosylase/hydrolase	29	8	36.0	8.25	0.0000
-1.7217	A0A0R0INP8	Peroxidase (EC 1.11.1.7)	29	7	34.5	8.90	0.0017
-1.7267	I1LCV9	Carboxypeptidase (EC 3.4.16.-)	16	6	56.0	5.38	0.0025
-1.7448	C6SVM4	Uncharacterized protein	35	4	22.3	5.82	0.0041
-1.7567	I1LA99	Uncharacterized protein	10	4	86.2	5.92	0.0440
-1.7632	I1L7W1	Uncharacterized protein	4	4	167.6	8.15	0.0014
-1.7641	C6ZRR4	NAK-type protein kinase	8	5	102.2	6.34	0.0008
-1.7689	I1LL85	Uncharacterized protein	5	2	60.7	8.48	0.0003
-1.7749	I1K8B5	Uncharacterized protein	11	2	53.5	6.13	0.0074
-1.7803	Q8S564	4-coumarate:coenzyme A ligase (EC 6.2.1.12)	26	9	59.3	5.91	0.0004
-1.7845	I1JUE0	Cellulose synthase (EC 2.4.1.12)	28	19	122.0	6.76	0.0001
-1.7961	I1NAW5	Uncharacterized protein	15	3	50.5	6.71	0.0150
-1.7981	I1L0Z7	Uncharacterized protein	6	6	168.3	7.47	0.0002
-1.8001	C6SXG7	FMN_red domain-containing protein	63	7	21.5	8.82	0.0126
-1.8130	I1MWI1	zinc_ribbon_10 domain-containing protein	9	2	44.8	8.84	0.0012
-1.8142	I1MXX3	Uncharacterized protein	21	2	16.5	9.31	0.0023
-1.8150	C6TLB6	Uncharacterized protein	46	11	46.3	6.30	0.0010
-1.8279	C6TL20	Lipase_GDSL domain-containing protein	31	7	40.2	6.10	0.0017
-1.8295	I1LI35	Vacuolar protein sorting-associated protein 35	18	8	89.7	5.44	0.0008
-1.8427	C6SYN8	Uncharacterized protein	24	2	15.5	7.96	0.0423
-1.8565	I1K3A6	Uncharacterized protein	10	4	64.3	6.57	0.0001
-1.8580	I1NEV9	Uncharacterized protein	15	2	24.2	7.83	0.0160
-1.8758	I1L6R1	Uncharacterized protein	13	6	66.9	8.27	0.0249
-1.8945	A0A0R0KRN8	Glycosyltransferase (EC 2.4.1.-)	7	2	50.9	7.50	0.0010
-1.9108	I1JCY0	Chlorophyll a-b binding protein, chloroplastic	18	3	26.7	6.68	0.0153
-1.9271	C6SZJ5	FAS1 domain-containing protein	14	2	26.3	9.57	0.0026
-1.9278	A0A0R0HZ32	Uncharacterized protein	13	2	20.0	5.30	0.0000
-1.9318	I1MVV4	Uncharacterized protein	3	2	125.9	5.22	0.0067
-1.9357	I1JVD1	Uncharacterized protein	6	3	78.7	6.73	0.0165
-1.9358	I1ND18	Uncharacterized protein	9	3	73.8	6.83	0.0056
-1.9361	A0A0R4J551	Phytocyanin domain-containing protein	19	2	20.9	7.36	0.0361
-1.9599	K7KP88	Uncharacterized protein	3	5	255.0	6.07	0.0129
-1.9635	Q2LAL4	Cytochrome P450 monooxygenase CYP83E8	38	13	57.4	7.96	0.0017
-1.9707	I1KH95	Uncharacterized protein	5	2	93.0	8.76	0.0011
-1.9772	I1K8W4	Cellulose synthase (EC 2.4.1.12)	28	18	122.0	6.76	0.0004
-1.9853	I1LDY8	Uncharacterized protein	23	5	52.1	7.80	0.0011
-2.0002	I1M192	Uncharacterized protein	26	5	33.3	5.81	0.0048
-2.0075	C6TEQ7	Lipase_GDSL domain-containing protein	15	2	37.8	7.85	0.0078
-2.0174	A0A0R0FGJ1	Protein kinase domain-containing protein	4	2	95.1	6.23	0.0092

-2.0339	K7MNL1	AB hydrolase-1 domain-containing protein	10	2	29.9	6.21	0.0258
-2.0362	Q2LAL0	Cytochrome P450 monooxygenase CYP81E11	22	6	57.9	8.91	0.0107
-2.0459	C6T8K0	LEA_2 domain-containing protein	19	4	34.2	9.94	0.0001
-2.0550	I1LH27	Tubulin alpha chain	56	20	49.7	5.06	0.0019
-2.0794	I1KWS8	Glyco_hydro_19_cat domain-containing protein	10	2	36.0	6.27	0.0004
-2.0933	K7M4N7	Uncharacterized protein	4	2	65.1	8.12	0.0002
-2.1012	A0A0R0HPC7	Pectate lyase (EC 4.2.2.2)	5	2	42.1	8.09	0.0004
-2.1108	I1JRU5	Lipase_GDSL domain-containing protein	25	6	40.4	5.74	0.0008
-2.1334	C6TJK9	Peptidyl-prolyl cis-trans isomerase	10	2	28.3	8.60	0.0005
-2.1461	I1K3E9	CULLIN_2 domain-containing protein	10	6	88.5	7.65	0.0166
-2.2140	I1N843	Glyceraldehyde-3-phosphate dehydrogenase	47	14	43.2	8.25	0.0000
-2.2167	I1LB67	t-SNARE coiled-coil homology protein	10	2	36.3	8.81	0.0194
-2.2268	I1NHU7	Uncharacterized protein	10	2	39.0	6.29	0.0278
-2.2439	A0A0R0EP22	Uncharacterized protein	14	6	76.5	7.87	0.0237
-2.2451	A0A0R0L0I9	HTH myb-type domain-containing protein	2	2	46.7	6.84	0.0076
-2.2454	I1MI08	Transmembrane 9 superfamily member	21	10	74.9	5.88	0.0014
-2.2574	I1KNP5	Endoglucanase (EC 3.2.1.4)	9	4	68.7	8.72	0.0427
-2.2622	K7L6B3	Uncharacterized protein	13	2	31.1	6.93	0.0008
-2.3237	K7LYU8	Uncharacterized protein	7	2	66.9	7.18	0.0196
-2.3297	I1MVM2	Uncharacterized protein	5	2	69.8	6.38	0.0381
-2.3618	C6SZD1	UBIQUITIN_CONJUGAT_2 protein	11	2	18.1	5.39	0.0053
-2.3838	I1MZV7	Uncharacterized protein	5	2	49.6	8.68	0.0095
-2.4066	I1MY41	Uncharacterized protein	13	5	61.3	6.65	0.0047
-2.4512	I1L2J3	Uncharacterized protein	16	5	66.7	7.18	0.0217
-2.4562	K7MNZ5	Uncharacterized protein	15	3	40.4	6.70	0.0006
-2.4712	A0A0R0IPL8	NAD(P)-bd_dom domain-containing protein	35	7	38.5	6.54	0.0421
-2.4892	I1MFX5	Endoglucanase (EC 3.2.1.4)	13	6	68.3	8.66	0.0003
-2.4906	A0A0R0LJE6	Uncharacterized protein	4	2	83.1	7.11	0.0220
-2.4911	A0A0R0I5R9	Uncharacterized protein	38	12	48.0	6.52	0.0005
-2.5087	Q9XFI6	Peroxidase (EC 1.11.1.7)	23	5	37.4	8.35	0.0021
-2.5298	I1K2X6	FAD-binding PCMH-type domain-containing protein	30	11	59.5	9.03	0.0339
-2.5372	K7M1F7	Lipase_GDSL domain-containing protein	8	2	40.5	7.91	0.0005
-2.6118	Q2MJR4	Myo-inositol-1-phosphate synthase	40	9	56.4	5.67	0.0061
-2.6285	I1K6Q2	Uncharacterized protein	12	3	48.3	5.78	0.0166
-2.6332	A0A368UNA6	SSD domain-containing protein	14	10	138.7	5.22	0.0095
-2.6470	I1L1D2	Uncharacterized protein	6	3	88.5	7.15	0.0414
-2.6623	Q70MR6	Ornithine decarboxylase (EC 4.1.1.17)	9	2	46.4	5.49	0.0103
-2.6754	I1L158	Cellulose synthase (EC 2.4.1.12)	6	3	119.2	7.62	0.0154
-2.6970	I1JFT2	Glycosyltransferase (EC 2.4.1.-)	6	2	54.9	6.52	0.0009
-2.7072	A0A0R0HMK4	Uncharacterized protein	8	2	46.1	7.50	0.0003
-2.7144	I1K0W4	Glutamate dehydrogenase	17	4	44.8	6.61	0.0259
-2.7338	C6KHU4	Phenylalanine ammonia-lyase (EC 4.3.1.24)	41	16	78.1	6.19	0.0001
-2.7677	K7MQS3	Peptidase A1 domain-containing protein	16	3	52.0	8.12	0.0003
-2.7941	I1NGM0	Uncharacterized protein	5	2	75.1	9.01	0.0009
-2.8082	I1M8S3	Uncharacterized protein	36	15	82.1	7.39	0.0009
-2.8427	I1KG57	Uncharacterized protein	32	9	46.6	5.88	0.0109
-2.8776	I1J785	Uncharacterized protein	25	8	52.0	7.78	0.0194
-2.9483	I1N641	Uncharacterized protein	16	14	136.1	7.99	0.0001
-2.9488	A0A0R4J3Q3	ABC transporter domain-containing protein	33	4	28.3	5.90	0.0232
-3.0120	I1KLU0	Mitogen-activated protein kinase (EC 2.7.11.24)	27	7	49.9	5.96	0.0000
-3.0368	I1L5J4	Uncharacterized protein	7	2	45.5	8.72	0.0218
-3.0375	C6TK45	Uncharacterized protein	8	2	43.8	8.46	0.0005
-3.0905	I1MAU6	Uncharacterized protein	8	4	78.8	6.55	0.0198
-3.1094	C6TH72	Fe2OG dioxygenase domain-containing protein	25	4	32.9	5.43	0.0431
-3.1585	C6TD22	Glyco_hydro_19_cat domain-containing protein	15	3	36.4	6.51	0.0001
-3.2153	I1M1T9	ALOG domain-containing protein	20	3	20.2	9.72	0.0047
-3.2262	C6T3V3	Glutathione peroxidase	18	3	19.1	9.61	0.0001

-3.2293	I1MC22	Lipase_GDSL domain-containing protein	10	2	39.9	7.85	0.0413
-3.2428	I1L090	Endoglucanase (EC 3.2.1.4)	13	6	68.3	8.68	0.0001
-3.2597	A0A024FRL5	Glycosyltransferase (EC 2.4.1.-)	8	2	52.0	5.92	0.0050
-3.3298	A0A0R0JYG7	Protein kinase domain-containing protein	11	4	76.1	6.54	0.0065
-3.3321	I1ML02	Uncharacterized protein	8	4	69.9	6.62	0.0006
-3.3431	I1N0K6	WD_REPEATS_REGION domain-containing protein	43	6	32.6	6.16	0.0323
-3.3501	I1JQB4	Uncharacterized protein	15	3	50.1	6.46	0.0092
-3.3546	I1KYC4	Peptidase A1 domain-containing protein	15	4	51.9	8.07	0.0013
-3.3578	I1N6V6	PPR_long domain-containing protein	11	3	50.7	7.42	0.0060
-3.3939	I1N019	Uncharacterized protein	12	4	72.2	6.25	0.0020
-3.3941	I1JSP4	X8 domain-containing protein	10	2	48.3	5.16	0.0008
-3.4054	I1JBJ2	Uncharacterized protein	45	11	50.2	6.99	0.0387
-3.4989	I1LRC1	Uncharacterized protein	21	9	62.5	9.11	0.0001
-3.5022	A0A0R4J4M3	FAS1 domain-containing protein	13	2	27.8	8.88	0.0194
-3.5037	A0A0R0KUR1	Phenylalanine ammonia-lyase (EC 4.3.1.24)	20	9	77.9	6.42	0.0001
-3.5069	I1MXD5	Uncharacterized protein	6	2	53.2	5.58	0.0018
-3.6505	I1JT08	Uncharacterized protein	10	3	81.4	6.67	0.0086
-3.6567	I1MVG7	Uncharacterized protein	23	7	76.9	4.35	0.0221
-3.7044	A0A0R4J2X1	Uncharacterized protein	8	2	44.0	8.34	0.0005
-3.7556	A0A0R0HVZ4	Exocyst subunit Exo70 family protein	5	2	72.0	6.99	0.0033
-3.7919	I1MXJ1	Uncharacterized protein	36	15	82.2	7.09	0.0001
-3.7953	C6TKJ2	Xyloglucan endotransglucosylase/hydrolase	35	7	35.6	8.37	0.0010
-3.8034	I1M5G8	Malic enzyme	27	9	71.3	7.55	0.0270
-3.8111	K7KP00	Uncharacterized protein	18	3	25.2	9.41	0.0039
-3.8424	I1LWJ6	Uncharacterized protein	17	14	136.0	7.99	0.0000
-3.8971	A0A0R0IBM9	CMP/dCMP-type deaminase domain-containing protein	19	3	20.7	5.86	0.0031
-4.0931	Q70MR5	Ornithine decarboxylase (EC 4.1.1.17)	9	2	46.3	5.60	0.0021
-4.1630	I1JDD7	Cellulose synthase (EC 2.4.1.12)	11	7	121.4	7.18	0.0163
-4.2009	I1MLF6	Uncharacterized protein	5	2	73.9	5.45	0.0125
-4.3376	I1NA96	Phenylalanine ammonia-lyase (EC 4.3.1.24)	28	11	77.7	6.49	0.0012
-4.4031	I1KI92	PlsC domain-containing protein	9	3	55.5	9.23	0.0000
-4.5513	I1N0I7	t-SNARE coiled-coil homology protein	16	3	28.1	8.90	0.0001
-4.6020	I1LVD2	Cellulose synthase (EC 2.4.1.12)	13	7	120.5	7.08	0.0003
-4.7092	I1KPJ3	Endoglucanase (EC 3.2.1.4)	6	3	68.7	8.72	0.0034
-4.8318	I1LZ94	Phenylalanine ammonia-lyase (EC 4.3.1.24)	38	15	78.1	6.10	0.0036
-5.0603	I1M8D6	X8 domain-containing protein	7	2	51.2	5.19	0.0321
-5.3458	A0A0R0I8A1	Xyloglucan endotransglucosylase/hydrolase	41	7	29.9	7.96	0.0009
-5.8235	A0A0R0FIC1	Flavodoxin-like domain-containing protein	4	2	71.6	6.92	0.0001
-5.8610	C6TKY7	Str_synth domain-containing protein	8	2	38.8	7.81	0.0001
-6.0312	I1LKW4	Uncharacterized protein	21	9	62.4	9.11	0.0008
-6.1644	A0A0R0GCM7	CMP/dCMP-type deaminase domain-containing protein	19	3	20.6	6.13	0.0002
-6.6578	I1K2B5	Uncharacterized protein	20	9	79.6	6.60	0.0024
-7.3191	I1LJF7	MFS domain-containing protein	8	3	56.5	9.38	0.0009
-10.4654	A0A0R0EIR6	Glycosyltransferase	36	9	54.4	5.92	0.0000

Abbreviation means as follows: Cov, coverage; MP, matched protein; MW, molecular weight; Cal, calculated.
Grey background is to proteins not considered to be differentially accumulated.
