# Supplementary material for: Proteomic and Biochemical Analyses of the Mechanism of Tolerance in Mutant Soybean Responding to Flooding Stress
Source: Int J Mol Sci. 2021 Aug 22;22(16):9046. doi: 10.3390/ijms22169046 (PMC8396653; doi:10.3390/ijms22169046)
Supplement: Supplementary file 1 [file ijms-22-09046-s001.zip › Supplementary Tables S3 and S4.pdf]

Supplemental Table S3. List of changed proteins in mutant line compared with wild type soybean under non-flooding

Fold change	Accession	Protein names	Cov (%)	MP	MW (kDa)	calc pI	p-value
6.2056	C6TMB5	Uncharacterized protein	45	5	17.8	8.97	0.0013
5.8848	C6T9K9	Elongation factor Tu	49	17	49.8	7.05	0.0002
5.6934	I1KMK2	Uncharacterized protein	4	3	162.5	8.13	0.0015
4.7650	I1KZW3	Uncharacterized protein	32	6	32.8	5.92	0.0006
4.5582	I1MV69	Uncharacterized protein	25	4	27.5	9.2	0.0042
4.3471	I1KBD1	Protein kinase domain-containing protein	4	2	129.2	6.74	0.0034
4.1682	C6SYA5	60S ribosomal protein L18a	29	4	21.3	10.42	0.0006
3.6515	A0A0R4J4G7	Uncharacterized protein	15	2	29.4	6.79	0.0017
3.4478	I1LVI3	Uncharacterized protein	12	9	100.1	9.07	0.0037
3.3423	A0A0R0GL62	PRA1 family protein	22	2	23.1	9.82	0.0004
3.3384	I1KQE2	Uncharacterized protein	59	9	34.6	7.81	0.0126
3.0367	A0A0R0I304	Uncharacterized protein	8	2	57.6	8.94	0.0405
2.9024	I1JJ62	Citrate synthase (EC 2.3.3.16)	19	5	56.5	8.72	0.0285
2.8070	C6TMH1	ZnMc domain-containing protein	12	2	40.1	5.63	0.0193
2.7239	I1NCN7	Uncharacterized protein	5	3	115.7	6.46	0.0125
2.7003	C6TKW5	MHD domain-containing protein	7	2	49.7	7.18	0.0038
2.6352	I1KJ00	Uncharacterized protein	14	3	28.2	5.43	0.0334
2.4269	A0A0R0KF48	Uncharacterized protein	41	14	65.2	6.21	0.0005
2.3746	Q1WCP1	Cytochrome P450 monooxygenase CYP94D24	6	2	39.3	9.22	0.0012
2.3474	I1LRQ2	NEDD8-activating enzyme E1 regulatory subunit	21	5	57.9	5.24	0.0388
2.3034	I1KLU0	Mitogen-activated protein kinase (EC 2.7.11.24)	27	7	49.9	5.96	0.0309
2.3008	K7KRK2	Uncharacterized protein	17	3	31.5	6.44	0.0265
2.2760	I1NGM0	Uncharacterized protein	5	2	75.1	9.01	0.0018
2.2590	I1LMC1	RRM domain-containing protein	10	2	31.7	11.41	0.0369
2.0603	C6T721	Aldose 1-epimerase (EC 5.1.3.3) (Galactose mutarotase)	13	3	40.6	9.28	0.0178
1.9815	I1L7R7	CTP synthase (EC 6.3.4.2) (UTP--ammonia ligase)	9	3	61.6	6.05	0.0007
1.9552	K7KU37	Uncharacterized protein	2	2	147.2	5.94	0.0106
1.8579	I1LWX5	Uncharacterized protein	21	6	54.2	7.42	0.0172
1.7970	I1JET8	Uncharacterized protein	6	2	56.9	6.48	0.0121
1.7764	K7LF33	RBR-type E3 ubiquitin transferase (EC 2.3.2.31)	10	3	68.2	5.26	0.0049
1.7466	I1MRW2	Importin N-terminal domain-containing protein	5	4	123.7	4.83	0.0006
1.6853	C6TIP6	Uncharacterized protein	10	3	54.5	8.25	0.0492
1.6347	K7LSJ2	Importin N-terminal domain-containing protein	24	17	123.9	5.57	0.0132
1.4224	A0A0R0EP22	Uncharacterized protein	14	6	76.5	7.87	0.0055
1.3771	I1NB50	Uncharacterized protein	7	2	52.8	7.9	0.0258
1.3363	I1N3Y0	Uncharacterized protein	15	4	46.1	7.05	0.0003
1.2035	A0A0R0KFE8	Chlorophyll a-b binding protein, chloroplastic	27	5	31.4	6.32	0.0008
1.1934	I1LM96	60S ribosomal export protein NMD3	8	3	58.5	6.73	0.0356
1.1687	A0A0R0IH39	Uncharacterized protein	2	2	212.1	7.31	0.0270
1.1259	A0A0R0EM40	Uncharacterized protein	8	3	57.1	8.63	0.0250
1.1152	K7MFQ7	Uncharacterized protein	15	6	68.8	8.5	0.0137
1.1091	I1LJG3	Flavodoxin-like domain-containing protein	25	4	27.3	6	0.0399
1.0995	K7MNN1	Aminotran_1_2 domain-containing protein	12	3	52.8	7.94	0.0004
1.0771	I1K072	Uncharacterized protein	33	16	77.3	5.14	0.0052
1.0701	I1NAS0	EF-hand domain-containing protein	9	3	47.3	5.49	0.0233
1.0662	I1K6B1	NAB domain-containing protein	5	4	139.4	4.94	0.0418
1.0491	K7M034	BED-type domain-containing protein	8	6	122.7	5	0.0331
1.0433	A0A0R0JDS4	TP_methylase domain-containing protein	24	2	30.8	5.39	0.0495

0.9923	C6T065	Ribosomal_S17_N domain-containing protein	31	4	17.8	10.56	0.0173
0.9801	I1L0D9	Peroxidase (EC 1.11.1.7)	36	6	38	7.3	0.0262
0.9699	I1JK97	Protein kinase domain-containing protein	19	6	55.1	5.99	0.0097
0.9108	I1MY43	Vacuolar protein sorting-associated protein 35	15	7	90.3	5.43	0.0012
0.8914	A0A0R0FN05	Gamma-tubulin complex component	7	3	75.3	7.11	0.0056
0.8697	A0A0R0F5Y7	WD_REPEATS_REGION domain-containing protein	18	14	121.9	5.52	0.0016
0.8570	K7LD67	Uncharacterized protein	67	18	41.7	5.49	0.0106
0.8547	C6TFJ4	Uncharacterized protein	38	3	15.3	8.13	0.0424
0.8269	Q39834	Clathrin heavy chain	45	55	193.2	5.55	0.0050
0.8230	I1KSA4	Uncharacterized protein	24	2	19.4	5.67	0.0467
0.8031	K7LXR3	Uncharacterized protein	6	2	83.1	6.95	0.0465
0.8024	K7MFC0	GYF domain-containing protein	5	7	179.2	6.23	0.0070
0.7973	I1KY70	Uncharacterized protein	5	2	86	6.02	0.0461
0.7577	I1M7P3	Uncharacterized protein	34	2	14.7	4.93	0.0013
0.7497	K7LK00	Uncharacterized protein	4	2	83.4	6.04	0.0059
0.7371	I1N1M0	ATP-dependent 6-phosphofructokinase (ATP-PFK) (Phosphofructokinase) (EC 2.7.1.11) (Phosphohexokinase)	13	4	58.4	7.59	0.0301
0.7351	I1LPZ3	V-type proton ATPase subunit a	27	15	92.9	5.96	0.0383
0.7234	I1J547	Aminotran_1_2 domain-containing protein	35	11	53.5	5.69	0.0021
0.7136	I1LQR8	Uncharacterized protein	34	5	26.2	7.85	0.0189
0.7040	I1K108	Uncharacterized protein	52	18	69.1	7.93	0.0007
0.6986	I1LGI3	RanBD1 domain-containing protein	8	2	39.1	7.18	0.0007
0.6906	I1LLG6	Uncharacterized protein	5	4	179.7	6.14	0.0129
0.6572	C6T3E5	MPN domain-containing protein	39	4	22.9	5.78	0.0122
0.6489	K7L9D8	Kinesin motor domain-containing protein	2	2	143.1	6.16	0.0263
0.6375	I1L090	Endoglucanase (EC 3.2.1.4)	13	6	68.3	8.68	0.0206
0.6353	I1JMK6	Uncharacterized protein	16	3	52.2	5.81	0.0078
0.6304	I1LF85	RING-type domain-containing protein	14	2	31.8	4.75	0.0246
0.6188	I1JMY9	Uncharacterized protein	20	5	48.9	6.3	0.0428
0.6125	C5NTE6	Uncharacterized protein	52	8	34.1	8.66	0.0338
0.5934	I1MVM6	Protein kinase domain-containing protein	20	5	40.6	8.66	0.0238
0.5863	I1LL06	Carboxypeptidase (EC 3.4.16.-)	17	5	54.6	6.76	0.0035
-0.5958	I1MXD1	Uncharacterized protein	25	12	69.1	5.88	0.0405
-0.6015	C6T374	VOC domain-containing protein	61	4	15.4	5.69	0.0091
-0.6030	I1N8M4	NAC-A/B domain-containing protein	45	6	22	4.55	0.0030
-0.6068	I1NDE9	Uncharacterized protein	24	8	54.4	4.73	0.0021
-0.6151	C6SX29	ACT domain-containing protein	22	2	16.6	4.7	0.0022
-0.6160	I1KB60	Uncharacterized protein	10	2	60	5.22	0.0340
-0.6489	I1LD65	Dihydrolipoamide acetyltransferase component of pyruvate dehydrogenase complex (EC 2.3.1.-)	34	9	48.9	8.82	0.0421
-0.6571	Q43468	Hsp70-Hsp90 organizing protein 1 (GmHop-1)	17	7	64.1	5.69	0.0347
-0.6769	K7MVZ6	Glucose-6-phosphate isomerase (EC 5.3.1.9)	37	10	62.7	5.72	0.0469
-0.6783	A0A0R0L3G5	Amino_oxidase domain-containing protein	5	3	66.8	7.87	0.0268
-0.6870	C6T394	UBIQUITIN_CONJUGAT_2 domain-containing protein	11	2	17.9	5.07	0.0050
-0.6887	I1JXR7	Exostosin domain-containing protein	12	3	47.1	6.65	0.0012
-0.6961	K7KCU6	Protein kinase domain-containing protein	6	3	92	8.16	0.0447
-0.7225	I1K1I6	Isocitrate dehydrogenase [NADP] (EC 1.1.1.42)	30	13	53.3	8.12	0.0158
-0.7306	C6SWA6	Histone H2A	46	4	15.7	10.67	0.0140
-0.7332	C6SV65	Histone H2A	44	3	14.7	10.36	0.0069
-0.7615	I1K042	Uncharacterized protein	6	2	66.5	9.95	0.0127
-0.7905	A0A0R0EWR2	PCI domain-containing protein	12	4	52.9	6.76	0.0080
-0.7933	Q8RVH5	Basic 7S globulin 2 (SBg7S) (Bg)	40	8	47.2	8.25	0.0419

-0.8351	M1FIS9	Ribosomal protein S4	11	3	41.3	11.22	0.0297
-0.8426	K7LDT9	Uncharacterized protein	9	5	103.3	6.76	0.0271
-0.9010	C6SXR9	Uncharacterized protein	15	3	26.1	8.05	0.0078
-0.9039	I1KYS8	Uncharacterized protein	12	3	41.1	7.85	0.0113
-0.9336	C6TN86	Uncharacterized protein	10	2	38.3	6	0.0223
-0.9470	I1JN48	DNA gyrase subunit B (EC 5.6.2.3)	7	3	81.1	8.38	0.0060
-0.9739	I1LRK0	DUF1279 domain-containing protein	15	2	20.4	9.41	0.0431
-1.0228	I1MDY6	Uncharacterized protein	5	2	60.6	9.51	0.0010
-1.0284	I1KKH2	Importin N-terminal domain-containing protein	6	3	114.8	5.68	0.0076
-1.1939	C6TE68	Uncharacterized protein	11	2	26.5	7.2	0.0307
-1.1947	A6BM07	Isoflavone 7-O-glucosyltransferase 1	11	3	52	5.34	0.0114
-1.2152	I1N1G8	PKS_ER domain-containing protein	14	4	41.9	8.94	0.0229
-1.3584	A0A0R0KL81	Uncharacterized protein	7	3	80.9	7.14	0.0037
-1.4020	A0A0R0G7V6	Uncharacterized protein	5	2	62.8	6.99	0.0105
-1.5596	I1LZW4	Tr-type G domain-containing protein	16	11	109.9	5.24	0.0104
-1.5825	K7M549	APC1_C domain-containing protein	3	2	201.2	6.27	0.0421
-1.6023	I1JSX4	AAA domain-containing protein	18	8	74.1	6.19	0.0010
-1.6412	I1L8V8	Uncharacterized protein	45	5	17.7	8.7	0.0114
-1.7599	A0A0R0HAG1	Uncharacterized protein	3	2	120.9	5.83	0.0048
-1.7742	I1LYM0	DUF1681 domain-containing protein	23	4	30.7	6.68	0.0084
-1.9360	K7K3E3	Uncharacterized protein	4	3	147.9	5.27	0.0134
-1.9552	A0A0R0I9A1	3-ketoacyl-CoA synthase (EC 2.3.1.-)	14	4	56.1	8.9	0.0416
-2.2526	I1LHJ6	SHNi-TPR domain-containing protein	23	6	50.1	4.54	0.0000
-2.2675	C6T390	Uncharacterized protein	55	6	16.2	5.91	0.0122
-2.3826	K7KVD9	Uncharacterized protein	4	2	73.5	6.42	0.0035
-2.6470	I1MFV0	Uncharacterized protein	11	9	152.5	6.19	0.0165
-4.1073	C6T020	Glutathione peroxidase	33	3	18.7	5.72	0.0129
-4.3460	K7K8V7	C2 domain-containing protein	2	3	229.9	5.62	0.0028
-5.8382	K7MPG5	Uncharacterized protein	28	6	42.4	8.29	0.0003

Abbreviation means as follows: Cov, coverage; MP, matched protein; MW, molecular weight; Cal, calculated.
Grey background is to proteins not considered to be differentially accumulated.

Supplemental Table S4. List of changed proteins in mutant line compared with wild type soybean under flooding

Fold change	Accession	Protein names	Cov (%)	MP	MW (kDa)	calc pI	p-value
3.3229	C6T2R5	RNase H domain-containing protein	14	2	26.7	7.34	0.0017
3.2103	I1JZK8	t-SNARE coiled-coil homology domain-containing protein	10	2	33.3	7.14	0.0000
3.1314	K7N4G1	Uncharacterized protein	33	10	46.6	6.79	0.0408
3.0888	I1NGD0	Lipoxygenase (EC 1.13.11.-)	12	6	97.9	6.7	0.0396
2.6669	K7MQS3	Peptidase A1 domain-containing protein	16	3	52	8.12	0.0011
2.2582	A0A0R0KUR1	Phenylalanine ammonia-lyase (EC 4.3.1.24)	20	9	77.9	6.42	0.0070
2.0875	C6T2W1	SHSP domain-containing protein	24	3	21.9	6.71	0.0285
1.9052	I1K6B1	NAB domain-containing protein	5	4	139.4	4.94	0.0469
1.6802	I1J7C0	Uncharacterized protein	11	2	37.3	4.88	0.0035
1.6645	I1MXD5	Uncharacterized protein	6	2	53.2	5.58	0.0376
1.5914	I1LM80	Sucrose synthase (EC 2.4.1.13)	5	3	95.7	6.99	0.0354
1.5010	I1LRM9	Uncharacterized protein	12	2	50.4	7.08	0.0368
1.4174	I1L0J1	Uncharacterized protein	5	2	52.9	5.16	0.0002
1.3421	I1JK18	Translocase of chloroplast (EC 3.6.5.-)	23	4	34.9	9.33	0.0456
1.2353	A0A0R0FUC5	Uncharacterized protein	21	3	21.4	5.29	0.0135
1.2302	I1KZ04	Cytochrome b561 and DOMON domain-containing protein	13	3	43.2	9.38	0.0019
1.1109	A8C8H5	Glutamate decarboxylase (EC 4.1.1.15)	59	19	57.2	5.82	0.0114
1.0686	I1M8S3	Uncharacterized protein	36	15	82.1	7.39	0.0290
1.0161	A0A0R0GIU4	Peroxidase (EC 1.11.1.7)	24	5	35.2	7.83	0.0179
0.9979	C6SZQ8	Phytocyanin domain-containing protein	24	2	17.3	6.76	0.0333
0.9801	I1L3X9	Sucrose synthase (EC 2.4.1.13)	11	6	104.1	7.06	0.0483
0.9769	I1KM99	Uncharacterized protein	30	8	54.6	4.68	0.0043
0.9042	I1MHW9	Uncharacterized protein	12	4	59.1	6.11	0.0322
0.8976	I1KMW1	Uncharacterized protein	23	3	27.6	6.02	0.0185
0.8642	I1MX49	Uncharacterized protein	22	6	46	6.35	0.0055
0.8383	K7M8I6	Uncharacterized protein	6	3	60.9	6.55	0.0339
0.8057	I1MMC2	t-SNARE coiled-coil homology domain-containing protein	18	3	30	6.32	0.0369
0.7693	I1KMW4	TPR_REGION domain-containing protein	12	4	50	8.18	0.0013
0.7260	K7KQQ0	Uncharacterized protein	13	2	30.8	7.28	0.0450
0.6872	I1J414	Uncharacterized protein	8	3	38.4	5.86	0.0273
0.6297	I1LF63	Importin N-terminal domain-containing protein	30	14	99.4	5.05	0.0258
0.6210	K7L479	J domain-containing protein	8	4	103.3	6.55	0.0483
0.6141	C6SXC9	Prefoldin subunit 4	34	3	14.7	4.46	0.0325
-0.6020	C6THF2	Uncharacterized protein	14	2	31.5	5.36	0.0074
-0.6234	C6SVK3	PITH domain-containing protein	21	2	22.7	5.27	0.0401
-0.6262	I1LLG3	Peptidase A1 domain-containing protein	29	6	46.8	9.01	0.0064
-0.6307	A0A0R0KFR5	Uncharacterized protein	13	2	31.6	9.55	0.0266
-0.6356	A0A0R0HN41	Uncharacterized protein (Fragment)	61	4	12	4.46	0.0370
-0.6470	I1J5A5	Aldedh domain-containing protein	50	16	58.4	6.84	0.0405
-0.6534	I1N5M3	Elongation factor Ts, mitochondrial (EF-Ts) (EF-TsMt)	7	2	38.3	8.24	0.0008
-0.6609	C6FF81	Receptor-like serine/threonine kinase	7	6	146.9	7.65	0.0329
-0.7104	A0A0R0ICF9	Pectinesterase (EC 3.1.1.11)	20	2	35.4	6.34	0.0175
-0.7245	I1K108	Uncharacterized protein	52	18	69.1	7.93	0.0004
-0.7455	I1KN20	Uncharacterized protein	6	2	54.8	5.15	0.0476
-0.7619	I1N842	Xyloglucan endotransglucosylase/hydrolase (EC 2.4.1.207)	28	7	34.1	7.71	0.0315
-0.7811	K7LKM1	Peptidase_M28 domain-containing protein	10	5	99.7	7.62	0.0057
-0.8115	K7MAX3	Uncharacterized protein	2	2	186.9	6.9	0.0324

-0.8146	I1M4L7	Beta-galactosidase (EC 3.2.1.23)	12	5	91.6	6.76	0.0037
-0.8344	I1KJ00	Uncharacterized protein	14	3	28.2	5.43	0.0080
-0.8413	A0A0R4J508	Expansin	11	2	27.9	9.26	0.0401
-0.8540	C6SX09	Uncharacterized protein (Fragment)	34	7	29.2	6.35	0.0068
-0.8553	M1FIP8	NADH-ubiquinone oxidoreductase chain 5 (EC 7.1.1.2)	9	4	74	7.84	0.0212
-0.8553	I1MCF0	Uncharacterized protein	23	5	45.2	6.43	0.0220
-0.8602	C6SYC0	Ribosomal_L18e/L15P domain-containing protein	16	3	27.6	10.99	0.0064
-0.8715	I1MY52	PKS_ER domain-containing protein	22	4	39.4	7.33	0.0322
-0.9106	C6TMZ4	Fe2OG dioxygenase domain-containing protein	30	5	36	5.38	0.0348
-0.9261	K7MJA4	Uncharacterized protein	1	2	186.8	7.14	0.0248
-1.0106	C6TNM5	Uncharacterized protein	17	3	36.4	7.17	0.0291
-1.0379	I1N9W1	Uncharacterized protein	5	5	161.4	8.87	0.0078
-1.0807	K7MNN1	Aminotran_1_2 domain-containing protein	12	3	52.8	7.94	0.0025
-1.1474	C6SXF0	Uncharacterized protein	23	3	22.6	10.23	0.0028
-1.1716	I1M192	Uncharacterized protein	26	5	33.3	5.81	0.0246
-1.3723	I1J730	Peptidylprolyl isomerase (EC 5.2.1.8)	18	7	69.9	5.57	0.0075
-1.4347	Q8RVH5	Basic 7S globulin 2 (SBg7S) (Bg) [Cleaved into: Basic 7S globulin 2 high kDa subunit;Basic 7S globulin 2 low kDa subunit]	40	8	47.2	8.25	0.0215
-1.4648	I1JNW2	tRNA (guanine-N(7)-)-methyltransferase non-catalytic subunit (WD repeat-containing protein 4 homolog)	16	3	44.7	5.91	0.0010
-1.4756	C6T3E5	MPN domain-containing protein	39	4	22.9	5.78	0.0204
-1.5000	I1JQZ9	Uncharacterized protein	10	3	83.9	7.23	0.0421
-1.5599	Q2MJR4	Myo-inositol-1-phosphate synthase	40	9	56.4	5.67	0.0239
-1.5804	I1MNI2	Uncharacterized protein	16	3	39.6	6.8	0.0033
-1.7512	C6TJK9	Peptidyl-prolyl cis-trans isomerase (PPIase) (EC 5.2.1.8)	10	2	28.3	8.6	0.0022
-1.8013	Q8LGT8	Phosphoglycerate mutase-like protein	20	4	31.8	6.34	0.0144
-1.8176	I1MZV7	Uncharacterized protein	5	2	49.6	8.68	0.0251
-1.8636	A0A0R0HMK4	Uncharacterized protein	8	2	46.1	7.5	0.0365
-2.0019	I1KG57	Uncharacterized protein	32	9	46.6	5.88	0.0414
-2.0281	K7LYU8	Uncharacterized protein	7	2	66.9	7.18	0.0316
-2.0787	P26413	Heat shock 70 kDa protein	53	25	70.8	5.49	0.0073
-2.2675	A0A0R0LJE6	Uncharacterized protein	4	2	83.1	7.11	0.0280
-2.4074	I1MTI4	Protein kinase domain-containing protein	8	2	43.7	7.65	0.0454
-2.6402	I1K3E9	CULLIN_2 domain-containing protein	10	6	88.5	7.65	0.0029
-2.8521	C6TKV3	DHO_dh domain-containing protein	35	9	46.1	5.96	0.0090
-2.8558	I1M1T9	ALOG domain-containing protein	20	3	20.2	9.72	0.0078
-3.1430	I1KLU0	Mitogen-activated protein kinase (EC 2.7.11.24)	27	7	49.9	5.96	0.0013
-3.5487	C6TJZ9	Uncharacterized protein	16	5	38.7	5.01	0.0015
-6.1028	I1K2B5	Uncharacterized protein	20	9	79.6	6.6	0.0033
-6.7510	I1LEX5	Uncharacterized protein	28	6	42.9	4.84	0.0002

Abbreviation means as follows: Cov, coverage; MP, matched protein; MW, molecular weight; Cal, calculated.
Grey background is to proteins not considered to be differentially accumulated.
